# Application of Green Chiral Chromatography in Enantioseparation of Newly Synthesized Racemic Marinoepoxides

**DOI:** 10.3390/md20080530

**Published:** 2022-08-19

**Authors:** Anđela Buljan, Marin Roje

**Affiliations:** Ruđer Bošković Institute, Department of Organic Chemistry and Biochemistry, Laboratory for Chiral Technologies, Bijenička cesta 54, 10 000 Zagreb, Croatia

**Keywords:** green solvents, chiral chromatography, marinoepoxides, marinoaziridines, 2,3-disubstituted epoxides, 2,3,3-trisubstituted epoxides, enantioseparation, supercritical fluid chromatography (SFC), high-performance liquid chromatography (HPLC), supercritical carbon dioxide, dimethyl carbonate (DMC)

## Abstract

Enantioseparation of the newly synthesized series of novel quinoline-2(1*H*)-one epoxide structures *rac*-**6a**–**c** and *rac*-**8a**–**c**, named marinoepoxides, is described. Marinoepoxide *rac*-**6a**, the key intermediate in the total synthesis of natural products marinoaziridines A and B, as well as their structural analogues, was synthesized by addition of the achiral ylide generated in situ from the sulfonium salt **5** or **7**, to the carbon-oxygen double bond of the corresponding quinoline-2(1*H*)-one-4-carbaldehyde **4a**–**c** in good yield. Separation of enantiomers of (±)-2,3,3-trisubstituted marinoepoxides *rac*-**6a**–**c** and (±)-*trans*-2,3-disubstituted marinoepoxides *rac*-**8a**–**c** was studied using two immobilized polysaccharide type chiral stationary phases (CSPs); *tris*-(3,5-dichlorophenylcarbamoyl)cellulose stationary phase (CHIRAL ART Cellulose-SC) and *tris-*(3,5-dimethylphenylcarbamoyl)amylose stationary phase (CHIRAL ART Amylose-SA). Enantioseparation conditions were explored by high-performance liquid chromatography (HPLC) using dimethyl carbonate/alcohol mixtures and *n*-hexane/ethanol (80/20, *v*/*v*) as mobile phase, and by supercritical fluid chromatography (SFC) using CO_2_/alcohol mixtures as mobile phase. In all examined racemates, enantioseparation was successfully achieved, but its efficiency largely depended on the structure of chiral selector and type/composition of the mobile phase.

## 1. Introduction

**Figure 1:**
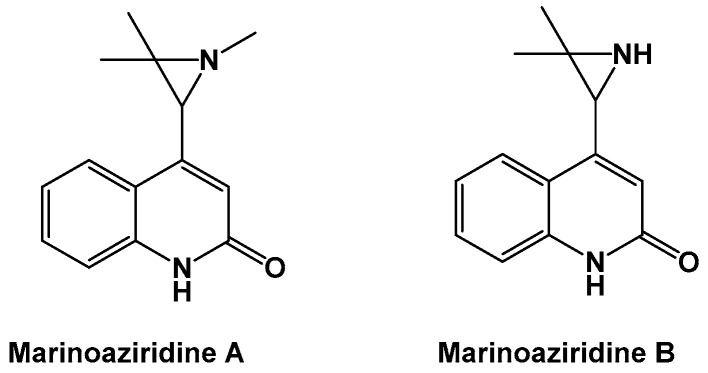
Chemical structures of marinoaziridines A and B.

As a result of marine bioprospecting, chiral alkaloids marinoaziridines A and B were recently isolated from marine sediment Gram-negative bacteria of the order Cytophagales (Figure 1) [1]. In the structure, they contain interesting aziridine and quinoline-2-(1*H*)-one ring, often present in bioactive natural and synthetic molecules [2,3,4].

**Scheme 1:**
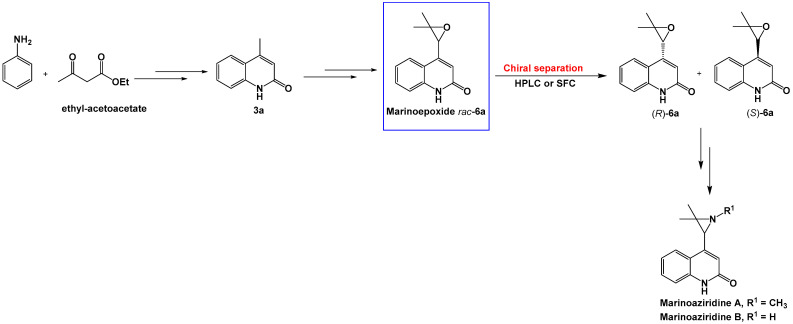
Proposed synthetic pathway towards marinoaziridines A and B.

The total synthesis of these compounds is not known yet, nor their absolute configurations. After evaluating several potential strategies, we developed a retrosynthetic analysis of marinoaziridines A and B. As shown in Scheme 1, our proposed synthetic pathway involves the preparation and enantioseparation of an epoxy synthetic intermediate, which we accordingly named marinoepoxide *rac*-**6a**. We envisaged access to marinoaziridines A and B via Staudinger reduction of the azido alcohol generated by ring opening of the marinoepoxide *rac*-**6a** [5,6].

**Figure 2:**
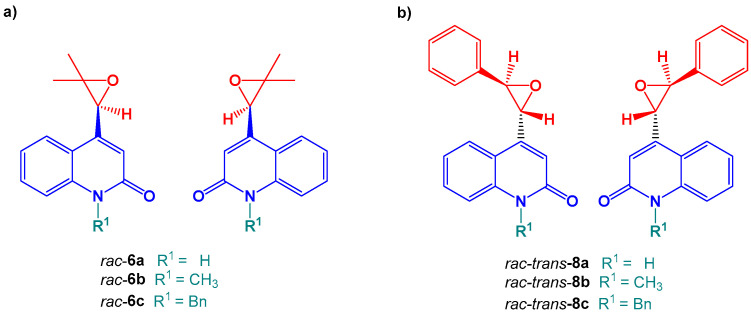
Chemical structures of newly synthesized marinoepoxides: (**a**) marinoepoxides *rac*-**6a**–**c**, (**b**) marinoepoxides *rac*-*trans*-**8a**–**c** (major diastereomer).

Along with the key synthon molecule *rac*-**6a** of marinoaziridines A and B, we also synthesized another group of its structural analogues *rac*-**6b**,**c** and *rac*-*trans*-**8a**–**c** (major diastereomer), since our preliminary in silico calculation results suggested interesting bioactive properties of this new class of organic epoxides as well as their aziridine counterparts (Figure 2).

With the goal of enantioseparation of racemic marinoepoxide *rac*-**6a** as the key intermediate in the total synthesis of marinoaziridines A and B, we applied chiral chromatography to access its pure enantiomers. For that purpose we used HPLC and SFC chromatography with dimethyl carbonate/alcohol and supercritical CO_2_/alcohol, respectively, as green solvent mobile phases along with two immobilized polysaccharides chiral columns (CHIRAL ART Amylose-SA, CHIRAL ART Cellulose-SC). Dimethyl carbonate (DMC; *T*_b_ = 90.3 °C, d = 1.069 g cm^−3^) is a non-polar aprotic solvent that mixes relatively well with organic solvents (alcohols, ethers, esters, ketones) and has partial solubility in water (139 g L^−1^) [7,8]. Its use in liquid chromatography in species analysis using HPLC-ICP-MS techniques is well known, but not in chiral chromatography. Due to its desirable properties, it is used as: solvent in organic reactions, reagent in chemical transformations and electrolyte in ion batteries [9]. DMC can be regarded as a true green solvent due to its nontoxicity and biodegradability [10,11,12,13]. Supercritical carbon dioxide is also considered to be an environmentally friendly solvent because it is non-toxic, inexpensive, inert, readily available, non-flammable, non-corrosive and mixes with a large number of organic solvents [14,15,16]. Its critical temperature and pressure are relatively low (*T*_c_ = 31 °C, *P*_c_ = 73.8 bar), and it possesses low viscosity, dielectric constant and surface tension [17]. Its poor UV absorption at low wavelengths (195, 205 and 210 nm, when mixed with acetonitrile, methanol and ethanol) makes this solvent compatible with UV and DAD detection [16]. SFC technique is increasingly used in the pharmaceutical industry for drug analysis, development and purification [18]. SFC is a particularly widespread technique for determining the enantiomeric purity of chiral compounds, but also for separating enantiomers on a preparative scale [19,20,21,22,23]. The exigency of preparative scale SFC protocols for enantiopure compounds is rising from its comparative advantages compared to HPLC such that the following are present: (a) faster flow-rate and column equilibration due to lower viscosity; (b) higher column loadability, hence productivity, is better than HPLC; (c) solubility in supercritical fluid decreases rapidly as the pressure decreases near the critical point, facilitating recovery of the isomer fractions and increasing yield; (d) instrumentation allows stacked or overlapped injections improving productivity and reducing solvent consumption; (e) mobile phase is mainly constituted of carbon dioxide, which is non-flammable, less expensive and less toxic than common organic solvents. An increased need for development of efficient chiral separation methods is based on the fact that the vast majority of compounds that carry biological activity are chiral molecules [24].

In this work, we also studied the enantioseparation of (±)-2,3,3-trisubstituted marinoepoxides *rac*-**6b**,**c** and (±)-*trans*-2,3-disubstituted marinoepoxides *rac***-8a**–**c** (major diastereomer) under the same chromatographic conditions as for *rac*-**6a**. Analyzed marinoepoxides possess one (*rac*-**6a**–**c**) or two centers of chirality *(rac*-**8a**–**c**), thus allowing existence of two or four stereoisomers, respectively. In the case of marinoepoxides *rac*-**8a**–**c**, only *trans* diastereoisomer was analyzed, resulting in two separated enantiomers.

To our knowledge, along with our recent paper [23], this is the only example of the efficient use of dimethyl carbonate as a green solvent in chiral chromatography.

## 2. Results and Discussion

### 2.1. Synthesis of Racemic Marinoepoxides rac-***6a**–**c*** and rac-***8a**–**c***

The Johnson–Corey–Chaykovsky reaction was applied for the synthesis of both trisubstituted and disubstituted marinoepoxides from quinoline-2(1*H*)-4-carbaldehydes **4a**–**c**. The formation of a new C-C bond and the formation of a three-membered heterocyclic ring was achieved by the reaction between sulfonium salt **5** or **7** base-generated in situ sulfur ylide and a carbonyl compound, specifically quinoline-2(1*H*)-one-4-carbaldehyde **4a**–**c**. The structure and purity of isolated marinoepoxides were confirmed by ^1^H and ^13^C NMR spectroscopy, high-performance liquid chromatography (HPLC) and high-resolution mass spectroscopy (HRMS).

#### 2.1.1. Synthesis of Trisubstituted Racemic Marinoepoxides *rac*-**6a**–**c**

Marinoepoxides *rac*-**6a**–**c** possess the same core structure of quinoline-2-(1*H*)-one fragment. Therefore, we envisioned building the aldehydes **4a**–**c** first.

**Scheme 2:**
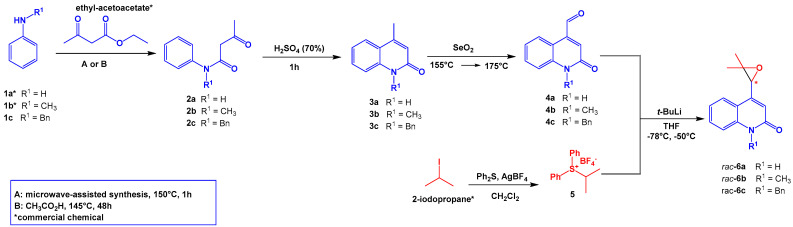
Synthesis of trisubstituted marinoepoxides *rac*-**6a**–**c**.

Trisubstituted quinoline-2(1*H*)-one epoxides *rac*-**6a**–**c** were prepared through the convergent synthetic route using isopropyl(diphenyl)sulfonium tetrafluoroborate **5** as a source of sulfur ylide, and the corresponding aldehydes **4a**–**c** (Scheme 2) as the key synthetic step. Commercially available aniline, *N*-methylaniline and ethyl-acetoacetate were adopted as the starting materials, aldehydes **4a**–**c** were obtained via condensation and cyclization process at Knorr condition, and Riley oxidation was obtained in good yield over three steps. The other fragment, achiral sulfonium salt **5**, was prepared from the commercially available benzyl chloride and dimethyl sulfide in the presence of the AgBF_4_ as a promoter in good yield.

Since in this case it was necessary to add two methyl groups to the carbonyl carbon atom, i.e., to cleave the proton from the tertiary carbon, we had to take into account the acidity of the proton being cleaved. Isopropyl(diphenyl)sulfonium triflate **5** contains two methyl groups attached to a carbon atom in the α position of the sulfur atom, and the cleavage of the carbon-hydrogen bond produces the desired ylide. The *tert*-butyl-lithium base, with a pK_a_ value of about 50, proved to be an extremely good choice regarding the proton acidity. Marinoepoxides *rac*-**6a**–**c** were isolated by extraction with several solvent types and further by silica-gel column chromatography, giving yields of 14.6%, 35% and 86%, respectively.

#### 2.1.2. Synthesis of Disubstituted Racemic Marinoepoxides *rac*-**8a**–**c**

**Scheme 3:**
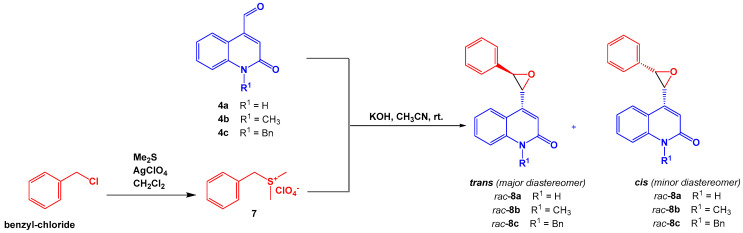
Synthesis of 2,3-disubstituted marinoepoxides *rac*-**8a**–**c**.

Disubstituted quinoline-2(1*H*)-one marinoepoxides *rac*-**8a**–**c** were prepared through the convergent synthetic route using benzyl(dimethyl)sulfonium perchlorate **7** as a ylide source, and the corresponding aldehydes **4a**–**c** as a mixture of diastereoisomers (Scheme 3). Achiral sulfonium salt **7** was prepared from the commercially available 2-iodopropane and diphenyl sulfide in the presence of the AgClO_4_ as a promoter in good yield.

The stereochemistry of marinoepoxides *rac*-**8a**–**c** was determined by comparison of the coupling constants between the protons in the epoxide ring; for *cis-* isomers 4.3 to 7.2 Hz and for *trans-*isomers 1.9 to 2.2 Hz, respectively. According to the ^1^H-NMR, the *trans-* isomer predominates. *Cis-* and *trans-* isomers of (±)-2,3-disubstituted marinoepoxides *rac*-**8a**,**c** have not been successfully separated by achiral column chromatography on silica gel, therefore they were used in chiral analysis as diastereomeric mixtures (Table 1).

### 2.2. Enantioselective Chromatography of (±)-Marinoepoxides rac-***6a**–**c*** and rac-***8a**–**c***

In this study, chromatographic conditions for the separation of marinoepoxides enantiomers *rac*-**6a**–**c** and *rac***-8a**–**c** on immobilized chiral stationary phases were investigated using high-performance liquid chromatography (HPLC) and supercritical fluid chromatography (SFC). Three chiral columns, CHIRAL ART Cellulose-SC, CHIRAL ART Cellulose-SB and CHIRAL ART Amylose-SA with immobilized chiral selector were examined (Table 2). In our preliminary studies, the CHIRAL ART Cellulose-SB column did not prove to be a good choice in the separation of marinoepoxides enantiomers, so the optimization of chromatographic conditions was performed on the remaining two immobilized stationary phases. The developed enantioselective analytical methods will be used in control of enantiomeric purity during stereoselective synthesis of target molecules, and also on a preparative scale chiral chromatography. Chiral recognition using polysaccharide-based chiral stationary phases is based on a complex process involving chiral and achiral interactions between the analyte, the chiral selector and the mobile phase. Enantiorecognition of trisubstituted *rac*-**6a**–**c** and disubstituted marinoepoxides *rac*-**8a**–**c** is based on attractive interactions such as π-π interactions, dipole-dipole interactions and hydrogen bonds, and repulsive interactions that are usually steric. Polar and π-π interactions between phenyl groups of the chiral stationary phase and the analyte play an important role in the chiral recognition mechanism [24,25,26] Based on numerous studies, it is difficult to predict which chiral selector of a particular stationary phase will separate the enantiomers of a compound, and which mobile phase will be the most favorable. The development of a good enantioselective chromatographic method requires finding the best combination of process parameters, described by chiral stationary phase selection, mobile phase composition and concentration, column temperature and back pressure (mainly for SFC) [27].

All investigated marinoepoxides *rac*-**6a**–**c**, and *rac*-**8a**–**c** have different substitutions (steric effects) on the nitrogen atom of the internal amide bond of quinoline-2(1*H*)-one moiety, and therefore possess different spatial orientation, due to which they possess different affinities towards the chiral stationary phase selector. In the case of disubstituted marinoepoxides *rac*-**8a**–**c**, it was important to determine whether the bulkiness of the phenyl ring on the C3 carbon atom of the epoxy ring (inability to adhere to the chiral cavity) or π-π interactions with the stationary phase selector is decisive for chiral recognition. Marinoepoxides *rac*-**6a**–**c** contain two methyl groups on the C3 atom instead of the phenyl ring, and the effect of less voluminous groups on the chiral recognition mechanism was investigated. The enantioseparation tests were firstly performed using HPLC with *n*-hexane/ethanol (80/20, *v*/*v*) as a standard, widely used as a solvent system to compare with green alternatives. Further enantioseparation tests were performed using green solvent systems as pure dimethyl carbonate and dimethyl carbonate/alcohol (*v*/*v*) mixtures. Particular attention was paid to the use of SFC with CO_2_/alcohol (*v*/*v*) as nonpolar mobile phase. In the *n*-hexane binary system, only ethanol was used as the alcohol modifier. Using propan-2-ol, a long retention time of enantiomers (t_R_ > 110 min) was observed, while methanol was not used in this study due to poor miscibility with *n*-hexane. The effect of the addition of an alcohol modifier (methanol or ethanol) on the enantioselectivity and retention times was investigated also in the dimethyl carbonate binary system as well as in the SFC system. It has been hypothesized that increasing the polarity of the mobile phase leads to less pronounced hydrogen bonds between the chiral stationary phase and the analyte. Moreover, alcohol molecules combine with the chiral stationary phase and cause swelling of the column, which directly affects the size and shape of the chiral cavity. If chiral cavities are opened, the inclusion interactions of the analyte are reduced and the retention time of the analyte is reduced [24,28,29,30]. Pirkle and Welch studied the effect of an alcohol modifier on enantioselectivity and found that the effect of a modifier depends on the structure of the analyte [31,32,33]. Based on VCD measurements Grinberg et al. observed conformational changes of the chiral stationary phase in the presence of polar solvents. Conformations changed drastically depending on the concentration and effect of the shape and size of chiral cavities, i.e., the mechanism of chiral recognition [34].

For each enantiomeric pair analyzed, the values of retention factors *k*_1_ and *k*_2_, separation factor (separation factor) α and resolution of *R*_s_ enantiomers obtained at each chiral stationary phase with a certain mobile phase composition are given. The retention factors *k*_1_ and *k*_2_ of individual enantiomers were calculated according to the equations:

*k*_1_ = (t_R1_ − t_0_)/t_0_

*k*_2_ = (t_R2_ − t_0_)/t_0_

In the HPLC mode, the retention time of the mobile phase (t_0_) was determined as the retention time of 1,3,5-tri-*tert*-butylbenzene, a compound that passes through the column without any non-covalent interaction with the chiral stationary phase.

In the SFC mode, the solvent front was taken as the mobile phase retention time (t_0_). The separation factor α was calculated according to the equation:

*α* = *k*_2_/*k*_1_

The resolution factor *R*_s_ was calculated according to equation:

*R*_s_ = 1.18 × (t_R2_ − t_R1_)/(w_b_ + w_h_)

where *w*_b_ denotes the width of the chromatographic peak, and *w*_h_ the width of the peak at half the peak height.

#### 2.2.1. HPLC and SFC Enantioselective Chromatography of (±)-Trisubstituted Marinoepoxides *rac*-**6a**–**c**

**Figure 3:**
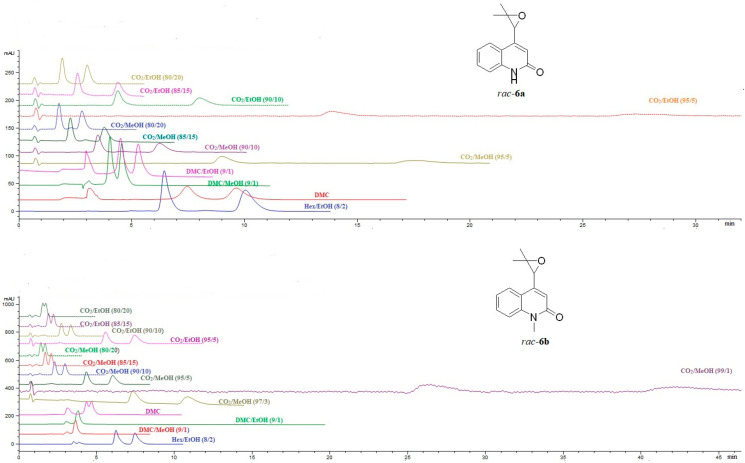

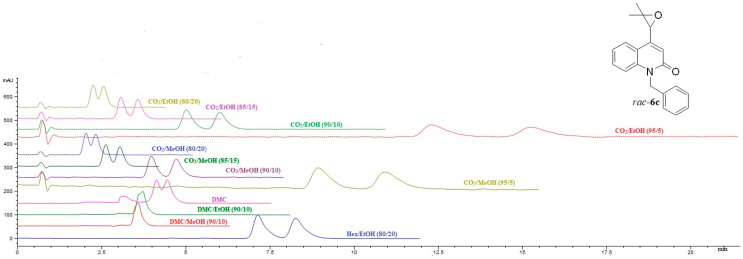
HPLC and SFC enantioseparation of racemic marinoepoxides *rac*-**6a**–**c** on CHIRAL ART Amylose-SA column.

**Figure 4:**
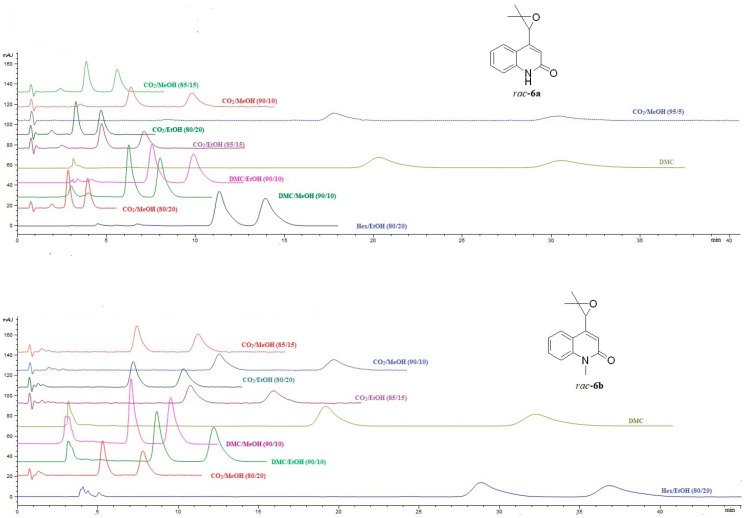

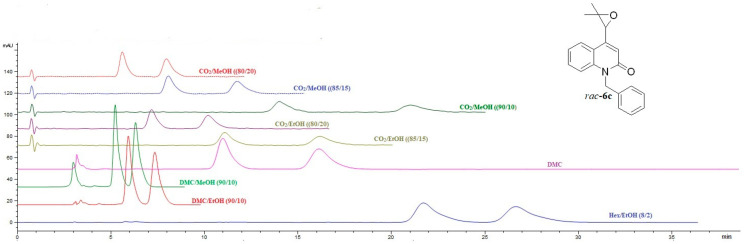
HPLC and SFC enantioseparation of racemic marinoepoxides *rac*-**6a**–**c** on CHIRAL ART Cellulose-SC column.

The results of HPLC enantioselective analysis of marinoepoxides *rac*-**6a**–**c** are shown in Table S1 (Supplementary materials) (Figure 3 and Figure 4). It was observed that enantioseparation of marinoepoxides *rac*-**6a**,**b** shows a higher degree of enantioselectivity on the CHIRAL ART Amylose-SA column, while compound *rac*-**6c** achieves better enantioselectivity on the CHIRAL ART Cellulose-SC column when using hexane/EtOH (80/20, *v*/*v*) as a mobile phase. By replacing hexane/EtOH (80/20, *v*/*v*) mobile phase with dimethyl carbonate, all enantiomers of marinoepoxides *rac*-**6a**–**c** showed enantioseparation on the CHIRAL ART Cellulose-SC column in comparison to CHIRAL ART Amylose-SA column.

From the obtained results, excellent baseline enantioseparation of all marinoepoxides *rac*-**6a**–**c** on the selected CHIRAL ART Cellulose-SC column in the HPLC mode was observed. Comparing the separation factors for enantiomers of all three compounds *rac*-**6a**–**c** with *n*-hexane/ethanol (80/20, *v*/*v*), it can be observed that the degree of enantioselectivity is almost independent of the substituent (-H, -CH_3_, -Bn) on the nitrogen atom of the internal amide bond of quinoline-2(1*H*)-one moiety. This finding indicates that the mechanism of chiral recognition is not dominated by steric interactions. The chiral stationary phase selector in CHIRAL ART Cellulose-SC contains two electron-withdrawing groups that reduce the electron density of the benzene system and increase the hydrogen acidity of the phenylcarbamate group [35]. Given these facts, it can be assumed that chiral recognition of marinoepoxide enantiomers *rac*-**6a**–**c** with selector *tris*-(3,5-dichlorophenylcarbamoyl)cellulose is based on hydrogen bonds and dipole-dipole interactions. By comparing the retention factors, it can be observed that the enantiomers of compound *rac*-**6b** show the longest retention time on the tested cellulose column. The longer retention of the enantiomers of compound *rac*-**6b** on the column relative to the remaining two compounds *rac*-**6a** and *rac*-**6c** may be due to strong dipole-dipole interactions or hydrogen bonds that can further stabilize the diastereomeric complex [36]. The electron-donating methyl group on the nitrogen atom in the analyte structure makes the C=O group a better acceptor of the hydrogen bond that can be achieved with the hydrogen atom in the phenylcarbamate group of the chiral selector. Longer retention times for enantiomers of *rac*-**6b** (*R*_s_ = 2.67) led to higher values of the resolution factor compared to the enantiomers of compounds *rac*-**6a** (*R*_s_ = 2.12) and *rac*-**6c** (*R*_s_ = 2.13).

The use of dimethyl carbonate as a mobile phase reduces the retention time of the enantiomers and increases the resolution factor for all marinoepoxides *rac*-**6a**–**c** in the sequence: *k*_1_ (20.34; *rac*-**6a**) > *k*_1_ (19.15; *rac*-**6b**) > *k*_1_ (10.96; *rac*-**6c**), or *R*_s_ (4.70; *rac*-**6b**) > *R*_s_ (3.31; *rac*-**6a**) > *R*_s_ (3.19; *rac*-**6c**). Shorter retention of enantiomers and higher values of *R*_s_ resolution factors for compounds *rac*-**6a**–**c** may be the result of dipole-dipole interactions of the solvent and stationary phase, with these interactions contributing to more significant enantiorecognition of chiral phase and analytes. This result confirms the thesis that the contribution of π-π interactions in the process of chiral discrimination is very small. The addition of methanol or ethanol with a volume fraction of 10% to the dimethyl carbonate mobile phase increases the polarity of the mobile phase and reduces the retention factor of the analyte on the stationary phase in the range *k*_H_ > *k*_CH3_ > *k*_Bn_. This indicates a strong competition of alcohols and analytes for interaction sites on the chiral stationary phase. At a volume fraction of 10% methanol or ethanol, α values and resolution factor decrease.

If we compare the enantioselectivity for enantiomers of compounds *rac*-**6a**–**c** on cellulose and amylose columns using SFC chromatography, then the enantiomers of compound *rac*-**6a** showed better enantioselectivity on the CHIRAL ART Amylose-SA column. The different chiral recognition mechanism is possibly due to the formation of different shapes and sizes of chiral cavities that build carbamate groups on adjacent glucose units of amylose and cellulose depending on the mobile phase [37].

The application of supercritical fluid chromatography in the enantioseparation of marinoepoxides *rac*-**6a**–**c** on a selected cellulose column showed excellent chiral recognition with a stationary phase selector, regardless of the type and volume fraction of alcohol modifier in the CO_2_ binary system. All enantiomers of compounds *rac*-**6a**–**c** were separated to the baseline, regardless of the composition of the mobile phase. By comparing the retention factors of the starting enantiomers *rac*-**6a**–**c** on the cellulose column, it was observed that the enantiomers remain longer with ethanol as an alcohol modifier in the series *k*_H_ > *k*_CH3_ > *k*_Bn_. The separation factors of the enantiomers of compounds *rac*-**6a**–**c** are almost constant by changing the type and volume fraction of the alcohol modifier. Compound *rac*-**6a** (α = 1.62) showed the best enantioselectivity with CO_2_/EtOH (80/20, *v*/*v*) mobile phase, while compounds *rac*-**6b** (α = 1.58) and *rac*-**6c** (α = 1.51) showed the best enantioselectivity using CO_2_/MeOH (80/20, *v*/*v*) as mobile phase. The values of the separation factors differ slightly by changing the alcohol modifier. By comparing the separation factors in the HPLC and SFC mode, it was observed that compounds *rac*-**6b** and *rac*-**6c** show better enantioselectivity with dimethyl carbonate compared to the enantioselectivity obtained using CO_2_/alcohol (*v*/*v*) as binary mobile phase. Compound *rac*-**6a** showed better enantioselectivity using supercritical fluid chromatography, regardless of the composition and volume of the alcohol in the CO_2_ mobile phase. By comparing the resolution factors of the enantiomers of compounds *rac*-**6a**–**c**, excellent resolution (*R*_s_ > 2.06) was observed in the entire test range from 20% to 5% by volume of alcohol in the CO_2_ binary system. The presented results indicate that the reduction of the alcohol content by volume in the CO_2_ binary system leads to longer retention of the enantiomer on the column and better resolution, i.e., better enantiorecognition of the analyte with the stationary phase selector.

The main advantage of developed SFC enantioselective methods is particularly visible during HPLC and SFC enantioseparation of the key intermediate molecule and its derivatives, racemic marinoepoxides *rac*-**6a**–**c**, on CHIRAL ART Amylose-SA and CHIRAL ART Cellulose-SC columns. In all cases, SFC chiral chromatography proved to be the method of choice in terms of lower retention times and higher separation factors. This is especially true when using CHIRAL ART Amylose-SA for enantioseparation of *rac*-**6b**. In this case the only green chromatography method is achieved using SFC, while using HPLC with green solvent (DMC) revealed only partial enantioseparation. For comparison, the HPLC method using *n*-hexane/EtOH mixture revealed longer retention times and lower enantioselectivity for *rac*-**6b**.

Figure 4 shows the enantioseparation chromatograms of marinoepoxides *rac*-**6a**–**c** separated on a CHIRAL ART Cellulose-SC column, with different mobile phase compositions, both in HPLC and SFC mode.

The amylose-based chiral stationary phase CHIRAL ART Amylose-SA contains two electron-donating methyl groups on the phenyl ring that increase the electron density of the benzene ring and the C=O group and reduce the hydrogen acidity of the phenylcarbamate group of the chiral selector. Given the described electronic properties of the amylose-based chiral stationary phase, enantiorecognition of trisubstituted marinoepoxides *rac*-**6a**–**c** is based on the formation of π-π interactions and dipole-dipole interactions, and repulsive interactions that are mostly steric. In the case of compound *rac*-**6a**, both the hydrogen bond via the C=O group of the chiral selector and the hydrogen atom of the internal amide bond of marinoepoxide structure can be achieved.

Using SFC chromatography in enantioseparation of marinoepoxides *rac*-**6a**–**c**, revealed an excellent baseline separation of *rac*-**6a**. The enantiomers of compound *rac*-**6a** are retained shorter with better resolution in the SFC mode compared to the HPLC mode. Moreover, the enantiomers of compound *rac*-**6b** showed excellent baseline separation using alcohol modifier ranging from 3% to 10% (methanol or ethanol, *v*/*v*). By comparing the retention factors of enantiomers *rac*-**6a**–**c** on the amylose-based column, it can be observed that the enantiomers retain longer with ethanol as modifier in the sequence *k*_Bn_ > *k*_H_ < *k*_CH3_. The separation factors for enantiomers of *rac*-**6a**–**c** are almost constant by changing the type and volume fraction of the alcohol modifier. The highest values of the separation factors for compounds *rac*-**6a**–**c** were achieved for compound *rac*-**6a** (α = 2.12) with CO_2_/EtOH (80/20, *v*/*v*), compound *rac*-**6b** (α = 1.55) with CO_2_ / MeOH (97/3, *v*/*v*) and compound *rac*-**6c** (α = 1.25) with CO_2_/EtOH (95/5, *v*/*v*). The highest values of the resolution factor *R*_s_ in the SFC mode were achieved for compound *rac*-**6a** (*R*_s_ = 4.83) with CO_2_/EtOH (95/5, *v*/*v*), compound *rac*-**6b** (*R*_s_ = 3.01) with CO_2_/MeOH (97/3, *v*/*v*) and compound *rac***-6c** (*R*_s_ = 2.04) with CO_2_/EtOH (95/5, *v*/*v*). The presented results indicate that the reduction of the alcohol content by volume in the CO_2_ binary system leads to longer retention of the enantiomers on the column and better resolution, i.e., better enantiorecognition of the analyte with the chiral stationary phase selector.

#### 2.2.2. HPLC and SFC Enantioselective Chromatography of (±)-Disubstituted Marinoepoxides *rac*-**8a**–**c**

**Figure 5:**
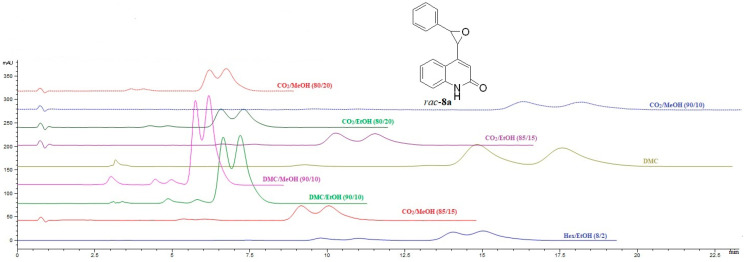

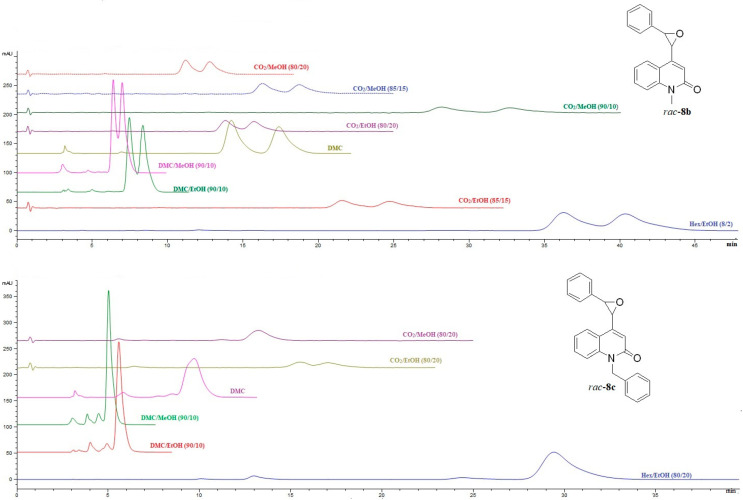
HPLC and SFC enantioseparation of racemic marinoepoxides *rac*-**8a**–**c** on CHIRAL ART Cellulose-SC column.

**Figure 6:**
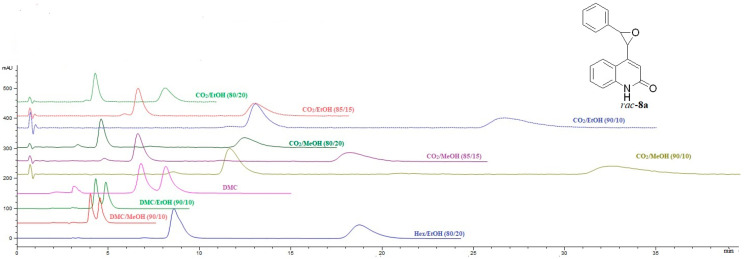

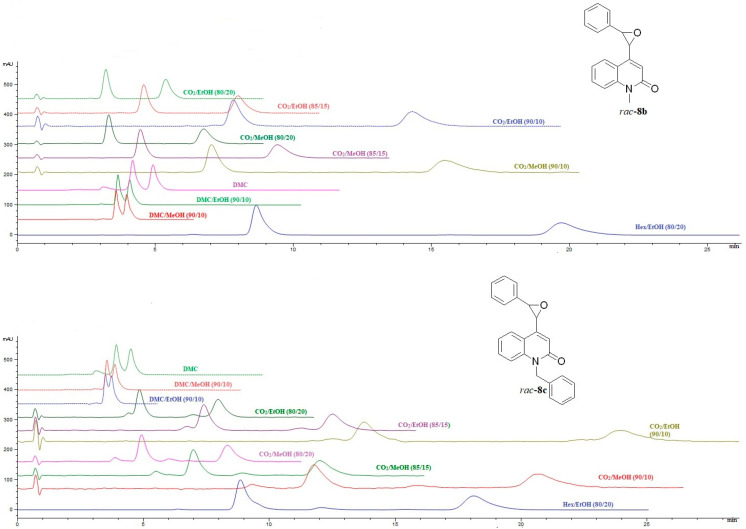
HPLC and SFC enantioseparation of racemic marinoepoxides *rac*-**8a**–**c** on CHIRAL ART Amylose-SA column.

The results of enantioseparation of marinoepoxides *rac*-**8a**–**c** on cellulose-based chiral stationary phase CHIRAL ART Cellulose-SC, and amylose-based chiral stationary phase CHIRAL ART Amylose-SA are shown in Table S2 (Supplementary materials). It is interesting to note that the analytes *rac*-**8a**–**c** showed a higher degree of enantioselectivity on the CHIRAL ART Amylose-SA column than on the CHIRAL ART Cellulose-SC column, regardless of the mobile phase composition (Figure 5 and Figure 6).

It is known from the literature that the most common interactions of the chiral selector present in the CHIRAL ART Cellulose-SC and analyte are dipole-dipole interactions and hydrogen bonds [35]. In the case of marinoepoxides *rac*-**8a**–**c**, dipole-dipole interactions can occur via polar C=O---C=O and C-Cl---C=O group of the chiral selector and analyte, respectively. Due to the possibility of involving at least four poles in these interactions, it has been suggested that such interactions are primarily dipolar [38,39]. If dipole formation does not occur due to the unfavorable position of the two groups, intimate interactions can be considered, i.e., short contact or n→π* interactions [40]. Non-covalent intermolecular hydrogen bonding can be achieved by interaction via carbamate group of chiral selector with C=O group or with oxygen in the epoxy ring of marinoepoxides. In the case of compound *rac*-**8a**, there are two additional possibilities with regard to the literature that quinoline-2(1*H*)-ones can occur in two tautomeric forms [41]. If the molecule is in the enol form, then the hydrogen bond can be realized through the OH group in the analyte structure with the C=O group of the chiral selector. By applying *n*-hexane/ethanol (80/20, *v*/*v*) mobile phase, only partial separation of the enantiomers of compounds *rac*-**8a**,**b** was achieved, with long retention of the enantiomers of *rac*-**8b** (*k*_1_ = 36.26, *k*_2_ = 40.36) on the tested cellulose columns. By comparing the resolution factors of compounds *rac*-**8a** and *rac*-**8b**, it was observed that their *R*_s_ values follow the sequence: *R*_s_ (*rac*-**8b**) > *R*_s_ (*rac-***8a**). Such results contribute to the thesis that the dipole-dipole interaction and hydrogen bond dominate in the chiral recognition mechanism. Given the structural difference between the compounds *rac*-**8a**–**c**, it can be assumed that the dominant interactions affecting the chiral recognition mechanism are realized through the C=O group in the analyte structure. Since compound *rac*-**8b** showed the best enantioselectivity, regardless of the composition of the mobile phase in the HPLC mode, it can be concluded that the electron-donating methyl group has a great influence, which increases the electron density of the internal amide bond and thus contributes to stronger interactions.

Using dimethyl carbonate as the mobile phase, the best results of enantiomer separation were achieved, and the enantiomers of the two compounds *rac*-**8a** (α = 1.23; *R*_s_ = 1.49) and *rac*-**8b** (α = 1.28; *R*_s_ = 1.85) showed similar enantioselectivity. The addition of 10% methanol or 10% ethanol to the mobile phase reduces the degree of chiral recognition with the stationary phase selector in CHIRAL ART Cellulose-SC. It should also be noted that the retention times of the analyte on the column, which can be seen by observing the *k*_1_ and *k*_2_ data, are much shorter compared to the retention times of the enantiomer with dimethyl carbonate as the mobile phase. The planar carbonyl group of the marinoepoxides *rac*-**8a**–**c** has a relatively large dipole moment and therefore has a high solvating power. Polar molecules of methanol or ethanol surround the carbonyl group of the analyte and it becomes sterically disturbed, which may affect the weaker interactions with the chiral stationary phase selector.

The use of ethanol (*v*/*v*) in enantioseparation of *rac*-**8a** achieved better chiral recognition and slightly longer retention times of the enantiomers (*R*_s_ = 1.24; *k*_1_ = 2.10; *k*_2_ = 2.49) than in comparison with methanol (*R*_s_ = 0.69; *k*_1_ = 0.93; *k*_2_ = 1.08). The absorbency of the analyte on the chiral stationary phase, together with the retention factor, is increased by decreasing the volume fraction of the alcohol modifier. By reducing the polarity of the mobile phase, the interaction between the enantiomers of the analyte and the stationary phase is significantly increased, which causes longer retention times of enantiomers and their better separation [42]. Longer retention times can consequently lead to circular and longitudinal diffusion [28].

Better chiral recognition of compounds *rac*-**8a** and *rac*-**8b** with the chiral selector *tris*-(3,5-dichlorophenylcarbamoyl)cellulose was not achieved by the use of SFC, regardless of the type and volume of alcohol modifier in the CO_2_ binary system. According to the results obtained, it was observed that the best enantioselectivity was shown by compound *rac*-**8b** having a methyl group attached to the internal amide bond of quinoline-2(1*H*)-one moiety. By comparing the retention factors of the first-emerging enantiomers of compounds *rac*-**8a**–**c** on the selected cellulose-based chiral stationary phase, the longest retention for enantiomers of *rac*-**8c** having a benzyl group attached to the internal amide bond within the quinoline-2(1*H*)-one structure was observed. By examining the effect of the addition of an alcohol modifier in the CO_2_ mobile phase, it was found that compounds *rac*-**8a**-**b** showed better enantioselectivity by the addition of ethanol compared to more polar methanol. Reducing the volume fraction of ethanol in the CO_2_ binary system leads to lower enantioselectivity for *rac*-**8a**-**b**. It was also observed that reducing the volume fraction of alcohol in the CO_2_ binary system leads to longer retention times of the analyte, and at the same time to higher values of the resolution factor *R*_s_ [28]. Enantioseparation of *rac*-**8c** with CO_2_/EtOH (85/15, *v*/*v*) showed slightly better enantioselectivity with a resolution factor *R*_s_ = 1.20 and a retention factor *k*_1_ = 25.26 of the first enantiomer, relative to CO_2_/EtOH (80/20, *v*/*v*) as the mobile phase. It is interesting to note that with CO_2_/MeOH (80/20, *v*/*v*) there is a complete lack of enantioseparation. A change in the polarity and volume of alcohol is sufficient to change the geometry and/or size of chiral cavities, which may affect the chiral recognition mechanism [26,43,44,45]. Polar alcohols will form stronger hydrogen bonds with the chiral stationary phase, due to the fact that they can diffuse more easily into a well-defined chiral cavity of the stationary phase [33]. Therefore, less stable complexes will be formed, which will then lead to lower *R*s and α. Consistent with the results obtained in the HPLC mode, it was observed that the enantiomers of the analyte were retained shorter with methanol in the CO_2_ binary system than with ethanol.

Comparing the results of the enantioselective analysis, it was observed that the enantiomers of compound *rac*-**8b** with a methyl group attached to the internal amide bond of quinoline-2(1*H*)-one showed the best chiral recognition with chiral selector *tris*-(3,5-dichlorophenylcarbamoyl)cellulose, both in HPLC and SFC mode. In the HPLC mode, the enantiomers of compound *rac*-**8b** are retained the longest with the best enantioselectivity, while the longest retention time in the SFC mode was observed for the enantiomers of compound *rac*-**8c** with the lower enantioselectivity. Comparing the results of both modes, it was observed that compound *rac*-**8b** showed the best enantioseparation results (α = 1.28, *R*_s_ = 1.85) with dimethyl carbonate as the mobile phase.

When the type of chiral stationary phase in the enantioselective system changes from the CHIRAL ART Cellulose-SC to the CHIRAL ART Amylose-SA, the chiral recognition mechanism also changes. In amylose, the geometry of the α-1,4-glycosidic bond between D-(+)-glucose units causes the natural bending of the associated glucopyranoses into a hollow helix. With such spatial organization, glucose units are properly distributed and make well-defined chiral cavities [46]. A potentially stronger hydrogen bond can only be achieved in the case of compound *rac*-**8a** via the carbamate group of the chiral selector and hydrogen of the amide group in the analyte structure when it is in the keto form. If compound *rac*-**8a** is in the enol form, it is possible to form a hydrogen bond via the carbamate group of the chiral selector and the hydrogen atom of the hydroxyl group in the analyte structure. In addition to affecting the hydrogen acidity of the phenylcarbamate group, methyl groups also have a role in controlling the entry of enantiomers into the chiral cavity of the amylose chiral stationary phase [47].

Based on the obtained results, excellent enantiorecognition of compounds *rac*-**8a**–**c** with *tris*-(3,5-dimethylphenylcarbamoyl)amylose chiral selector (CHIRAL ART Amylose SA column) can be observed using both HPLC and SFC. All marinoepoxide structures *rac*-**8a**–**c** showed efficient chiral recognition and shorter enantiomer retention times, regardless of the mobile phase composition in HPLC and SFC mode compared to CHIRAL ART Cellulose-SC. The results of enantioseparation of compounds *rac*-**8a**–**c** on amylose-based chiral stationary phase indicate that in the mechanism of chiral recognition, in addition to noncovalent dipole-dipole and π-π interactions, the placement of diastereomeric complex in the chiral cavity of the selector has a significant contribution. As the tertiary structure of amylose is different from cellulose, it is to be expected that the mechanism of chiral recognition will be different. The higher structure of the amylose coil in this case favors the entry of the less sterically demanding enantiomer [43]. Using dimethyl carbonate as the mobile phase, worse enantiomer separation results are obtained for marinoepoxides *rac*-**8a**–**c** on the amylose-based chiral stationary phase. The highest observed value of the separation factor was α = 1.63 for compound *rac*-**8c**, while compound *rac*-**8c** showed slightly less enantioselectivity with α = 1.61. Examination of the influence of different volumes of alcohol in dimethyl carbonate mobile phase on the separation of enantiomers of compound *rac*-**8a**–**c** showed the dependence of chiral recognition on the percentage and type of alcohol modifier in the mobile phase. The addition of 10% methanol and 10% ethanol, respectively, reduces the retention factor and the resolution factor of compounds *rac*-**8b**–**c**. The values of the separation factors for compounds *rac*-**8a** and *rac*-**8b** are higher with the addition of the alcohol modifier to the dimethyl carbonate mobile phase. Compound *rac*-**8a** showed slightly better enantioselectivity by the addition of methanol, with a longer retention time of the enantiomers than with ethanol. On the other hand, the enantiomers of compounds *rac*-**8b** and *rac*-**8c** showed slightly better enantiorecognition with the addition of ethanol than with the addition of methanol. The obtained results indicate that the chiral recognition of the enantiomers of compounds *rac*-**8a**–**c** with the selector of the tested amylose-based stationary phase depends on the substituents on the nitrogen atom of the internal amide bond, the mobile phase, but also on the supramolecular structure of the stationary phase.

Better enantioselectivity was achieved in the SFC mode compared to the HPLC mode for the selected amylose-based chiral stationary phase. As shown in Table S2 (Supplementary materials), all marinoepoxides *rac*-**8a**–**c** were efficiently baseline separated with high selectivity and resolution and shorter retention times on the amylose-based column with CO_2_ binary mobile phase. If we compare the enantiorecognition of compounds *rac*-**8a**–**c**, then it can be concluded that the best chiral recognition using *tris*-(3,5-dimethylphenylcarbamoyl) amylose selector was achieved with compound *rac*-**8a** having a free, non-substituted amide bond in quinoline-2(1*H*)-one moiety, regardless of composition and volume fraction of alcohol modifier in the CO_2_ binary system. While the lower enantiorecognition was achieved with a sterically more demanding compound *rac*-**8c** having a benzyl group attached to the nitrogen of the internal amide bond. These results indicated that the change in the shape of the chiral cavity is less conducive to the sterically more demanding compound *rac*-**8c**. By comparing the retention factors of the first eluted enantiomers of compounds *rac*-**8a**–**c** on the selected amylose stationary phase, the longest retention of enantiomers of compound *rac*-**8a** with unprotected internal amide bond is observed. By examining the effect of the addition of an alcohol modifier on the CO_2_ mobile phase, it was found that all compounds *rac*-**8a**–**c** showed better enantioselectivity by the addition of methanol compared to less polar ethanol. An enantioselective chromatographic system with methanol as an alcohol modifier was more efficient for separating the enantiomers of all three compounds. By comparing separation factors and resolution factors with CO_2_/MeOH (80/20, *v*/*v*) as the mobile phase, compound *rac*-**8a** showed the best enantioselectivity (α = 3.11) with the highest resolution factor (*R*_s_ = 6.33). Binding of the methyl and benzyl groups to the nitrogen atom of the internal amide bond of compounds *rac*-**8b**,**c** decreases the enantioselectivity. The sterically more demanding compound *rac*-**8c** showed the lower enantioselectivity within this group. It has also been observed that a decrease in the alcohol ratio in the binary system caused a longer retention of the enantiomers of *rac*-**8a**–**c** on the column, with an increase in the resolution factor. The best selectivity and resolution were achieved with CO_2_/ MeOH (90/10, *v*/*v*) for all marinoepoxides in this group. The bulky and planar benzyl substituent in compound *rac*-**8c** is sterically less suitable for the existing shape or size of the chiral cavity, while the less sterically demanding compound *rac*-**8a** is more suitable for placement in selector grooves. It should be also noted that compound *rac*-**8a** can achieve intensely strong hydrogen bonding via the NH group of the internal amide bond with the C=O group in the phenylcarbamate unit of the chiral selector, which may contribute to additional stabilization of the resulting diastereomeric complex or better chiral recognition.

## 3. Materials and Methods

### 3.1. Synthesis

All reactions were conducted under an argon atmosphere unless otherwise noted. All the solvents were either *puriss p.a.* quality or distilled over appropriate drying reagents. THF was distilled from sodium/benzophenone ketyl and CH_2_Cl_2_ was distilled from CaH_2_. All other reagents were purchased from commercial sources and used without purification. For the chromatographic separations, silica gel (Honeywell Fluka Analytical, 35–75 μm, Seelze, Germany) was used. Analytical thin-layer chromatography was performed on silica gel plates (DC-Alufolien-Kieselgel F_254_, Sigma Aldrich, Merck KGaA, Darmstadt, Germany). Visualization was carried out under ultra-violet irradiation (254 nm). NMR spectra were recorded on Bruker AV 600 MHz and 300 MHz spectrometers (Bruker Technologies, Ettlingen and Leipzig, Germany**)**, operating at 150.92 or 75.47 MHz for ^13^C and 600.13 or 300.13 MHz for ^1^H nuclei. The NMR spectra were taken in CDCl_3_ or DMSO-d_6_ at ambient temperature using TMS as a reference. Multiplets are abbreviated as follows: br–*broad*; s–*singlet*; d–*doublet*; t–*triplet*; q–*quartet*; m–*multiplet*. Infrared spectra were recorded with a Bruker ABB Bomem FTIR spectrometer (Bruker Technologies, Ettlingen and Leipzig, Germany). Melting points were determined using an Electrothermal 9100 apparatus in open capillaries and are uncorrected. High-resolution mass spectrometry (HRMS) was performed on an Agilent 6545 LC/Q-TOF MS (Agilent Technologies, Waldbronn, Germany) operating in a positive electrospray ionization (ESI) mode. The samples were dissolved in a mixture of matanol/acetonitrile (9/1) and injected directly into the MS detector in 70% acetonitrile with 0.1% formic acid and 30% water with 0.1% formic acid, flow rate 0.2 mL min^−1^. Recording and data processing were performed in Agilent MassHunter software (Agilent Technologies, Waldbronn, Germany).

The NMR spectra of new compounds are given in Supplementary Materials.

### 3.2. Chromatographic Conditions

In the HPLC mode, the mobile phase flow was 1.0 mL min^−1^, and the column operating temperature was 35 °C. By changing the volume fraction and type of alcohol (methanol or ethanol) in the CO_2_ mobile phase, the optimal chromatographic conditions for the separation of enantiomers in the SFC mode were determined. The mobile phase flow was 4.0 mL min^−1^, and the operating temperature of the column was 35 °C, with the return pressure of 15 MPa. In both modes, chromatogram recording was performed at a wavelength of 254 nm, and a full UV range from 190 nm to 400 nm was recorded (DAD detector, Agilent Technologies, Waldbronn, Germany).

### 3.3. Sample Preparation

Marinoepoxides *rac*-**6a**–**c** and *rac*-**8a**–**c** were dissolved in appropriate amounts of mobile phase used in analysis. The solutions were all filtered before sample injection. The concentration of solutions was 0.5 mg/mL.

## 4. Conclusions

In this work, we have demonstrated how the key chiral intermediate, marinoepoxide **6a**, in the synthesis of marine natural products marinoaziridines A and B, can be successfully achieved in its enantiopure form using a combination of organic synthesis and enantioselective chromatography with green solvents. Marinoepoxide *rac*-**6a** was synthesized through the convergent approach based on addition of the achiral sulfonium ylide, derived in situ from sulfonium salt **5** in presence of a base, to a carbon-oxygen double bond of the corresponding aldehydes **4a**–**c**, in 3.7% overall yield over five steps. Five other new marinoepoxide structures *rac*-**6b**–**c** and *rac*-**8a**–**c** were also synthesized using similar methodology. The enantioseparation of trisubstituted marinoepoxides *rac*-**6a**–**c** and disubstituted marinoepoxides *rac*-**8a**–**c** was successfully tested using two immobilized polysaccharides-based CSPs (Amylose-SA, and Cellulose-SC) by HPLC (using *n*-hexane and DMC), and by SFC (using supercritical carbon dioxide). We have demonstrated that green solvents, DMC and supercritical carbon dioxide, can be efficiently used as a mobile phase in chiral separation of marinoepoxides *rac*-**6a**–**c** and *rac*-**8a**–**c** on the immobilized polysaccharide-based CSPs. This finding contributes to the ecological initiative of replacing hydrocarbon-based mobile phases in liquid chromatography. This approach provides a new solution to prepare its derivatives and structurally related natural products.

## Figures and Tables

**Table 1:** Yields and diastereomeric ratios obtained in the reaction of the preparation of marinoepoxides *rac*-**8a**–**c**.

Marinoepoxide	Yield (%)	dr (*cis*/*trans*)
*rac*-**8a**	79	76 (12/88)
*rac*-**8b**	75	74 (13/87)
*rac*-**8c**	86	88 (6/94)

**Table 2:** Overview of immobilized polysaccharide chiral stationary phases based on cellulose and amylose derivatives.

Chiral Selector	Structure of the Chiral Selector		Commercial Name
cellulose *tris*-(3,5-dichlorophenylcarbamate)	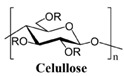	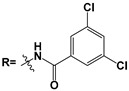	CHIRAL ART Cellulose-SC
cellulose *tris*-(3,5 dimethylphenylcarbamate)		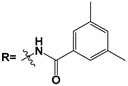	CHIRAL ART Cellulose-SB
amylose *tris*-(3,5 dimethylphenylcarbamate)	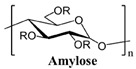	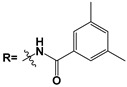	CHIRAL ART Amylose-SA

## Data Availability

Data are contained within the article.

## Supplementary Materials

The following are available online at https://www.mdpi.com/article/10.3390/md20080530/s1, Figure S1: ^1^H NMR spectrum (CDCl_3_, 300 MHz) of compound **1c**, Figure S2: ^13^C NMR spectrum (CDCl_3_, 151 MHz) of compound **1c**, Figure S3: ^1^H NMR spectrum (DMSO-*d_6_*, 600 MHz) of compound **3a**, Figure S4: ^13^C NMR spectrum (DMSO-*d_6_*, 151 MHz) of compound **3a**, Figure S5: ^1^H NMR spectrum (CDCl_3_, 300 MHz) of compound **3b**, Figure S6: ^13^C NMR spectrum (CDCl_3_, 75 MHz) of compound **3b**, Figure S7: ^1^H NMR spectrum (CDCl_3_, 300 MHz) of compound **3c**, Figure S8: ^13^C NMR spectrum (CDCl_3_, 151 MHz) of compound **3c**, Figure S9: ^1^H NMR spectrum(CDCl_3_, 300 MHz) of compound **4a**, Figure S10: ^13^C NMR spectrum (DMSO-*d6*, 75 MHz) of compound **4a**, Figure S11: ^1^H NMR (DMSO-*d6*, 300 MHz) of compound **4b**, Figure S12: ^13^C NMR spectrum (DMSO-*d6*, 151 MHz) of compound **4b**, Figure S13: ^1^H NMR spectrum(CDCl_3_, 300 MHz) of compound **4c**, Figure S14: ^13^C NMR spectrum (CDCl_3_, 151 MHz) of compound **4c,** Figure S15: ^1^H NMR spectrum (CDCl_3_, 300 MHz) of compound **5**, Figure S16: ^13^C NMR spectrum (CDCl_3_, 75 MHz) of compound **5**, Figure S17: ^1^H NMR spectrum (CDCl_3_, 300 MHz) of compound *rac*-**6a**, Figure S18: ^13^C NMR spectrum (CDCl_3_, 75 MHz) of compound *rac*-**6a**, Figure S19: ^1^H NMR spectrum(CDCl_3_, 600 MHz) of compound *rac*-**6b**, Figure S20: ^13^C NMR spectrum (CDCl_3_, 151 MHz) of compound *ra*c-**6b**, Figure S21: ^1^H NMR spectrum (CDCl_3_, 300 MHz) of compound *rac*-**6c**, Figure S22: ^13^C NMR spectrum (CDCl_3_, 75 MHz) of compound *rac*-**6c**, Figure S23: ^1^H NMR spectrum (CDCl_3_, 300 MHz) of compound **7**, Figure S24: ^13^C NMR spectrum (CDCl_3_, 75 MHz) of compound **7**, Figure S25: ^1^H NMR spectrum (CDCl_3_, 600 MHz) of diastereomeric mixture of compound *rac*-**8**, Figure S26: ^1^H NMR spectrum (CDCl_3_, 600 MHz) of the major diastereomer of compound *rac*-**8a**, Figure S27: ^13^C NMR spectrum (CDCl_3_, 151 MHz) of the major diastereomer of compound *rac*-**8a**, Figure S28: ^1^H NMR spectrum (CDCl_3_, 300 MHz) of diastereomeric mixture of compound *rac*-**8b**, Figure S29: ^1^H NMR spectrum (CDCl_3_, 300 MHz) of the major diastereomer of compound *rac-***8b**, Figure S30: ^13^C NMR spectrum (CDCl_3_, 75 MHz) of the major diastereomer of compound *rac*-**8b**, Figure S31: ^1^H NMR spectrum (CDCl_3_, 300 MHz) of diastereomeric mixture of compound *rac*-**8c**, Figure S32: ^1^H NMR spectrum (CDCl_3_, 300 MHz) of the major diastereomer of compound *rac*-**8c**, Figure S33: ^13^C NMR spectrum (CDCl_3_, 75 MHz) of the major diastereomer of compound *rac*-**8c**. Figure S34: IR spectrum of compound *rac*-**6a**, Figure S35: IR spectrum of compound *rac*-**6b**, Figure S36: IR spectrum of compound *rac*-**6c**, Figure S37: IR spectrum of compound *rac*-**8a,** Figure S38: IR spectrum of compound *rac*-**8b**, Figure S39: IR spectrum of compound *rac*-**8c**. Table S1: Chromatographic parameters for HPLC and SFC enantioseparation of *rac*-**6a**–**c**, Table S2: Chromatographic parameters for HPLC and SFC enantioseparation of *rac*-**8a**–**c [48,49,50,51]**.
