# Supplementary material for: Application of Green Chiral Chromatography in Enantioseparation of Newly Synthesized Racemic Marinoepoxides
Source: Mar Drugs. 2022 Aug 19;20(8):530. doi: 10.3390/md20080530 (PMC9409916; doi:10.3390/md20080530)
Supplement: Supplementary file 1 [file marinedrugs-20-00530-s001.zip › marinedrugs-1861881-supplementary.pdf]

Supplementary Materials

Application of green chiral chromatography in enantioseparation of newly synthesized racemic marinoepoxides

Anđela Buljan [1] and Marin Roje [1,*]

[1] Laboratory for Chiral Technologies, Department of organic chemistry and biochemistry, Ruđer Bošković Institute, Bijenička cesta 54, 10000 Zagreb, Croatia; Andjela.Buljan@irb.hr (A.B.)

* Correspondence: Marin.Roje@irb.hr; Tel.: +385-01-4571-283

Table of contents

1.1. **Preparation of marinoepoxides** ***rac*****-6a-c and** ***rac*****-8a-c** S4

Figure S1. ^{1}H NMR spectrum ($CDCl_3$, 300 MHz) of compound **1c** S14

Figure S2. ^{13}C NMR spectrum ($CDCl_3$, 151 MHz) of compound **1c** S14

Figure S3. ^{1}H NMR spectrum (DMSO-d6, 600 MHz) of compound **3a** S15

Figure S4. ^{13}C NMR spectrum (DMSO-d6, 151 MHz) of compound **3a** S15

Figure S5. ^{1}H NMR spectrum ($CDCl_3$, 300 MHz) of compound **3b** S16

Figure S6. ^{13}C NMR spectrum ($CDCl_3$, 75 MHz) of compound **3b** S16

Figure S7. ^{1}H NMR spectrum ($CDCl_3$, 300 MHz) of compound **3c** S17

Figure S8. ^{13}C NMR spectrum ($CDCl_3$, 151 MHz) of compound **3c** S17

Figure S9. ^{1}H NMR spectrum ($CDCl_3$, 300 MHz) of compound **4a** S18

Figure S10. ^{13}C NMR spectrum (DMSO-d_6, 75 MHz) of compound **4a** S18

Figure S11. ^{1}H NMR spectrum (DMSO-d_6, 300 MHz) of compound **4b** S19

Figure S12 ^{13}C NMR spectrum (DMSO-d_6, 151 MHz) of compound **4b** S19

Figure S13. ^{1}H NMR spectrum ($CDCl_3$, 300 MHz) of compound **4c** S20

Figure S14. ^{13}C NMR spectrum ($CDCl_3$, 151 MHz) of compound **4c** S20

Figure S15. ^{1}H NMR spectrum ($CDCl_3$, 300 MHz) of compound **5** S21

Figure S16. ^{13}C NMR spectrum ($CDCl_3$, 75 MHz) of compound **5** S21

Figure S17. ^{1}H NMR spectrum ($CDCl_3$, 300 MHz) of compound *rac*-**6a** S22

Figure S18. ^{13}C NMR spectrum ($CDCl_3$, 75 MHz) of compound *rac*-**6a** S22

Figure S19. ^{1}H NMR spectrum ($CDCl_3$, 600 MHz) of compound *rac*-**6b** S23

Figure S20. ^{13}C NMR spectrum ($CDCl_3$, 151 MHz) of compound *rac*-**6b** S23

Figure S21. ^{1}H NMR spectrum ($CDCl_3$, 300 MHz) of compound *rac*-**6c** S24

Figure S22. ^{13}C NMR spectrum ($CDCl_3$, 75 MHz) of compound *rac*-**6c** S24

Figure S23. ^{1}H NMR spectrum ($CDCl_3$, 300 MHz) of compound **7** S25

Figure S24. ^{13}C NMR spectrum ($CDCl_3$, 75 MHz) of compound **7** S25

Figure S25. ^{1}H NMR spectrum ($CDCl_3$, 600 MHz) of diastereomeric mixture of compound *rac*-**8a** S26

Figure S26. ^{1}H NMR spectrum ($CDCl_3$, 600 MHz) of the major diastereomer of compound *rac*-**8a** S26

Figure S27. ^{13}C NMR spectrum ($CDCl_3$, 151 MHz) of the major diastereomer of compound *rac*-**8a**..... S27

Figure S28. ^{1}H NMR spectrum ($CDCL_3$, 300 MHz) of diastereomeric mixture of compound *rac*-**8b** .. S28

Figure S29. ^{1}H NMR spectrum ($CDCl_3$, 300 MHz) of the major diastereomer of compound *rac*-**8b** S28

Figure S30. ^{13}C NMR spectrum ($CDCl_3$, 75 MHz) of the major diastereomer of compound *rac*-**8b** S29

Figure S31. ^{1}H NMR ($CDCl_3$, 300 MHz) of diastereomeric mixture of compound *rac*-**8c**...................... S30

Figure S32. ^{1}H NMR spectrum ($CDCl_3$, 300 MHz) of the major diastereomer of compound *rac*-**8c**.......... S30

Figure S33. ^{13}C NMR spectrum ($CDCl_3$, 75 MHz) of the major diastereomer of compound *rac*-**8c** S31

Figure S34. IR spectrum of compound rac-**6a** S32

Figure S35. IR spectrum of compound rac-**6b** S32

Figure S36. IR spectrum of compound *rac*-**6c** S33

Figure S37. IR spectrum of compound *rac*-**8a** S33

Figure S38. IR spectrum of compound *rac*-**8b** S34

Figure S39. IR spectrum of compound *rac*-**8c** S34

Table S1.. S35

Table S2.. S36

References.. S36

1.1. Preparation of marinoepoxides *rac*-6a-c and *rac*-8a-c

Preparation of *N*-benzylaniline 1c: Following a reported procedure [1], a mixture of aniline (12 mL, 129 mmol), $NaHCO_3$ (3.42, 40 mmol) and H_2O (3.6 mL) was heated to 95 °C before the addition of freshly distilled benzyl chloride (3.9 mL, 33.3 mmol). The reaction mixture was stirred at that temperature for 4 h, upon which HPLC analysis of an aliquot of the reaction mixture indicated complete consumption of the benzyl chloride. The mixture was then poured into ice water. The aqueous layer was extracted with dichloromethane (3 x 100 mL). The combined organic layers were washed with brine (100 mL), dried over anhydrous Na_2SO_4, filtered and concentrated under reduced pressure. The crude product was purified *via* column chromatography (*flash* silica gel, petroleum ether/ethyl acetate = 30/1). Pure product **1c** was isolated as a light yellow oil (5.19 g, 86% yield).

Data for *N*-benzylaniline 1c:

^{1}H NMR ($CDCl_3$, 300 MHz) δ/ppm**:** 4.01 (1H, s), 4.32 (s, 2H), 6.58-6.67 (m, 2H), 6.71 (tt, J_1 = 1.0 Hz, J_2 = 7.3 Hz, 1H), 7.13-7.21 (m, 2H), 7.22-7.40 (m, 5H).

^{13}C ($CDCl_3$, 151 MHz) δ/ppm: 48.35, 112.86 (2C), 117.58, 127.25 (2C), 127.53, 128.66 (2C), 129.29 (2C), 139.46, 148.18.

HRMS calculated for $C_{13}H_{13}N$ $[M+H]^+$ 184.11262, found 184.11299.

General procedure 1 for the synthesis of quinoline-2(1*H*)-ones 3a,c: A mixture of aniline derivatives **1a,1c** (1 equiv.) and ethyl-3-oxobutanoate (2 equiv.) was stirred at 150 °C for 1h (keeping the power constant at 250 W) under microwave irradiation. The course of the reaction was monitored using HPLC which indicated product formation. The excess of 3-oxobutanoate was evaporated in *vacuo* to give a yellow oil **2a,c** which was used without further purification. The residue was heated in H_2SO_4 (23 mL, 70%) at 95 °C. After 1h the HPLC analysis indicated complete consumption of starting material. The mixture was then diluted with water, pH was adjusted to ~7 by addition of aqueous NaOH (6M). The aqueous layer was extracted with ethyl acetate (3 x 50 mL). The combined organic layers were washed with brine, dried over anhydrous Na_2SO_4, filtered and concentrated under reduced pressure. The crude product was purified *via* flash column chromatography (dichloromethane/methanol = 25/1).

Preparation of 4-methylquinoline-2(1*H*)-one 3a:

According to the general procedure 1, starting from aniline **1a** (1.0 g, 10.7 mmol) and ethyl-3-oxobutanoate (2.80 g, 20.14 mmol), the compound **3a** was obtained as a yellow solid (0,66 g, 40 % yield)

Data for 4-methylquinoline-2(1*H*)-one 3a:

m.p. = 217.1-218.9 °C

^{1}H NMR (DMSO-d6, 600 MHz) δ/ppm: 2.40 (d, J_1 = 1.2 Hz, 3H), 6.38 (q, J_1 = 1.4 Hz, 1H), 7.17 (ddd, J_1 = 1.2 Hz, J_2 = 7.3 Hz, J_3 = 8.3 Hz, 1H), 7.30 (dd, J_1 = 1.2 Hz, J_2 = 8.2 Hz, 1H), 7.48 (ddd, J_1 = 1.5 Hz, J_2 = 7.3 Hz,

J_3 = 8.5 Hz, 1H), 7.68 (dd, J_1 = 1.3 Hz, J_2 = 8.2 Hz, 1H), 11.57 (s, 1H).

^{13}C ($CDCl_3$, 151 MHz) *δ*/ppm: 18.89, 115.87, 120.05, 121.30, 122.09, 125.15, 130.71, 139.11, 148.36, 162.09.

Preparation of 1,4-dimethylquinoline-2(1*H*)-one 3b: Following a reported procedure [2], a mixture of *N*-methylaniline (6 mL, 55.38 mmol) and ethyl-3-oxobutanoate (14.01 mL, 110.76 mmol) was heated at reflux in acetic acid (30 mL) for 48 h. HPLC indicated complete consumption of the amine starting material. The excess of ethyl acetoacetate was evaporated in *vacuo* to give a yellow oil **2b** which was used without further purification. The residue was heated in H_2SO_4 (24 mL, 70%) at 95°C. HPLC after 1 h indicated complete consumption of starting material. The mixture was then diluted with water, pH was adjusted to ~7 by addition of aqueous NaOH (6M). The aqueous layer was extracted with ethyl acetate (3 x 50 mL). The combined organic layers were washed with brine, dried over anhydrous Na_2SO_4, filtered and concentrated under reduced pressure. The crude NMR showed ~55% conversion of SM to **3b**. The regioisomeric side product was observed on the crude NMR. The crude product was purified *via* column chromatography (*flash* silica gel, dichloromethane/methanol = 25/1). Pure product **3b** was isolated as a white solid (2.54 g, 27% yield).

Data for 1,4-dimethylquinoline-2(1*H*)-one 3b:

m.p. = 131.8-133.1 °C

^{1}H NMR ($CDCl_3$, 300 MHz) δ/ppm: 2.47 (s, 3H), 3.71 (s, 3H), 6.61 (s, 1H), 7.26 (d, J_1 = 15.0 Hz, 1H), 7.38 (d, J_1 = 8.4 Hz, 1H), 7,57 (t, J_1 = 7.7 Hz, 1H), 7.71 (d, J_1 = 8.0 Hz, 1H).

^{13}C ($CDCl_3$, 75 MHz) *δ*/ppm: 18.96, 29.23, 114.41, 121.17, 121.47, 121.90, 125.22, 130.45, 139.86, 146.37, 162.14.

IR $\tilde{\nu}$/ cm^{-1} = 2919, 2886, 2850, 1652, 1615, 1592, 1562, 1503, 1455, 1409, 1387, 1.371, 1322, 1268, 1162, 1139, 1113, 1066, 1041, 924, 874, 863, 843, 769, 753.

HRMS calculated for $C_{11}H_{11}NO$ $[M+H]^+$ 174.09189, found 174.09181.

Preparation of 1-benzyl-4-methylquinolin-2(1*H*)-one 3c: According to the general procedure 1, starting from compound **1c** (4.7 g, 26 mmol) and ethyl 3-oxobutanoate (6.5 mL, 52 mmol), compound **3c** was obtained as a white solid (3.15 g, 49% yield).

Data for 1-benzyl-4-methylquinoline-2(1*H*)-one 3c:

m.p. = 114.1-115.5 °C

^{1}H NMR ($CDCl_3$, 300 MHz) δ/ppm: 2.51 (d, J_1 = 1.0 Hz, 3H), 5.56 (s, 2H), 6.70 (d, J_1 = 0.8 Hz, 1H), 7.17-7.32 (m, 7H), 7.42 (ddd, J_1 = 1.5 Hz, J_2 = 7.1 Hz, J_3= 8.6 Hz, 1H), 7.71 (dd, J_1 = 1.3, J_2 = 8.0 Hz, 1H).

^{13}C ($CDCl_3$, 151 MHz) δ/ppm: 18.66, 45.18, 114.85, 120.51, 121.19, 121.52, 124.78, 126.03 (2C), 126.69, 128.28 (2C), 129.96, 136.05, 138.67, 146.57, 161.77.

IR ṽ/cm^{-1}: 3316, 3141, 3098, 3017, 2968, 2891, 2856, 2741, 1669,1651, 1594, 1511, 1473, 1435, 1403, 1267, 1206, 1145, 1130, 982.

HRMS calculated for $C_{17}H_{15}NO$ $[M+H]^+$ 250.12319, found 250.12365.

Preparation of 2-oxo-1,2-dihydroquinoline-4-carbaldehyde 4a: Following a reported procedure [3], to a suspension of compound **3a** (2.0 g, 12.6 mmol) and molecular sieves (5 Å, powder, activated, 3.8 g) in xylene (30 mL) at 150 °C was added selenium dioxide (9.76 g, 88.1 mmol) under an atmosphere of argon. The mixture was further stirred at reflux for 24 h, upon which HPLC analysis of an aliquot of the reaction mixture indicated complete consumption of starting material. The crude NMR showed ~65% conversion of SM to **4a**. The alcohol side product was observed on the crude NMR. The crude product was purified *via* column chromatography (*flash* silica gel, chloroform/methanol = 5/1). The solvent was removed in *vacuo* to give a dark yellow solid which was recrystallized from methanol to give a yellow solid **4a** (1.20 g, 55% yield).

Data for 2-oxo-1,2-dihydroquinoline-4-carbaldehyde 4a:

m.p. =214.2-216.4 °C

^{1}H NMR ($CDCl_3$, 300 MHz) δ/ppm: 7.16 (s, 1H), 7.35 (d, J_1 = 8.0 Hz, 1H), 7.60 (m, 1H), 8.76 (d, J_1 = 7.7 Hz, 1H), 10.21 (s, 1H), 10.88 (s, 1H).

^{13}C (DMSO-d_6, 75 MHz) δ/ppm: 114.93, 115.67, 122.66, 125.17, 131.02, 131.61, 139.61, 141.18, 161.37, 194.74.

IR ṽ/cm^{-1}:

HRMS calculated for $C_{10}H_7NO_2$ $[M+H]^+$ 174.05550, found 174.05535.

General procedure 2 for the synthesis of quinoline-2(1*H*)-one-4-carbaldehydes 4b,c: A closed tube was charged with compounds **3b,c** (1 equiv.) and heated to 150 °C before the addition of selenium dioxide (1.5 equiv.). The resulting mixture was stirred at 175 °C for 2 h, until HPLC indicated complete consumption of starting materials. The mixture was allowed to cool to 40 °C and the resulting dark orange solid was dissolved in CH_2Cl_2 (40 mL) and filtered through a short pad of celite. The filtrate was concentrated *in vacuo* to give a crude product which was purified *via* column chromatography or recrystallized from the appropriate solvent.

Preparation of 1-methyl-2-oxo-1,2-dihydroquinoline-4-carbaldehyde 4b: According to the general procedure 2, starting from compound **3b** (1.92 g, 11.1 mmol) and selenium dioxide (1.85 g, 16.6 mmol) in a closed system, compound **4b** was obtained as a yellow solid (1.30 g, 63% yield).

Data for 1-methyl-2-oxo-1,2-dihydroquinoline-4-carbaldehyde 4b:

m.p. = 187.1-189.2 °C

^{1}H NMR (DMSO-d_6, 300 MHz) δ/ppm: 3.67 (s, 3H), 7.28 (s, 1H), 7.36 (ddd, J_1 = 1.2 Hz, J_2 = 7.1 Hz, J_3 = 8.2 Hz, 1H), 7.62 (dd, J_1 = 0.7 Hz, J_2 = 8.4 Hz, 1H), 7.71 (ddd, J_1 = 1.5 Hz, J_2 = 7.1 Hz, J_3 = 8.6 Hz, 1H), 8.67 (dd, J_1 = 1.4 Hz, J_2 = 8.5 Hz, 1H), 10.19 (s, 1H).

^{13}C (DMSO-d_6, 151 MHz) δ/ppm: 30.04, 115.74, 116.39, 123.29, 126.09, 130.93, 131.92, 140.18, 140.70, 161.15, 195.05.

IR $\tilde{\nu}_{max}$/ cm^{-1}: 3024, 2882, 2772, 1698, 1659, 1587, 1453, 1412, 1392, 1326, 1053, 937, 898, 750, 656, 518, 468.

HRMS calculated for $C_{11}H_9NO_2$ $[M+H]^+$ 188.07115, found 188.07098.

Preparation of 1-benzyl-2-oxo-1,2-dihydroquinoline-4-carbaldehyde 4c: According to the general procedure, starting from compound **3c** (1.33 g, 5.34 mmol) and selenium dioxide (0.89 g, 8.0 mmol) in a closed system, compound **4c** was obtained as a orange solid (1.35 g, 96% yield).

Date for 1-benzyl-2-oxo-1,2-dihydroquinoline-4-carbaldehyde 4c:

m.p. = 84.7-86.1 °C

^{1}H NMR ($CDCl_3$, 300 MHz) δ/ppm: 5.60 (s, 2H), 7.19-7.37 (m, 8H), 7.50 (ddd, J_1 = 1.6 Hz, J_2 = 7.1 Hz, J_3 = 8.7 Hz, 1H), 8.84 (dd, J_1 = 1.5 Hz, J_2 = 8.2 Hz, 1H), 10.20 (s, 1H).

^{13}C ($CDCl_3$, 151 MHz) δ/ppm: 45.94, 114.88, 116.27, 122.91, 126.03 (2C), 126.17, 127.05, 128.44 (2C), 131.05, 131.10, 135.11, 139.51, 140.05, 161.32, 192.22.

IR (KBr) $\tilde{\nu}$/cm^{-1}: 3313, 3132, 3045, 3010, 2940, 2851, 2810, 2741, 1730, 1717, 1669,1651, 1594, 1511, 1473, 1435, 1403, 1267, 1206, 1145, 1130, 982.

HRMS calculated for $C_{17}H_{13}NO_2$ $[M+H]^+$ 264.10245, found 265.10533.

Preparation of isopropyl(diphenyl)sulfonium tetrafluoroborate 5: Following a reported procedure [4], to a mixture of 2-iodopropane (3.0 g, 17.6 mmol) and diphenyl sulfide (2.65 mL, 15.9 mmol) in dry dichloromethane (20 mL) was added $AgBF_4$ (3.43 g, 15.9 mmol) under ice bath cooling. The mixture was covered with aluminium foil. The solution turned white and a white solution started to precipitate, and then the resulting mixture was stirred 24 h under an atmosphere argon, until TLC indicated complete consumption of starting material. The resulting suspension was filtered through a short pad of celite. The filtrate was concentrated *in vacuo* to give a yellow oil. After being washed with ether, the residue was dried under reduced pressure and recrystallized from dichloromethane/diethyl ethere = ¼ to provide the sulfonium salt **5** (2.5 g, 50% yield) as white crystals.

Data for isopropyl(diphenyl)sulfonium tetrafluoroborate 5:

m.p. = 122.3-123.5 °C

^{1}H NMR ($CDCl_3$, 300 MHz) δ/ppm: 1.50 (d, J_1 = 6.6 Hz, 6H), 5.04 (p, 1H), 7.57-7.79 (m, 6H), 8.02-8.20 (m, 4H).

^{13}C ($CDCl_3$, 75 MHz) δ/ppm: 18.54 (2C), 50.68, 123.81 (2C), 131.33 (4C), 131.66 (4C), 134.83 (2C).

General procedure 3 for the synthesis of racemic 2-quinolinone trisubstituted marino**epoxides** rac-**6a-c:** To a flame dried Schlenk flask was added isopropyl(diphenyl)sulfonium tetrafluoroborate **5** (1 equiv.), which was stirred under vacuum for 0.5 h and then suspended in dry freshly distilled THF under an atmosphere of argon. The mixture was cooled to -78 and treated dropwise with *t*-BuLi (1.2 equiv., c = 1.9 M) to give a orange solution. The reaction mixture was stirred for 20 minutes before the addition of the appropriate aldehydes **4a-c** (1 equiv.). After the addition was complete, the solution was heated to -

50 °C, and then was stirred for 4 h. The reaction was quenched by the addition of water (10 mL). The aqueous layer was extracted with ethyl acetate (3 x 20 mL). The combined organic layers were washed with brine (20 mL), dried over anhydrous Na_2SO_4 and filtered. The solvent was removed in *vacuo* to give a dark orange solid which was purified *via* column chromatography (*flash* silica gel, dichloromethane/methanol = 25/1). Pure products *rac*-**6a-c** were isolated as white solids.

Preparation of racemic 4-(3,3-dimethyloxiran-2-yl)quinolin-2(1*H*)-one *rac*-6a: According to the general procedure 3, starting from aldehyde **4a** (0.32 g, 1.8 mmol), isopropyl(diphenyl)sulfonium tetrafluoroborate **5** (1,3 g, 4.0 mmol) and *t*-BuLi (2.5 mL, 4.8 mmol) in dry THF (70 mL), compound rac-**6a** was obtained as a white solid (0.058 g, 14.6% yield).

Data for racemic 4-(3,3-dimethyloxiran-2-yl)quinolin-2(1*H*)-one *rac*-6a:

^{1}H NMR ($CDCl_3$, 300 MHz) δ/ppm: 1.14 (s, 3H), 1.65 (s, 3H), 4.05 (d, J = 0,3 Hz, 1H), 6,71 (d, J = 0,4 Hz, 1H), 7.26 (dd, J_1 = 0.9 Hz, J_2 = 15.3 Hz, 1H), 7.40 (d, J_1 = 8.2 Hz, 1H), 7.55 (ddd, J_1 = 1.3 Hz, J_2 = 7.2 Hz, J_3 = 8.3 Hz, 1H), 7.63 (dd, J_1 = 1.3 Hz, J_2 = 8.0 Hz, 1 H), 11.32 (s, 1H).

^{13}C ($CDCl_3$, 75 MHz) δ/ppm: 17.05, 23.45, 60.54, 60.68, 115.81, 117.55, 117.60, 121.78, 122.39, 129.81, 137.35, 146.34, 163.15.

IR (KBr) ṽ/cm^{-1}: 2983, 2963, 2850, 1651, 1612, 1557, 1437, 1373, 1190, 1119, 912, 885, 719, 695, 508.

HRMS calculated for $C_{13}H_{13}NO_2$ $[M+H]^+$ 216.10245, found 216.10226.

Preparation of racemic 4-(3,3-dimethyloxiran-2-yl)-1-methylquinolin-2(1*H*)-one *rac*-6b: According to the general procedure 3, starting from aldehyde **4b** (0.47 g, 2.5 mmol), isopropyl(diphenyl)sulfonium tetrafluoroborate **5** (0.80 g, 2.5 mmol) and *t*-BuLi (1.6 mL, 3.0 mmol) in dry THF (15mL), compound *rac*-**6b** was obtained as a white solid (0.20 g, 35% yield).

Data for racemic 4-(3,3-dimethyloxiran-2-yl)-1-methylquinolin-2(1*H*)-one *rac*-6b:

m.p. = 92,9 - 94,8 °C

^{1}H NMR ($CDCl_3$, 600 MHz) δ/ppm: 1.10 (s, 3H), 1.62 (s, 3H), 3.72 (s, 3H), 4,00 (d, J_1 = 0.9 Hz, 1H), 6,71 (d, J_1 = 0.9 Hz, 1H), 7.26-7.29 (m, 1H), 7,41 (d, J_1 = 8,3 Hz, 1H), 7, 60 (ddd, J_1 = 1.5 Hz, J_2 = 7.1 Hz, J_3 = 8.6 Hz, 1H), 7,64 (d, J_1 = 1.5 Hz, J_2 = 7.9 Hz, 1H).

^{13}C ($CDCl_3$, 151 MHz) δ/ppm: 18.07, 24.41, 29.36, 61.38, 61.55, 114.75, 119.22, 119.36, 122.14, 124.17, 130.75, 139.88, 144.56, 161.94.

IR ṽ/cm^{-1}: 2961, 2923, 1649, 1590, 1565, 1504, 1453, 1365, 1320, 1250, 1119, 1071, 933, 795, 750, 728, 693, 461.

HRMS calculated for $C_{14}H_{15}NO_2$ $[M+H]^+$ 230.11810, found 230.11866.

Preparation of racemic 1-benzyl-4-(3,3-dimethyloxiran-2-yl)quinolin-2(1*H*)-one *rac*-6c: According to the general procedure 3, starting from aldehyde **4c** (0.32 g, 1.2 mmol), isopropyl(diphenyl)sulfonium tetrafluoroborate **5** (0.38 g, 1.2 mmol) and *t*-BuLi (0.7 mL, 1.4 mmol) in dry THF (7 mL), compound *rac*-**6c** was obtained as a white solid (0.32 g, 86% yield).

Data for racemic 1-benzyl-4-(3,3-dimethyloxiran-2-yl)quinolin-2(1*H*)-one *rac*-6c:

m.p. = 140.0-142.0 °C

^{1}H NMR ($CDCl_3$, 300 MHz) δ/ppm: 1.16 (s, 3H), 1.65 (s, 3H), 4.03 (d, *J* = 1.1 Hz, 1H), 5.47 (d, *J* = 15.4 Hz, 1H), 5.65 (d, *J* = 16.2 Hz, 1H), 6.82 (d, *J* = 1.2 Hz, 1H); 7.19-7.26 (m, 4H), 7.26-7.35 (m, 3H), 7.46 (ddd, J_1 = 1.5 Hz, J_2 = 7.2 Hz, J_3 = 8.6 Hz, 1H).

^{13}C NMR ($CDCl_3$, 75 MHz) δ/ppm: 18.14, 24.44, 45.89, 61.49, 61.64, 115.65, 119.10, 119.60, 122.26, 124.24, 126.61 (2C), 127.30, 128.82 (2C), 130.75, 136.34, 139.29, 145.19, 162.10.

IR ṽ/cm^{-1}: 2978, 1652, 1596, 1567, 1497, 1450, 1366, 1316, 1253, 1304, 1160, 1073, 929, 800, 753, 725, 715, 696, 576, 510, 458.

HRMS calculated for $C_{20}H_{19}NO_2$ $[M+H]^+$ 306.14940, found 306.15009.

Preparation of benzyl(dimethyl)sulfonium perchlorate 7: To a mixture of benzyl chloride (0.84 g, 4.95 mmol) and dimethyl sulfide (0.37 g, 5.94 mmol) in dry dichloromethane (20 mL) was added $AgClO_4$ (1.03 g, 4.95 mmol) at room temperature under an atmosphere of argon. The mixture was covered with aluminium foil. The solution turned grey and a grey solution started to precipitate, and then the resulting mixture was stirred 4 h, until TLC indicated complete consumption of starting materials. The resulting suspension was filtered through a short pad of celite. The filtrate was concentrated *in vacuo* to give a yellow oil. After being washed with ether, the residue was dried under reduced pressure and recrystallized from diethyl ethere to provide the sulfonium salt **7** (1.18, 78% yield).

Data for benzyl(dimethyl)sulfonium perchlorate 7:

m.p. = 62.2-62.9 °C

^{1}H NMR ($CDCl_3$, 300 MHz) δ/ppm: 2,91 (s, 6H); 4,66 (s, 2H); 7,45 (m, 5H).

^{13}C ($CDCl_3$, 75 MHz) δ/ppm: 23.74 (2C), 45.75, 126.09, 129.85 (2C), 130.56, 130.86, 131.01.

General procedure 4 **for the synthesis of racemic 2-quinolinone disubstituted** marino**epoxides** ***rac*-8a-c:** To a flame dried Schlenk flask was added benzyl(dimethyl)sulfonium perchlorate **7** (1 equiv.), which was stirred under vacuum for 0.5 h, and then suspended in dry CH_3CN under an atmosphere of argon. Potassium hydroxide (2 equiv.) was added and the resulting orange solution was stirred 0.5 h at room temperature before addition of the corresponding aldehydes **4a-c** (1 equiv.). After the addition was complete, the resulting suspension was stirred overnight. The reaction was quenched by the addition of water (10 mL). The aqueous solution was extracted with ethyl acetate (3 x 20 mL). The combined organic layers were washed with brine (20 mL), dried over anhydrous Na_2SO_4 and filtered. The solvent was removed in *vacuo* to give a dark orange solid. ^{1} H NMR analysis of the crude reaction mixture indicated that the *trans* isomer predominates. The crude product was purified *via* column chromatography (*flash* silica gel, dichloromethane/methanol = 25/1). Pure products *rac*-**8a-c** were isolated as white solids.

Preparation of racemic 4-(3-phenyloxiran-2-yl)quinolin-2(1*H*)-one *rac*-8a: According to the general procedure 4, starting from aldehyde **4a** (0.09 g, 0.52 mmol), benzyl(dimethyl)sulfonium perchlorate **7** (0.16 g, 0.52 mmol) and KOH (0.06 g, 1.0 mmol) in dry CH_3CN (10 mL), compound *rac*-**8a** was obtained as a white solid (0.11 g, 79 % yield). The crude NMR showed d.r. *cis*/*trans* = 12/88 (Figure S25).

Data for racemic *trans*-4-(3-phenyloxiran-2-yl)quinoline-2(1*H*)-one *rac*-8a (major diastereomer):

m.p. = 254.9-257.0 °C

^{1}H NMR ($CDCl_3$, 600 MHz) δ/ppm: 3.87 (d, J_1 = 1.9 Hz, 1H), 4.25 (dd, J_1 = 1.0 Hz, J_2 = 2.0 Hz, 1H), 6.84 (d, J_1 = 1.0 Hz, 1H), 7.18-7.23 (m, 1H), 7.23-7.32 (m, 1H), 7.38-7.47 (m, 5H), 7.55 (ddd, J_1 = 1.3 Hz, J_2 = 7.2 Hz, J_3 = 8.4 Hz, 1H), 7.64 (dd, J_1 = 1.3 Hz, J_2 = 8.1 Hz), 11.61 (s, 1H).

^{13}C ($CDCl_3$, 151 MHz) δ/ppm: 59.18, 61.84, 116.51, 118.46, 122.88, 123.49, 125.67 (2C), 128.85 (2C), 128.94, 130.97, 136.04, 138.33, 147.55, 163.88.

IR $\tilde{\nu}_{max}$ / cm^{-1}: 3002, 2847, 1652, 1557, 1510, 1438, 1425, 1380, 1351, 1265, 1240, 983, 883, 768, 751, 703, 583.

HRMS calculated for $C_{17}H_{13}NO_2$ $[M+H]^+$ 264.10245, found 264.10257.

Preparation of racemic 1-methyl-4-(3-phenyloxiran-2-yl)quinolin-2(1*H*)-one *rac*-**8b:** According to the general procedure 4, starting from aldehyde **4b** (0.4 g, 2.1 mmol), benzyl(dimethyl)sulfonium perchlorate **7** (0.6 g, 2.1 mmol) and KOH (0.24 g, 4.2 mmol) in dry CH_3CN (12 mL), compound *rac*-**8b** was obtained as a white solid (0.4 g, 75 % yield). The crude NMR showed d.r. *cis*/*trans* = 13/87 (Figure S28).

Data for racemic *trans*-1-methyl-4-(3-phenyloxiran-2-yl)quinoline-2(1*H*)-one *rac*-8b (major diastereomer): m.p. = 198.1-200.9 °C

^{1}H NMR ($CDCl_3$, 300 MHz) δ/ppm: 3.74 (s, 3H), 3.84 (d, J_1 = 2.0 Hz, 1H), 4.21 (dd, J_1 = 0.9 Hz, J_2 = 2.0 Hz, 1H), 6.85 (d, J_1 = 0.9 Hz, 1H), 7.22 (td, J_1 = 0.8 Hz, J_2 = 8.1 Hz, 1H), 7.35-7.50 (m, 6H), 7.60 (ddd, J_1 = 1.5 Hz, J_2 = 7.2 Hz, J_3 = 8.6 Hz, 1H), 7.67 (dd, J_1 = 1.5 Hz, J_2 = 8.0 Hz, 1H).

^{13}C ($CDCl_3$, 75 MHz) δ/ppm: 29.43, 59.12, 61.60, 114.73, 117.02, 122.23, 124.16, 125.65 (2C), 128.82 (2C), 128.88, 130.94, 136.10, 139.95, 144.89, 162.10, 170.56.

IR $\tilde{\nu}_{max}$/ cm^{-1}: 3060, 1650, 1592, 1568, 1499, 1456, 1415, 1378, 1324, 1272, 1163, 1083, 934, 881, 753, 701, 644, 608, 701, 591, 507, 480.

HRMS calculated for $C_{18}H_{15}NO_2$ $[M+H]^+$ 278.11810, found 278.11784.

Preparation of racemic 1-benzyl-4-(3-phenyloxiran-2-yl)quinolin-2(1*H*)-one According to the general procedure 4, starting from aldehyde **4c** (0.15 g, 0.57 mmol), benzyl(dimethyl)sulfonium perchlorate **7** (0.17 g, 0.57 mmol) and KOH (0.06 g, 1.14 mmol) in dry CH_3CN (10 mL), compound *rac*-**8c** was obtained as a white solid (0.16 g, 80 % yield). The crude NMR showed d.r. *cis*/*trans* = 6/94 (Figure S31).

Data for racemic *trans*-1-benzyl-4-(3-phenyloxiran-2-yl)quinoline-2(1*H*)-one *rac*-8c (major diastereomer): m.p. = 161.4-163.6 °C

^{1}H NMR ($CDCl_3$, 300 MHz) δ/ppm: 3.89 (d, J_1 = 1.9 Hz, 1H), 4.23 (dd, J_1 = 1.0 Hz, J_2 = 2.0, 1H), 5.58 (s, 2H), 6.94 (s, 1H), 7.16 (td, J_1 = 1.1 Hz, J_2 = 8.1 Hz, 1H), 7.20-7.26 (m, 3H), 7.26-7.35 (m, 3H), 7.35-7.50 (m, 6H), 7.66 (dd, J_1 = 1.6 Hz, J_2 = 8.2 Hz, 1H).

^{13}C NMR ($CDCl_3$, 75 MHz) δ/ppm: 45.92, 59.19, 61.64, 115.66, 116.94, 119.50, 122.32, 124.23, 125.67 (2C), 126.60 (2C), 127.33, 128.84 (4C), 128.92, 130.88, 136.09, 136.28, 139.36, 145.49, 162,27.

IR $\tilde{\nu}_{max}$/ cm^{-1}: 3034, 1652, 1590, 1564, 1496, 1454, 1379, 1314, 1073, 1029, 898, 840, 752, 723, 694, 594, 505.

HRMS calculated for $C_{24}H_{19}NO_2$ $[M+H]^+$ 354.14940, found 354.15016.

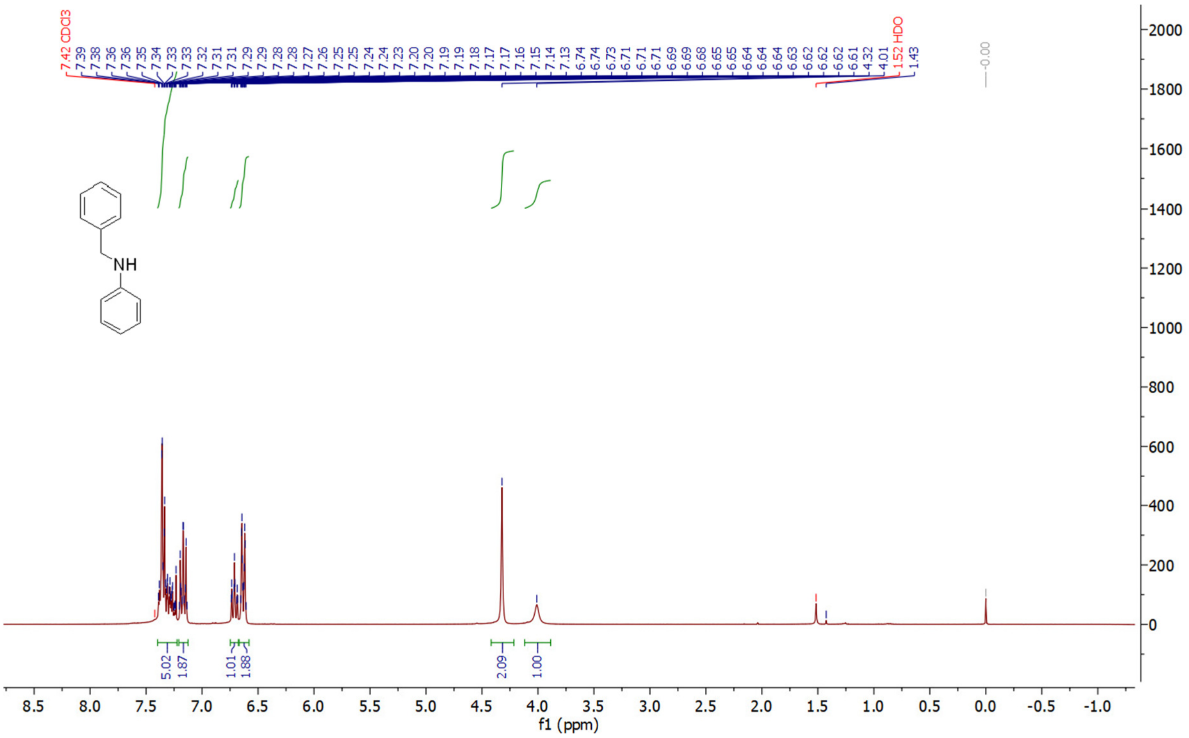

Figure S1. ^{1}H NMR spectrum ($CDCl_3$, 300 MHz) of compound **1c**.

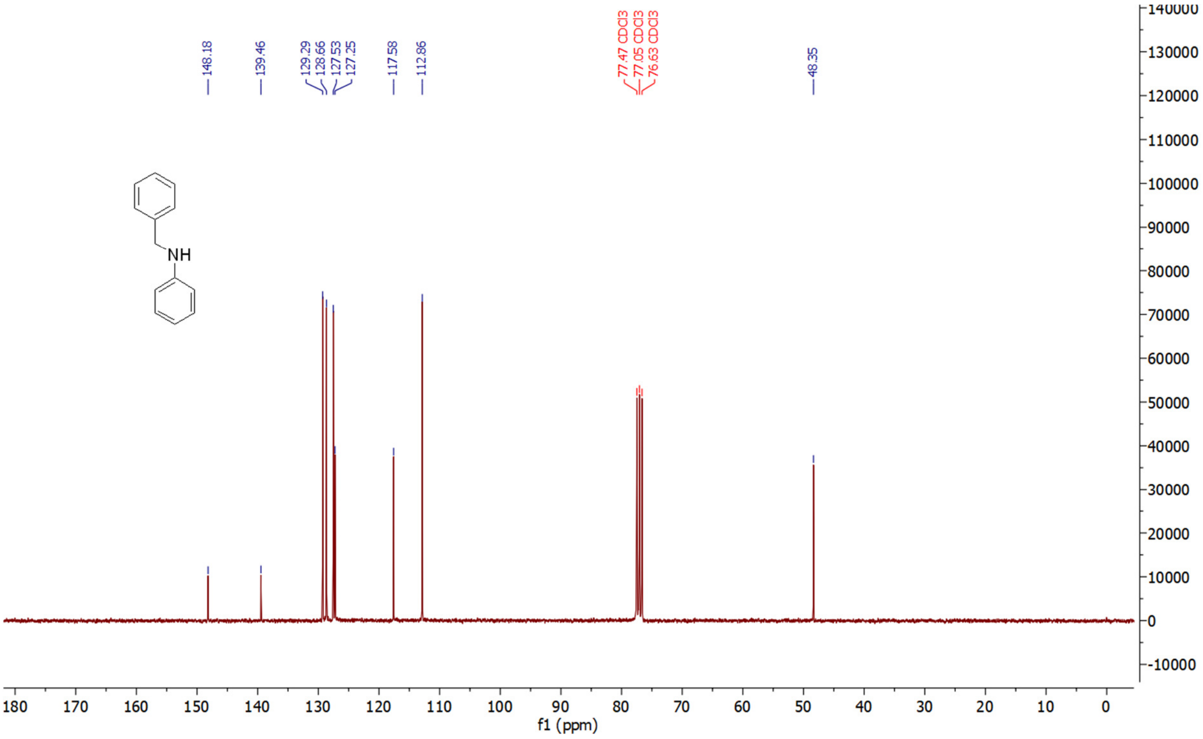

Figure S2. ^{13}C NMR spectrum ($CDCl_3$, 151 MHz) of compound **1c**.

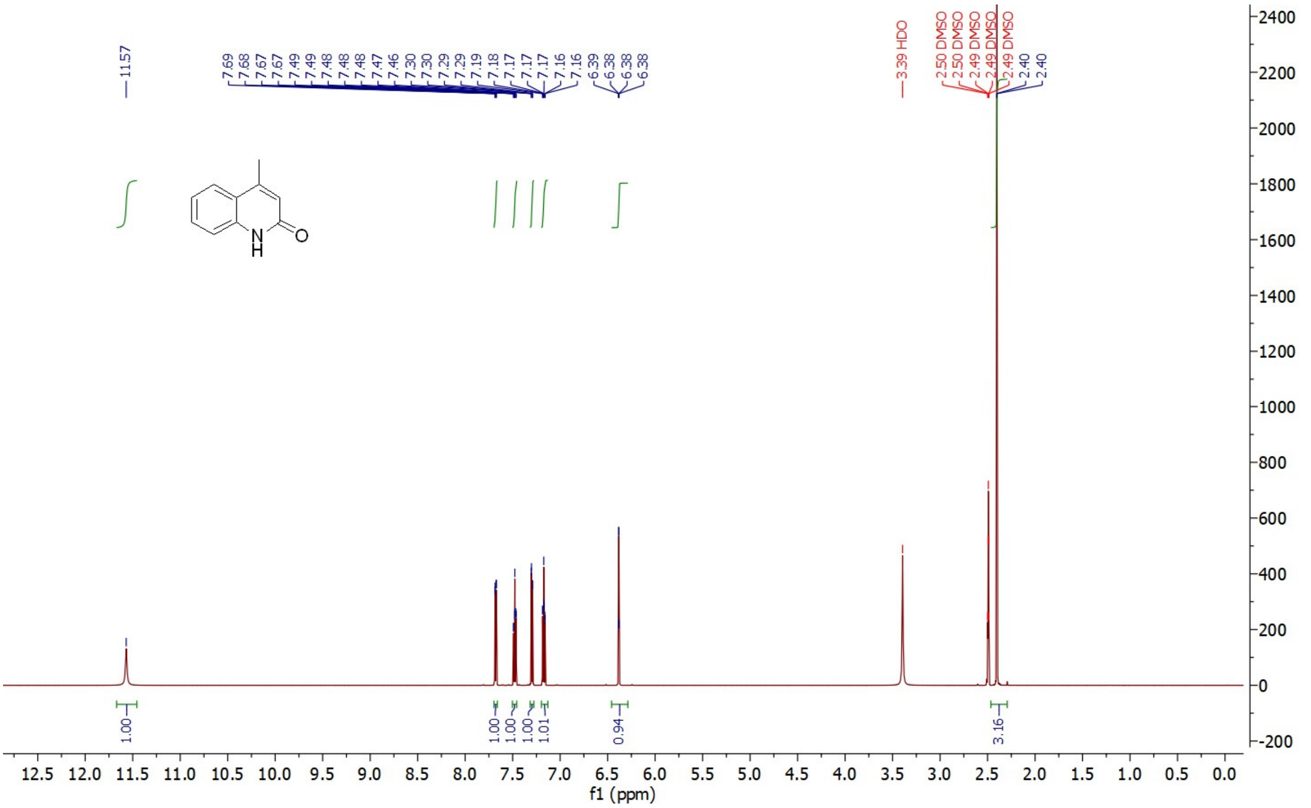

Figure S3. ^{1}H NMR spectrum (DMSO-d6, 600 MHz) of compound **3a**.

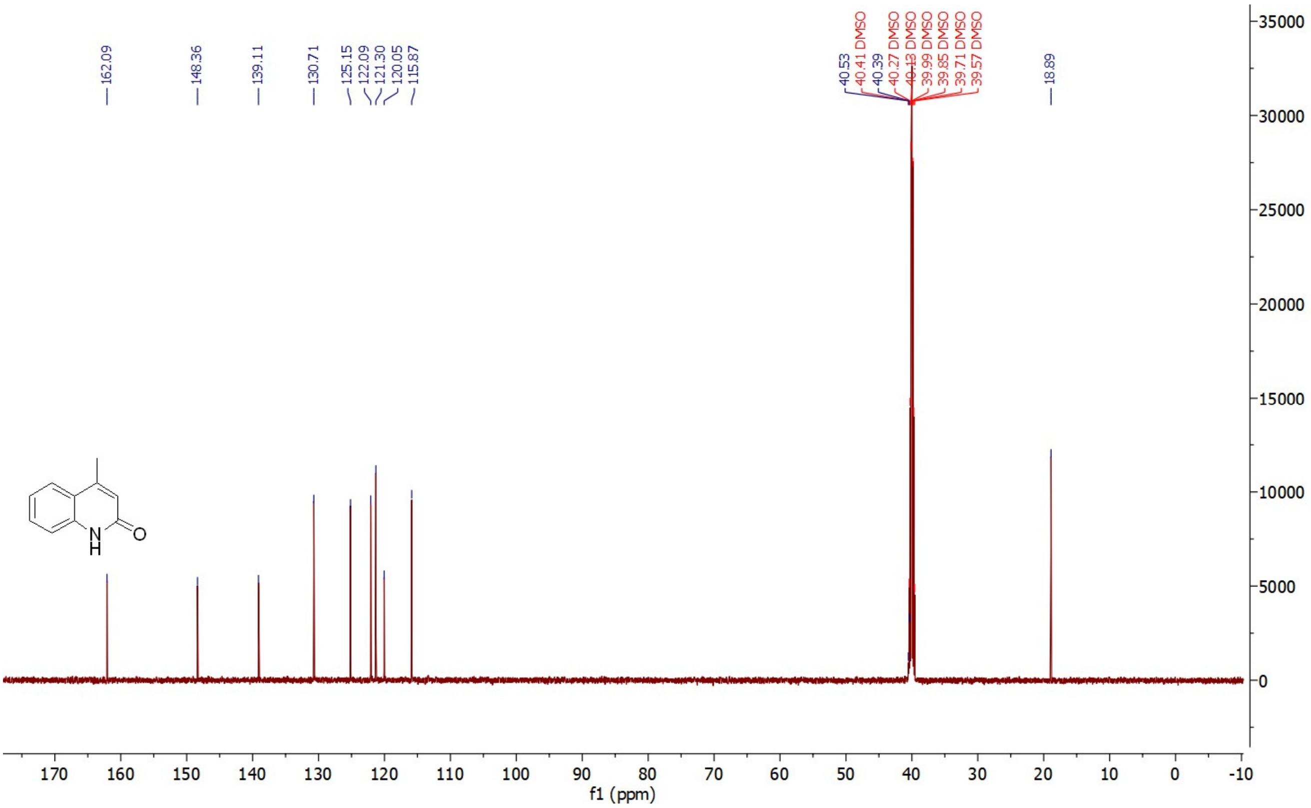

Figure S4. ^{13}C NMR spectrum (DMSO-d6, 151 MHz) of compound **3a**.

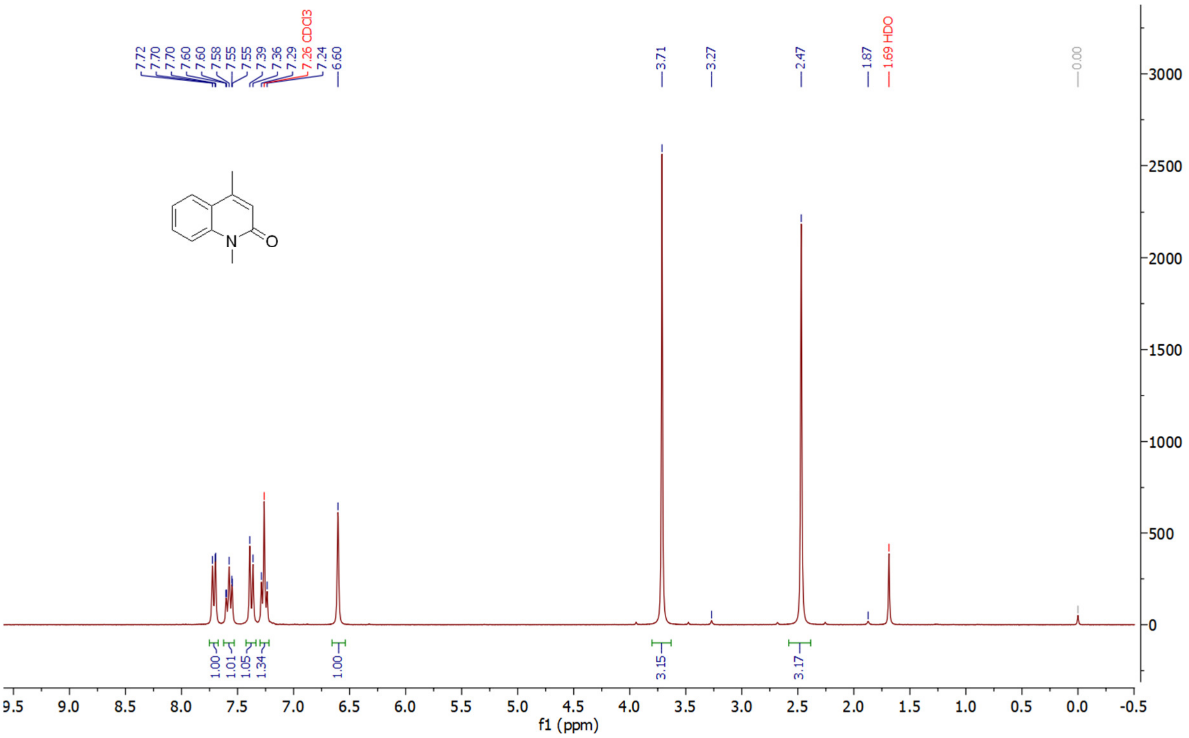

Figure S5. ^{1}H NMR spectrum ($CDCl_3$, 300 MHz) of compound **3b**.

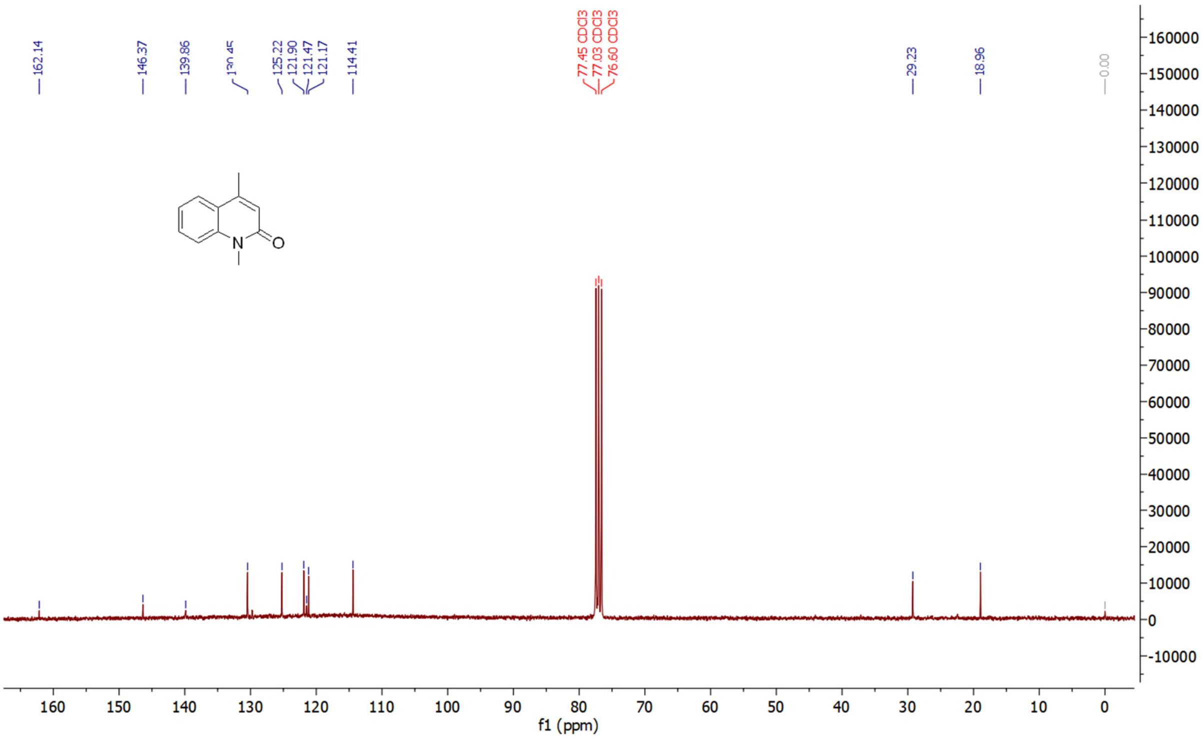

Figure S6. ^{13}C NMR spectrum ($CDCl_3$, 75 MHz) of compound **3b**.

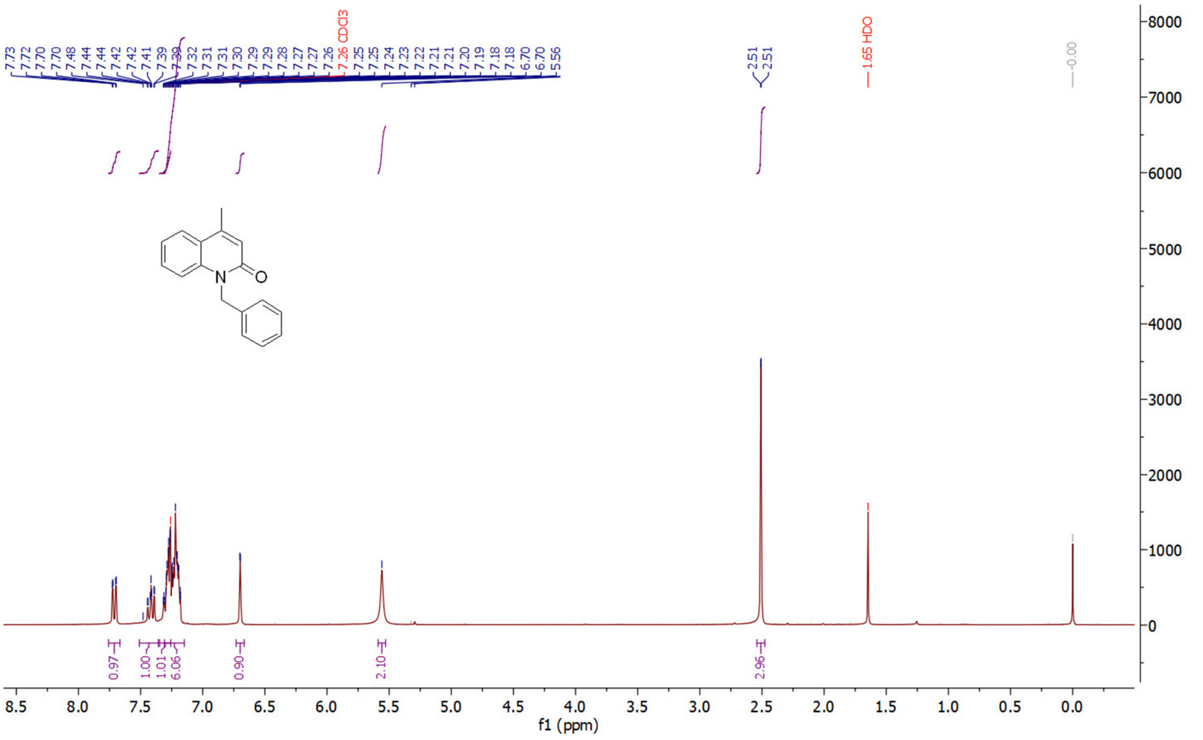

Figure S7. ^{1}H NMR spectrum ($CDCl_3$, 300 MHz) of compound **3c**.

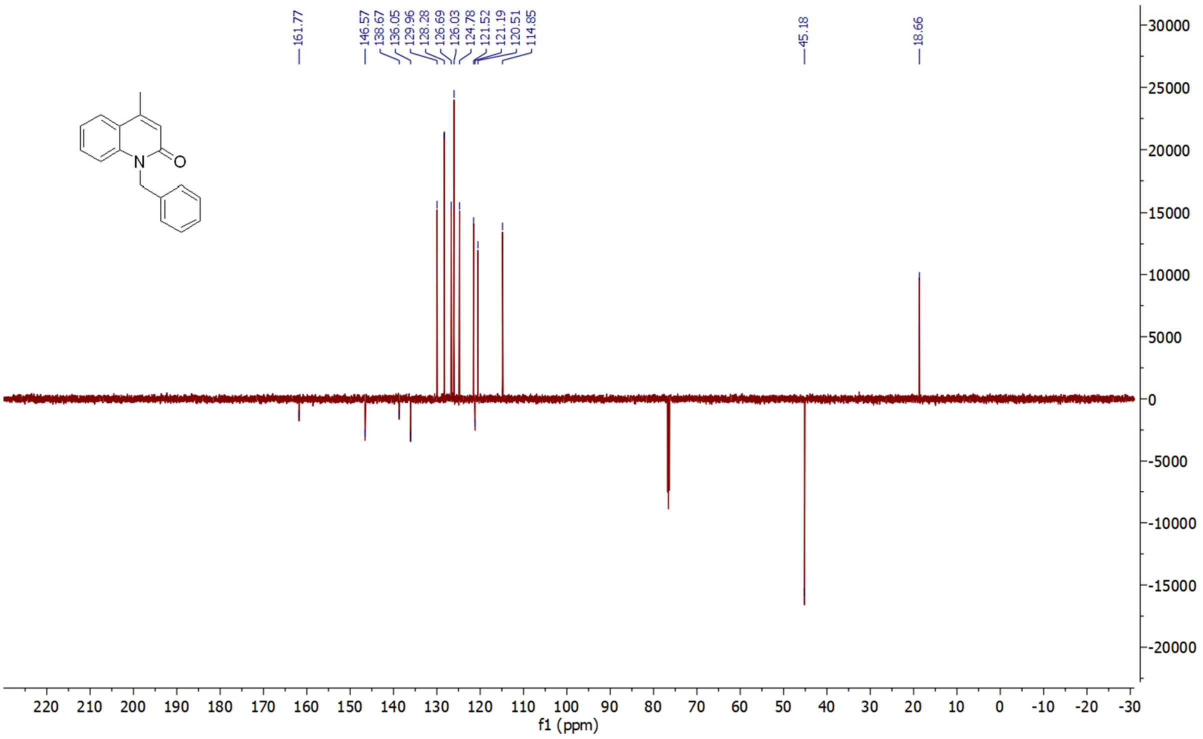

Figure S8. ^{13}C NMR spectrum ($CDCl_3$, 151 MHz) of compound **3c**.

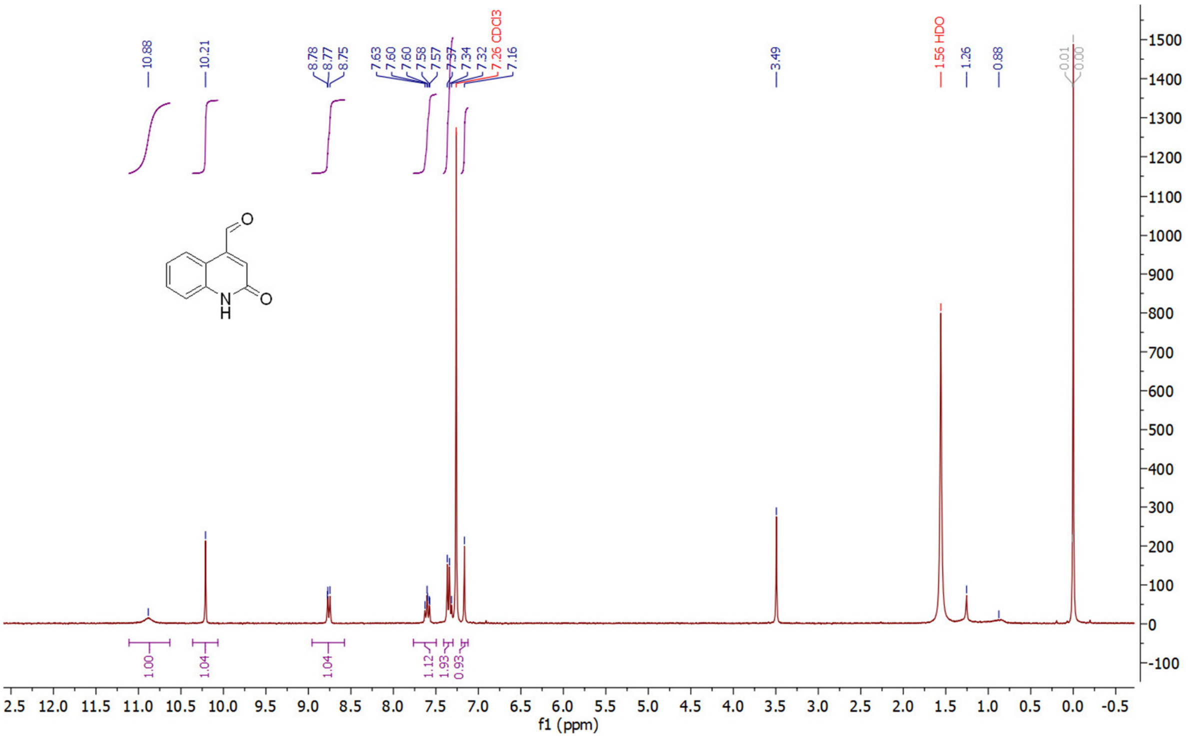

Figure S9. ^{1}H NMR spectrum ($CDCl_3$, 300 MHz) of compound **4a**.

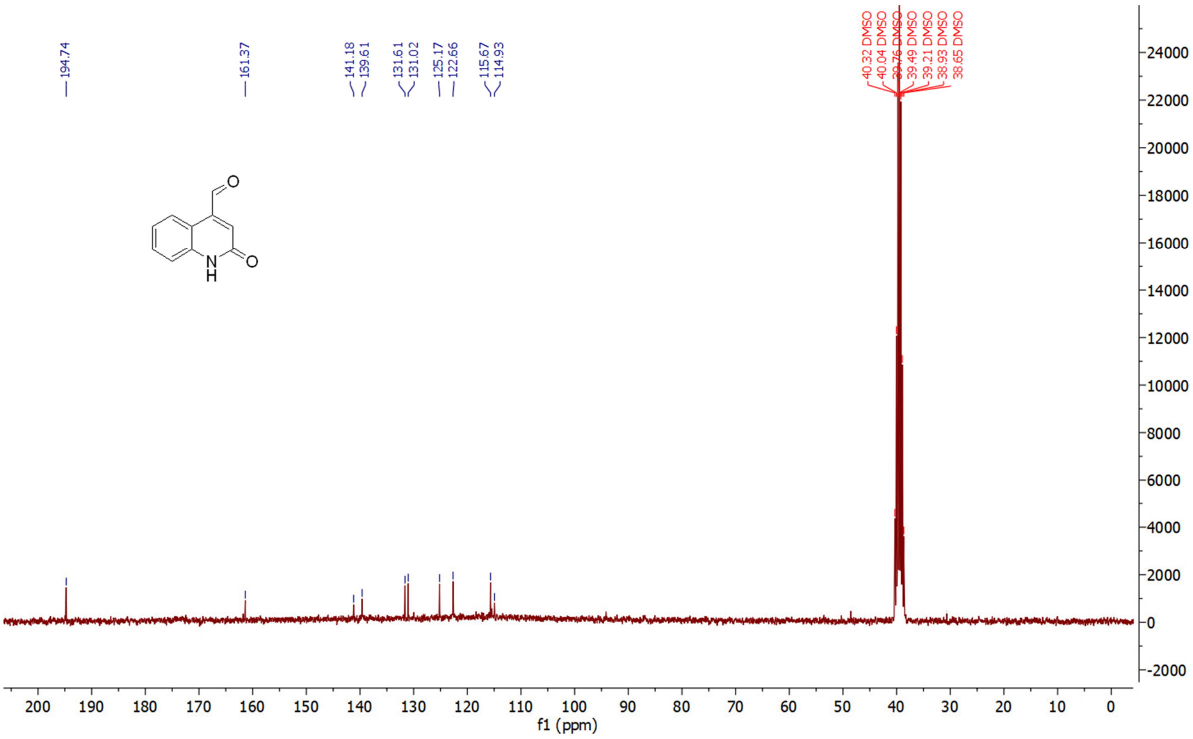

Figure S10. ^{13}C NMR spectrum (DMSO-d_6, 75 MHz) of compound **4a**.

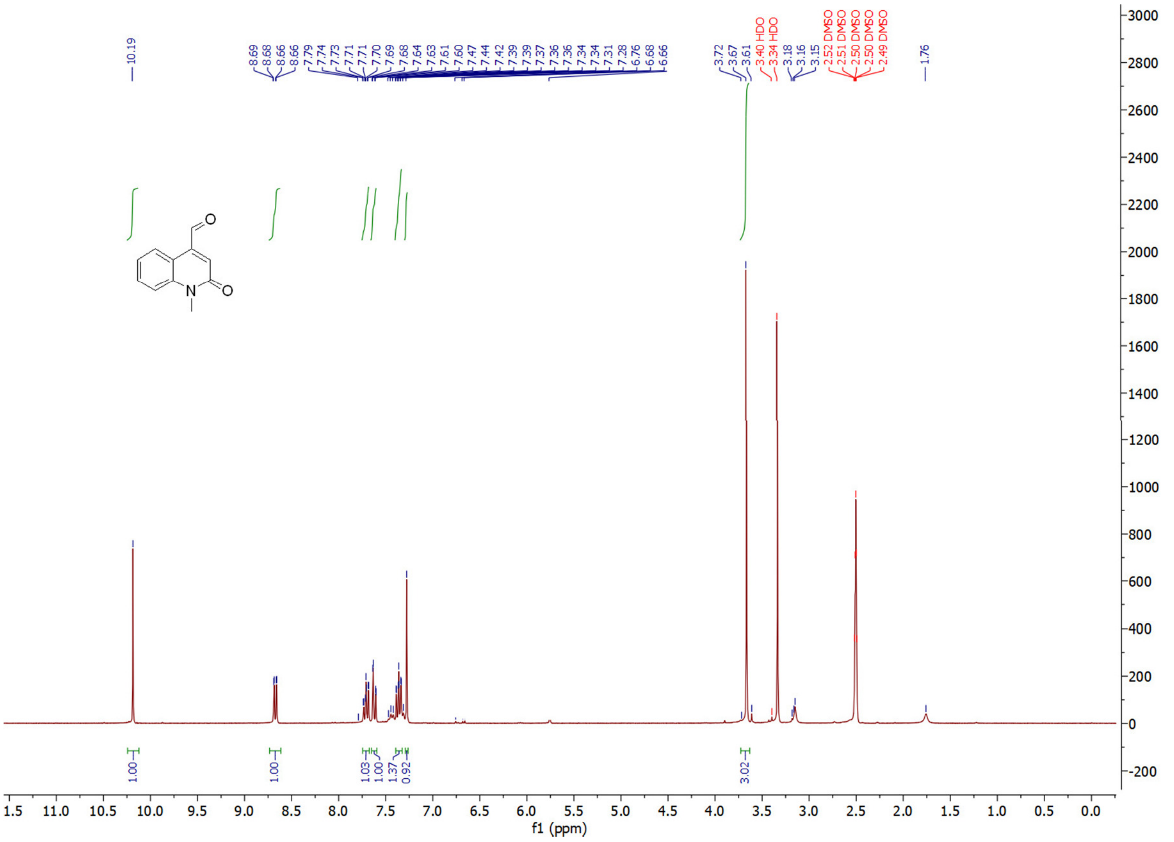

Figure S11. ^{1}H NMR spectrum (DMSO-d_6, 300 MHz) of compound **4b**.

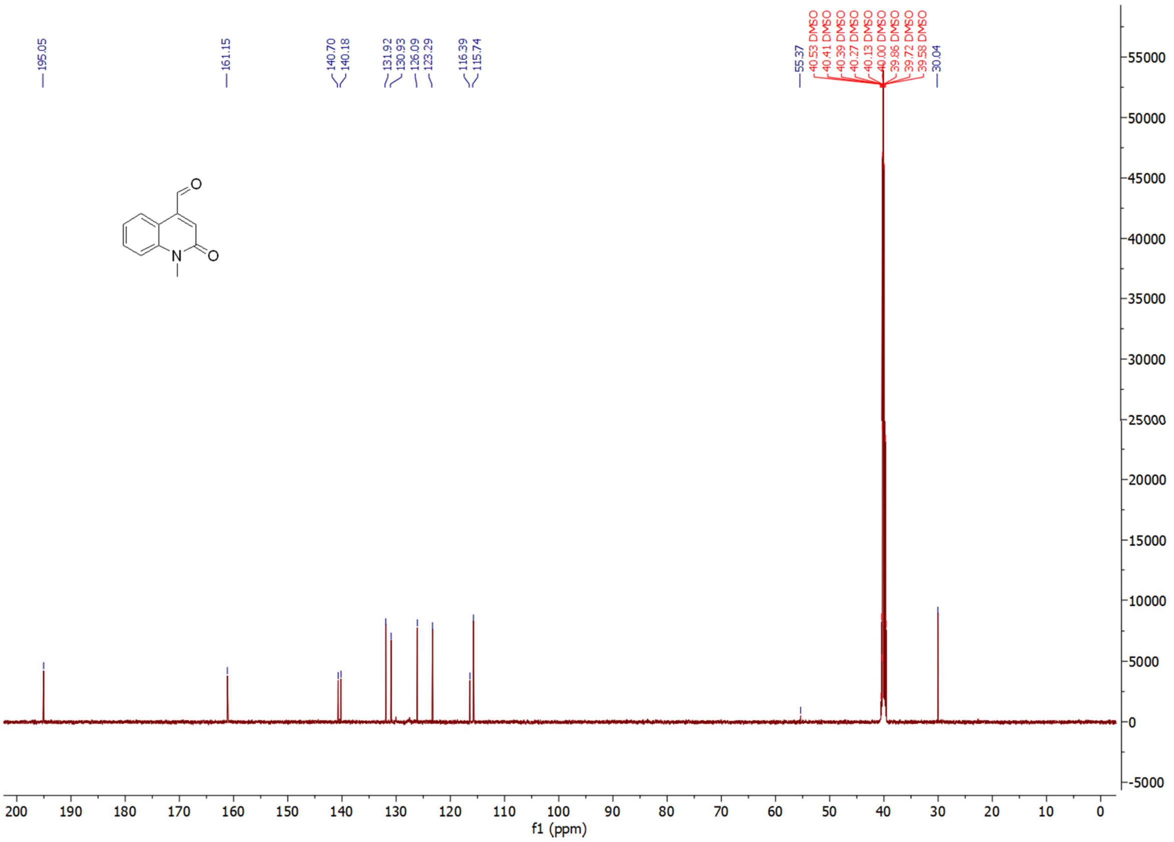

Figure S12. ^{13}C NMR spectrum (DMSO-d_6, 151 MHz) of compound **4b**.

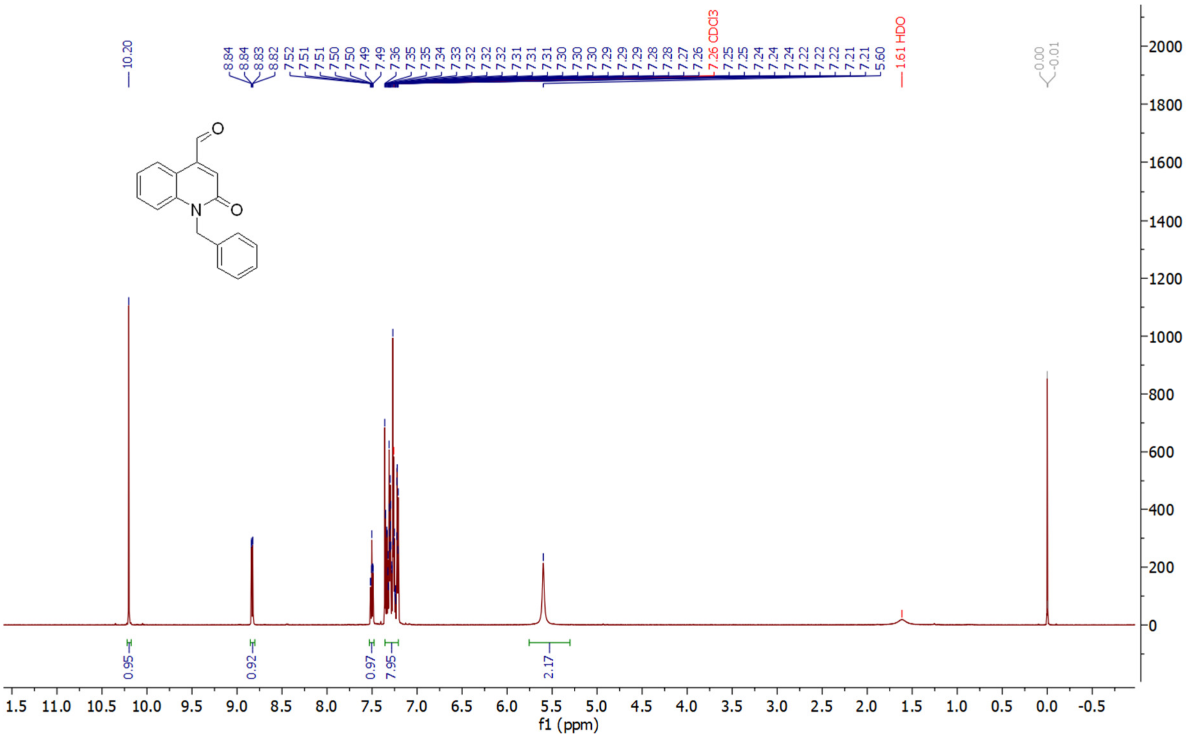

Figure S13. ^{1}H NMR spectrum ($CDCl_3$, 300 MHz) of compound **4c**.

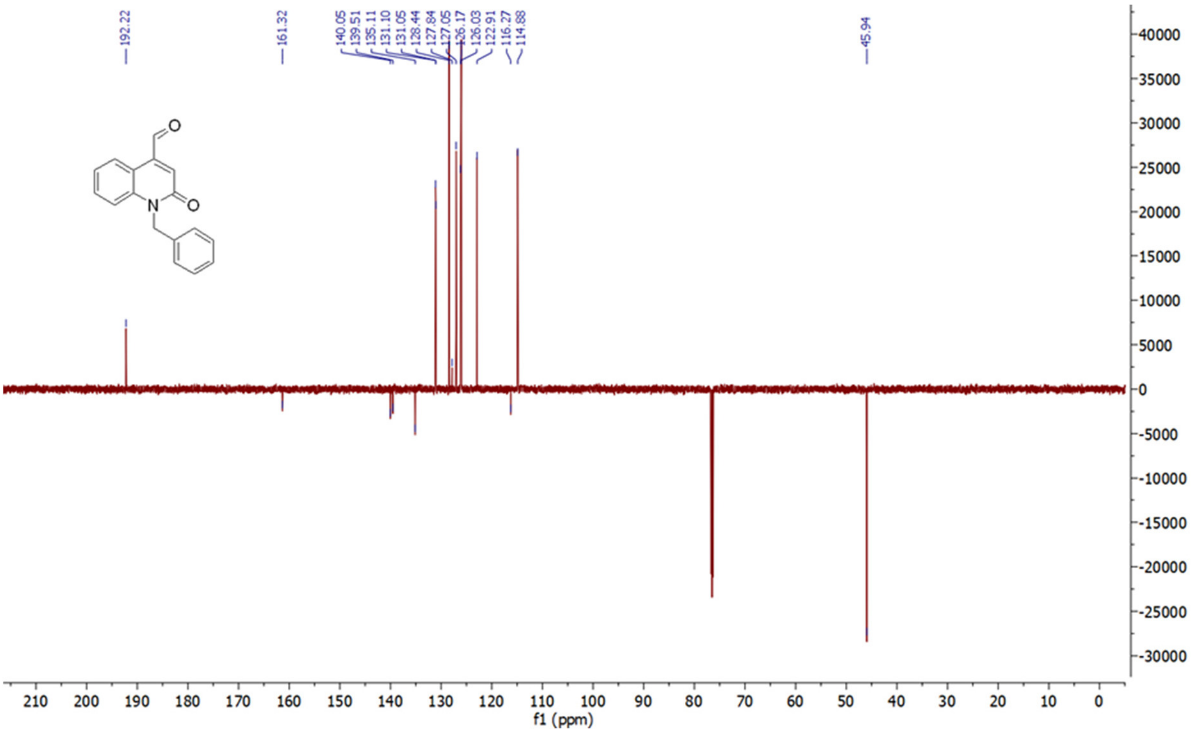

Figure S14. ^{13}C NMR spectrum ($CDCl_3$, 151 MHz) of compound **4c**.

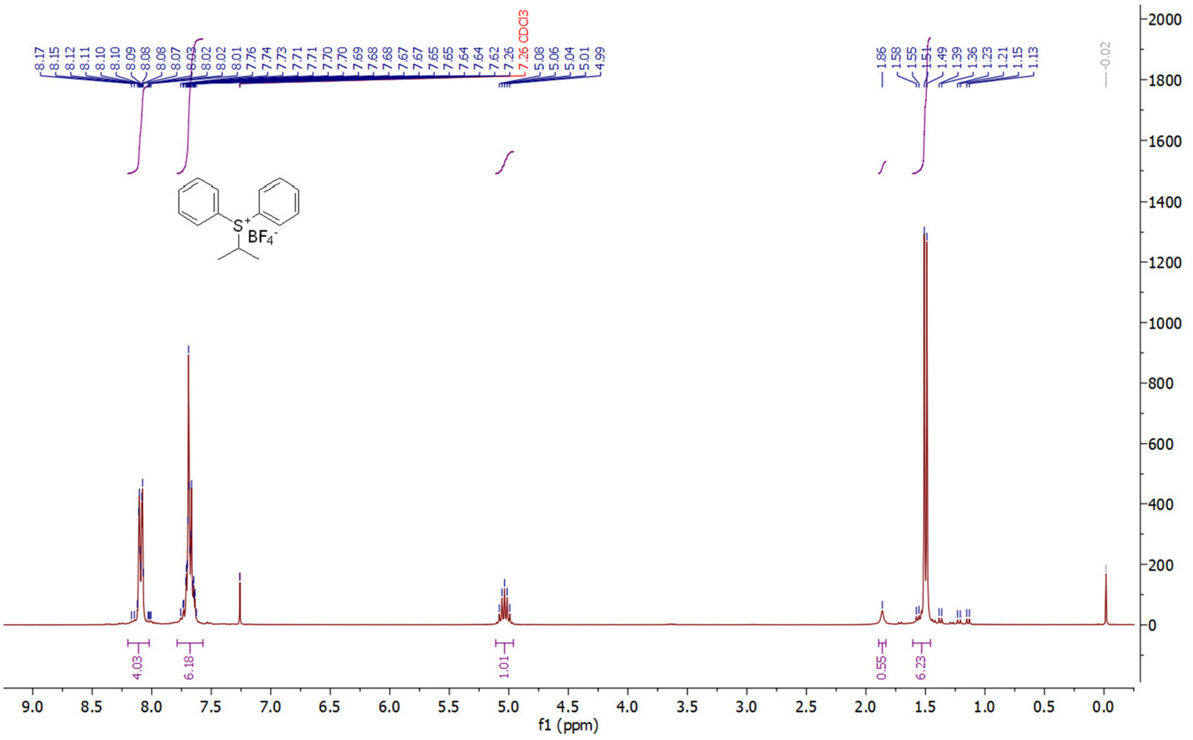

Figure S15. ^{1}H NMR spectrum ($CDCl_3$, 300 MHz) of compound **5**.

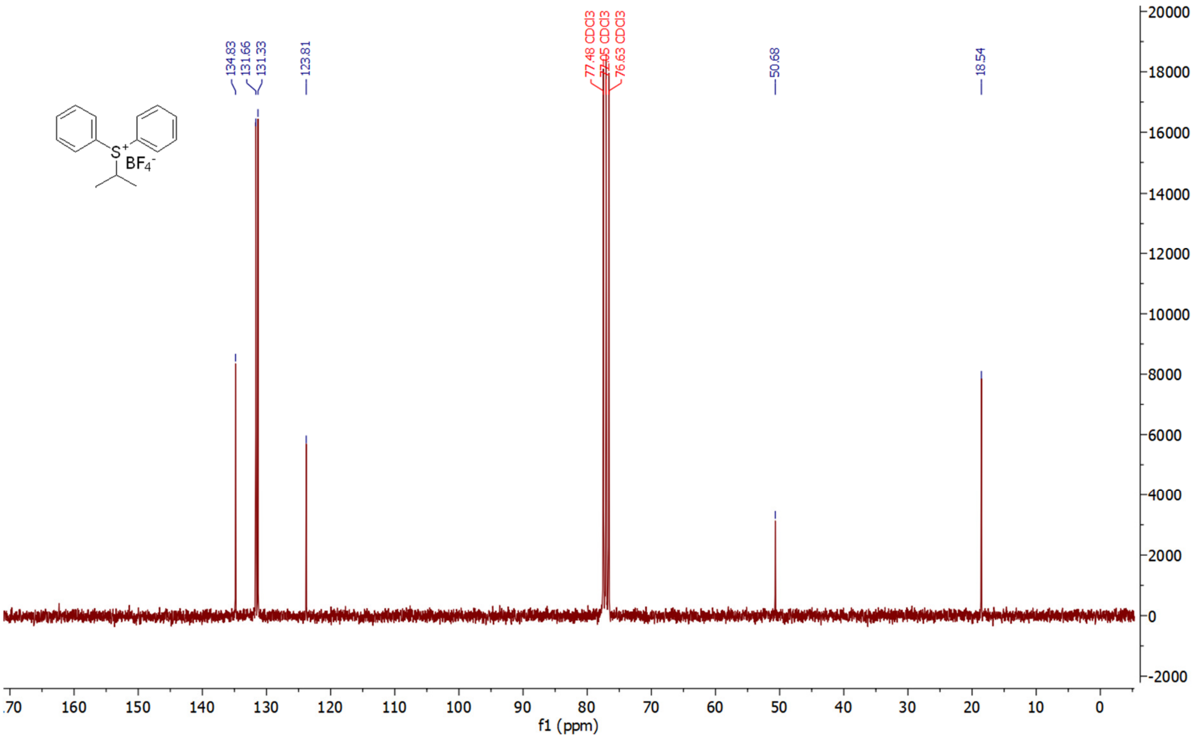

Figure S16. ^{13}C NMR spectrum ($CDCl_3$, 75 MHz) of compound **5**.

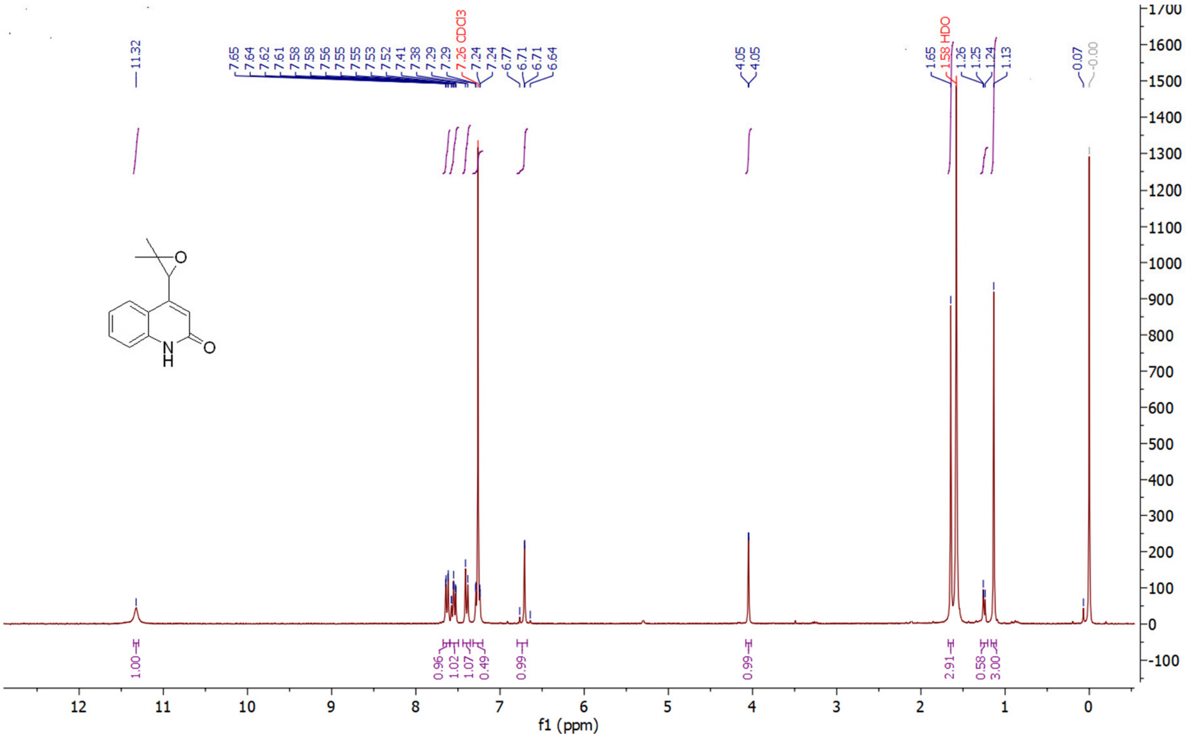

Figure S17. ^{1}H NMR spectrum ($CDCl_3$, 300 MHz) of compound *rac*-**6a.**

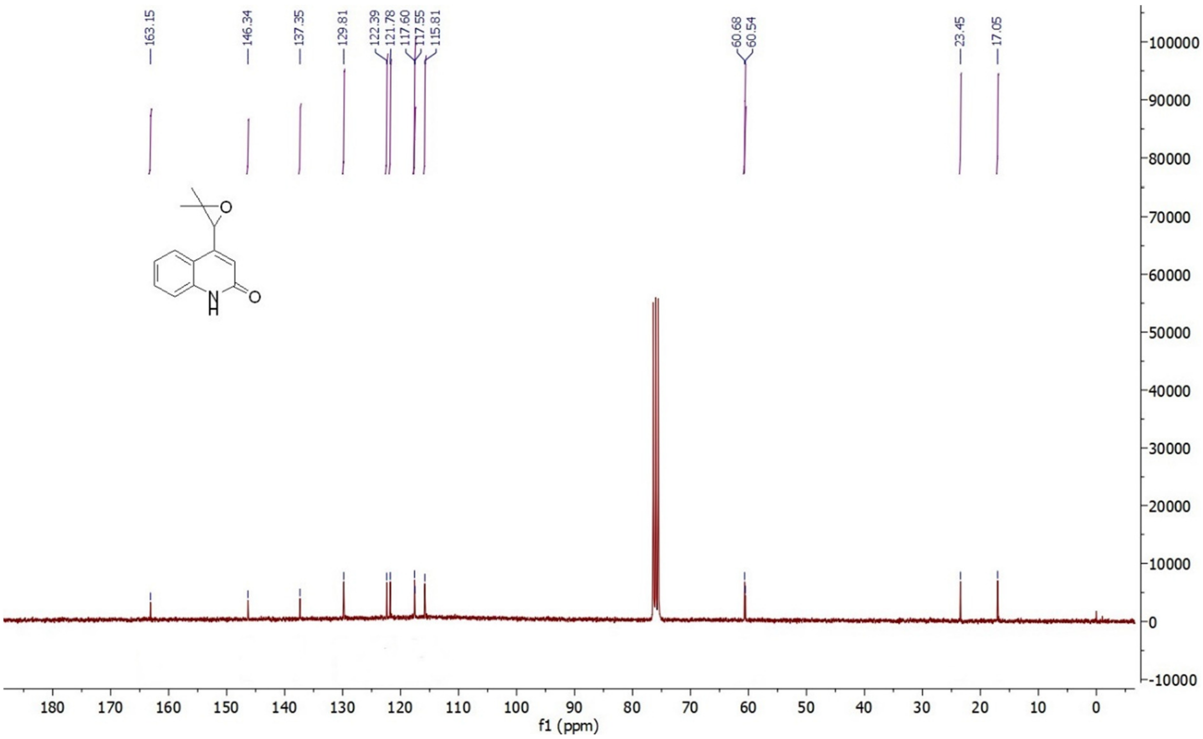

Figure S18. ^{13}C NMR spectrum ($CDCl_3$, 75 MHz) of compound *rac*-**6a.**

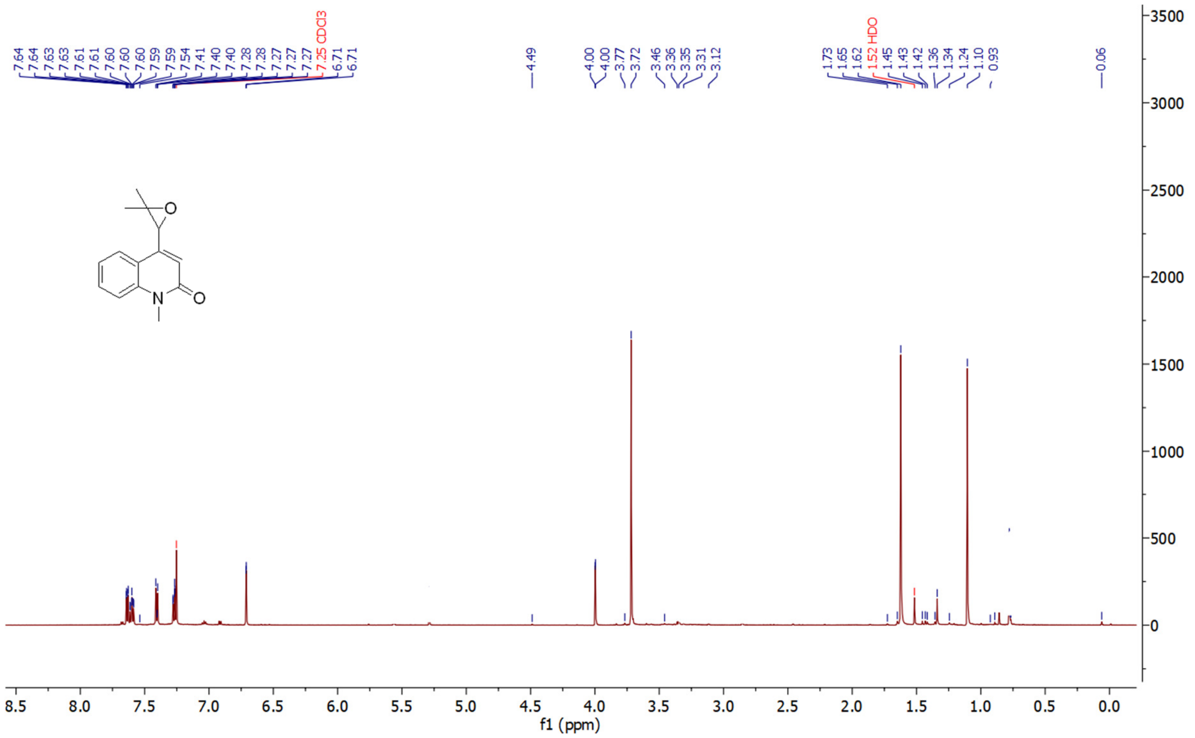

Figure S19. ^{1}H NMR spectrum ($CDCl_3$, 600 MHz) of compound *rac*-**6b**.

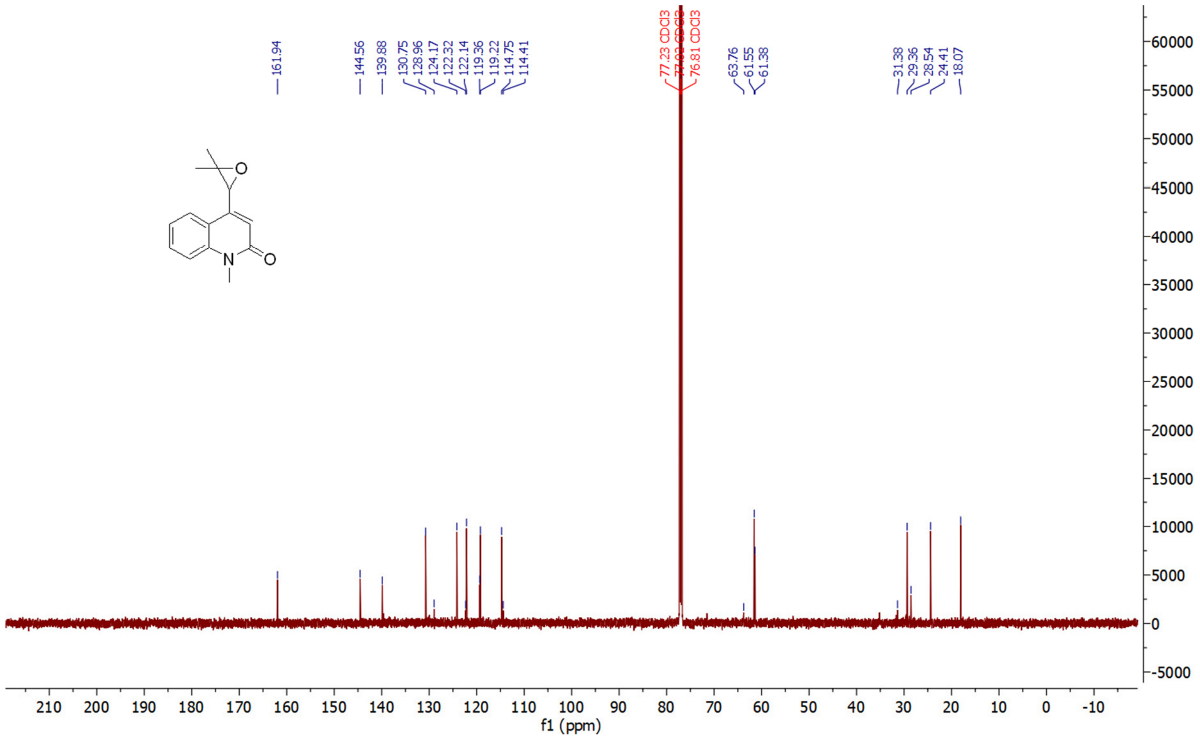

Figure S20. ^{13}C NMR spectrum ($CDCl_3$, 151 MHz) of compound *rac*-**6b.**

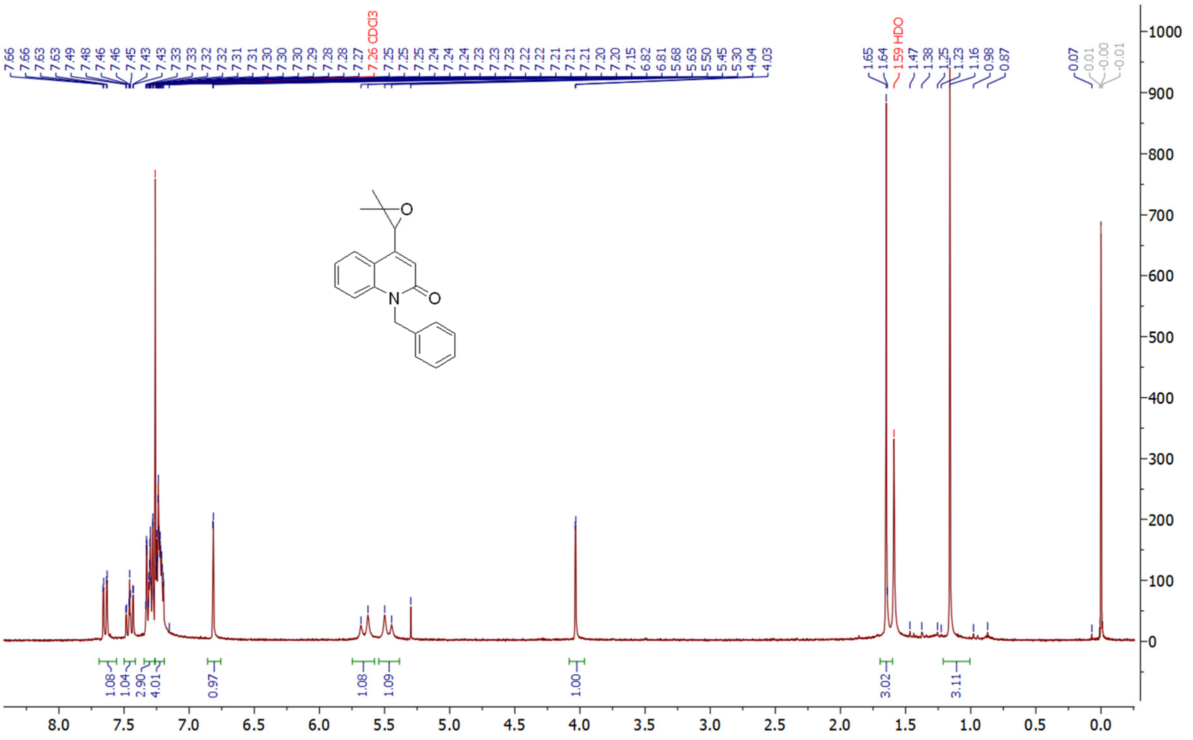

Figure S21. ^{1}H NMR spectrum ($CDCl_3$, 300 MHz) of compound *rac*-**6c.**

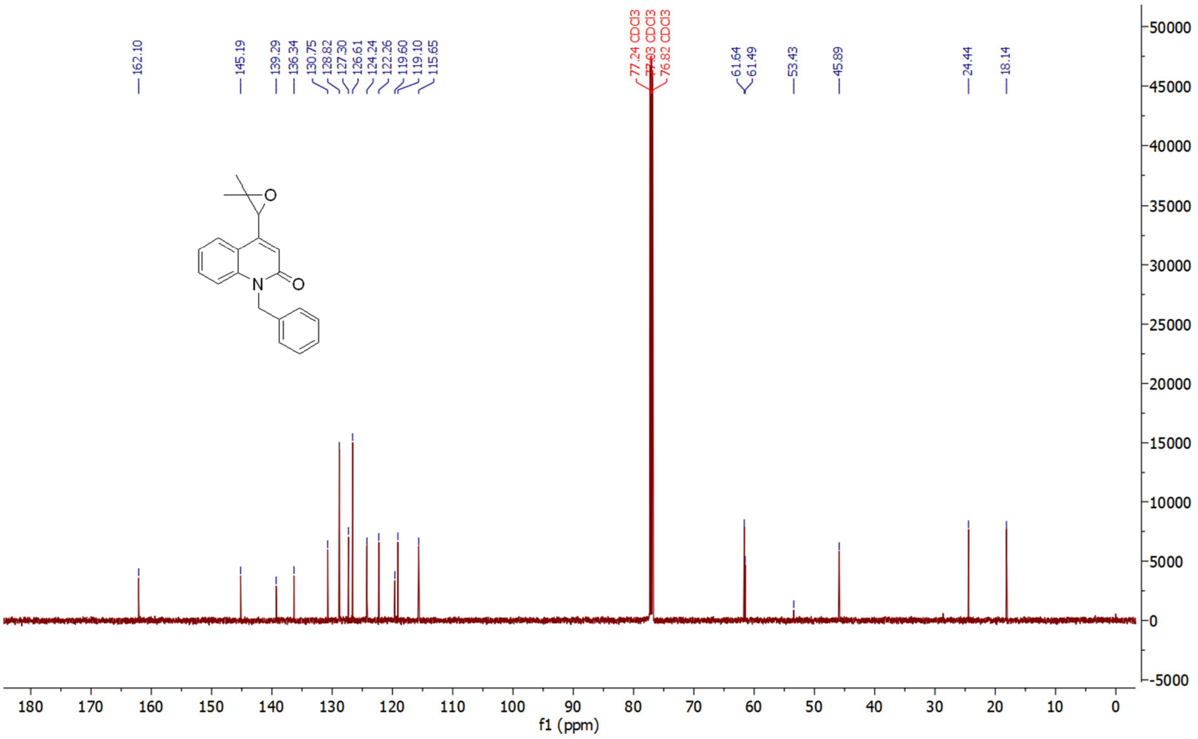

Figure S22. ^{13}C NMR spectrum ($CDCl_3$, 75 MHz) of compound *rac*-**6c.**

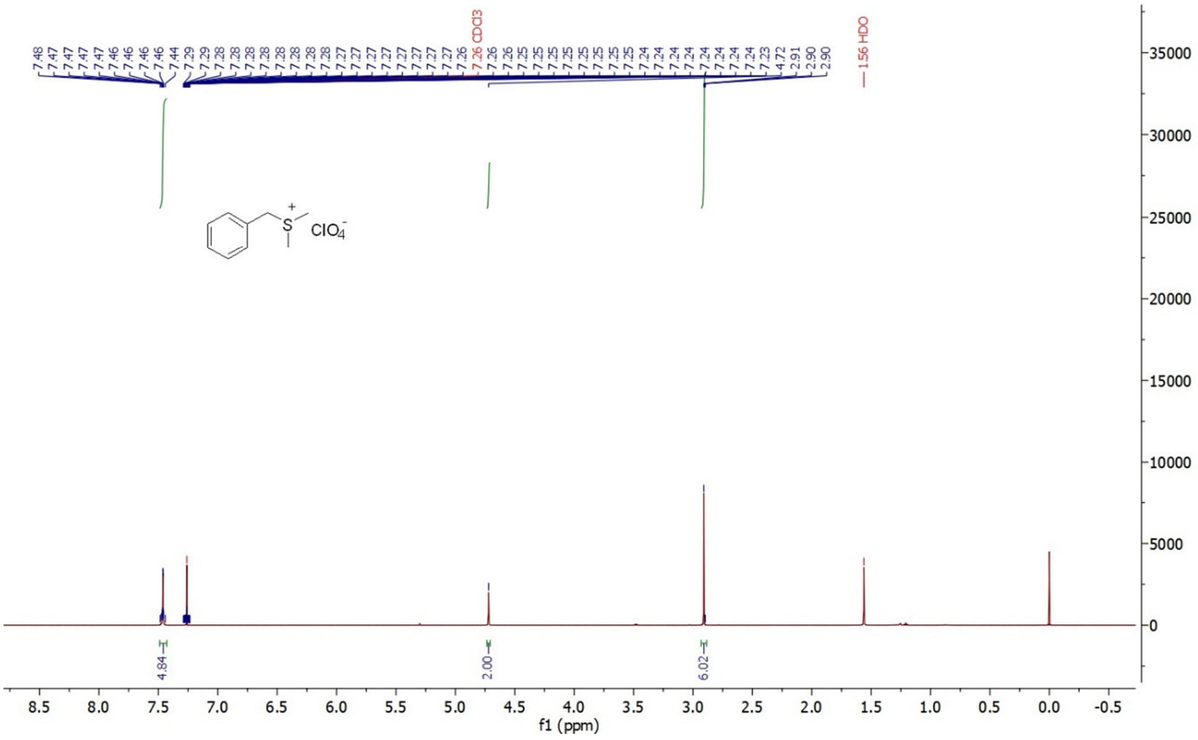

Figure S23. ^{1}H NMR spectrum ($CDCl_3$, 300 MHz) of compound **7**.

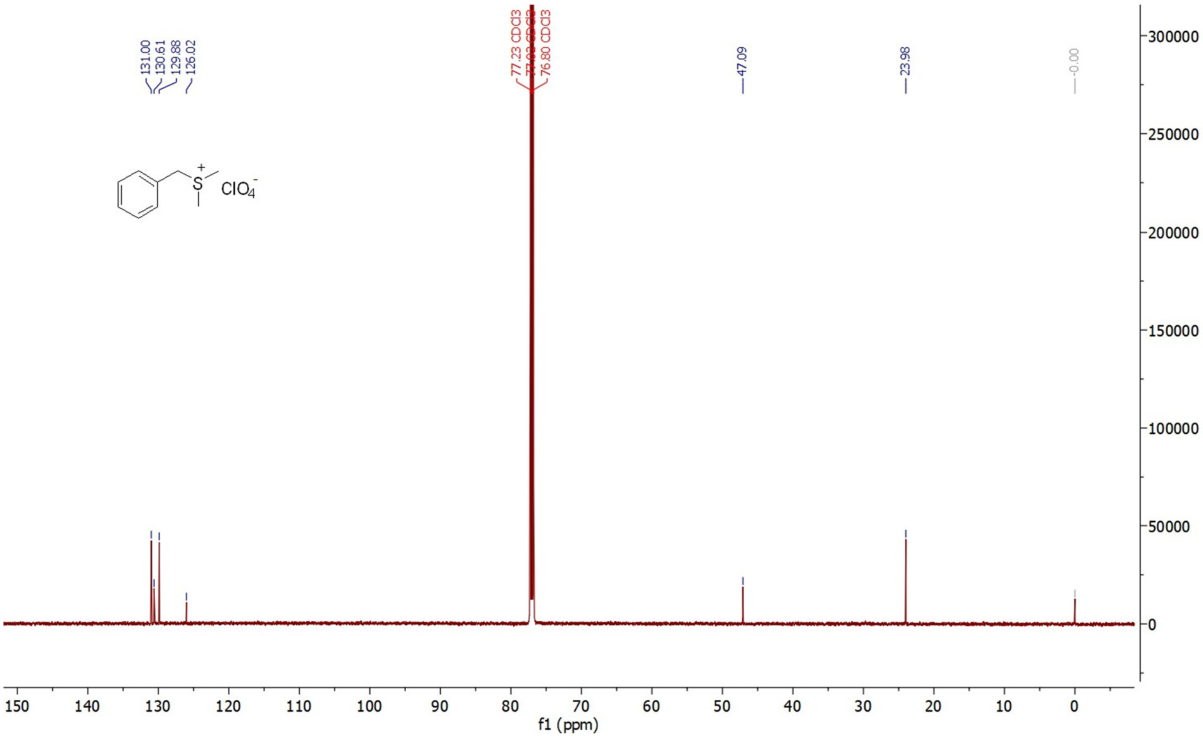

Figure S24. ^{13}C NMR spectrum ($CDCl_3$, 75 MHz) of compound **7**.

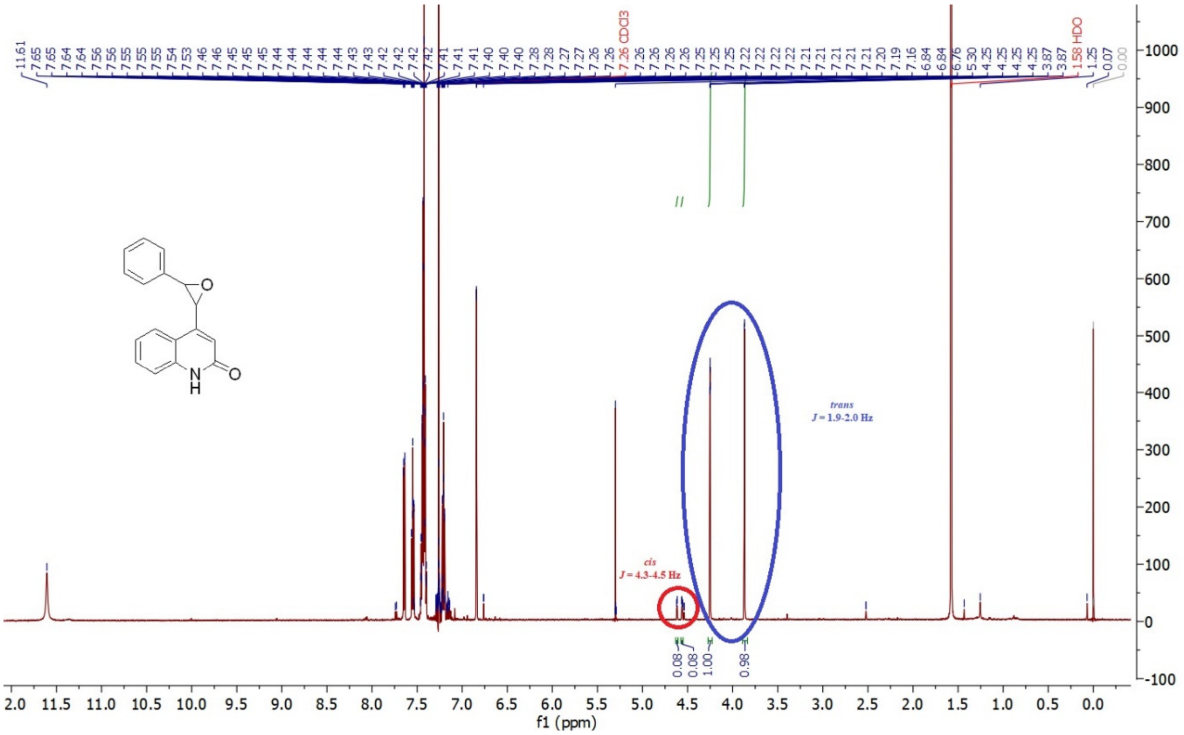

Figure S25. ^{1}H NMR ($CDCl_3$, 600MHz) of diastereomeric mixture of compound *rac*-**8a**.

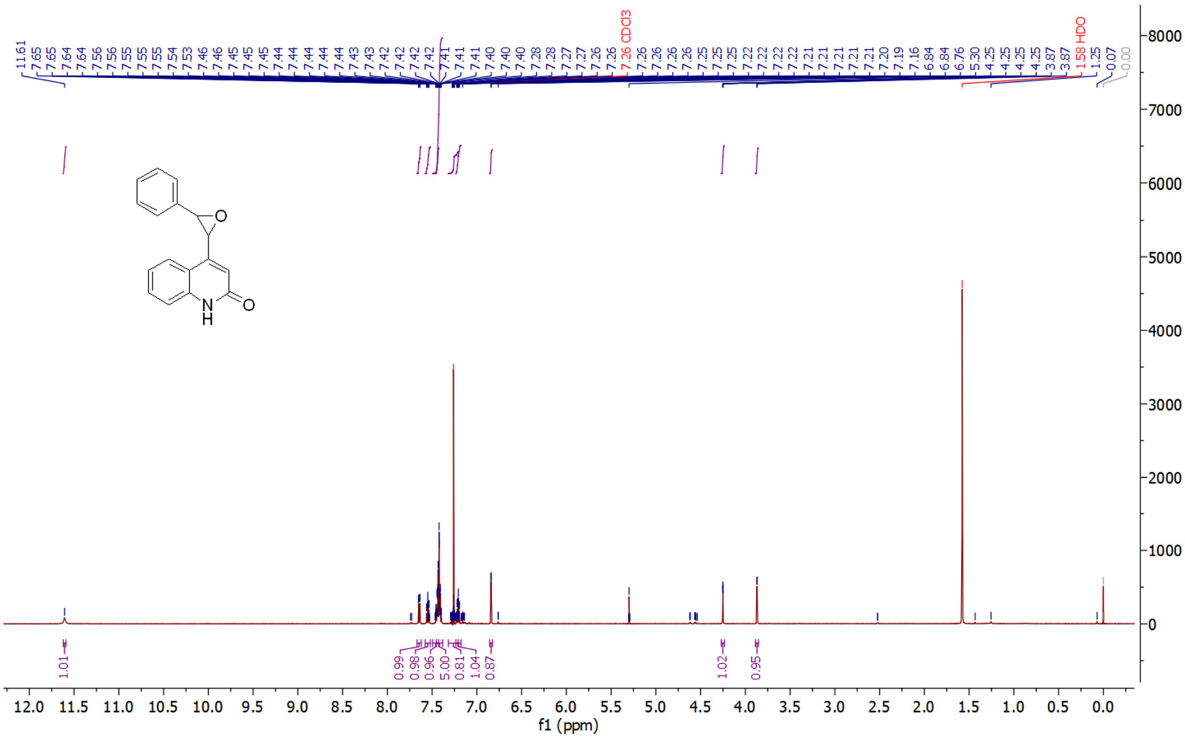

Figure S26. ^{1}H NMR spectrum ($CDCl_3$, 600 MHz) of the major diastereomer of compound *rac*-**8a**.

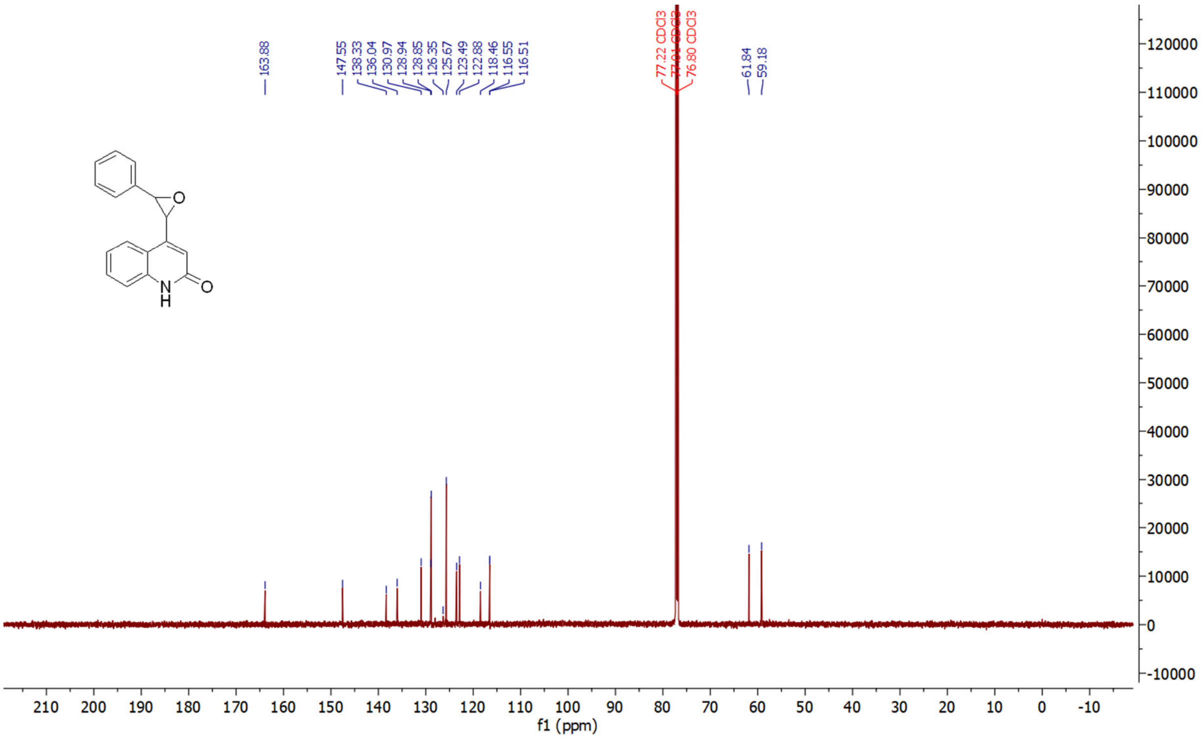

Figure S27. ^{13}C NMR spectrum ($CDCl_3$, 151 MHz) of the major diastereomer of compound *rac*-**8a.**

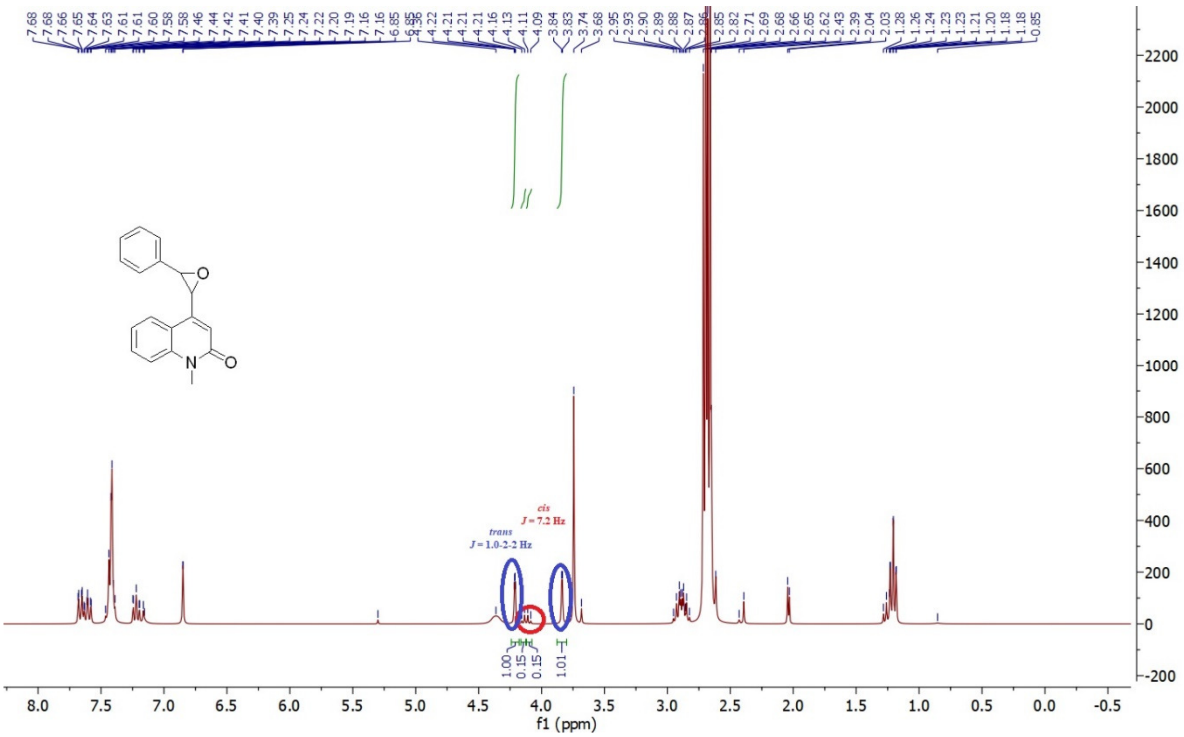

Figure S28. ¹H NMR ($CDCl_3$, 300MHz) of diastereomeric mixture of compound *rac*-**8b**.

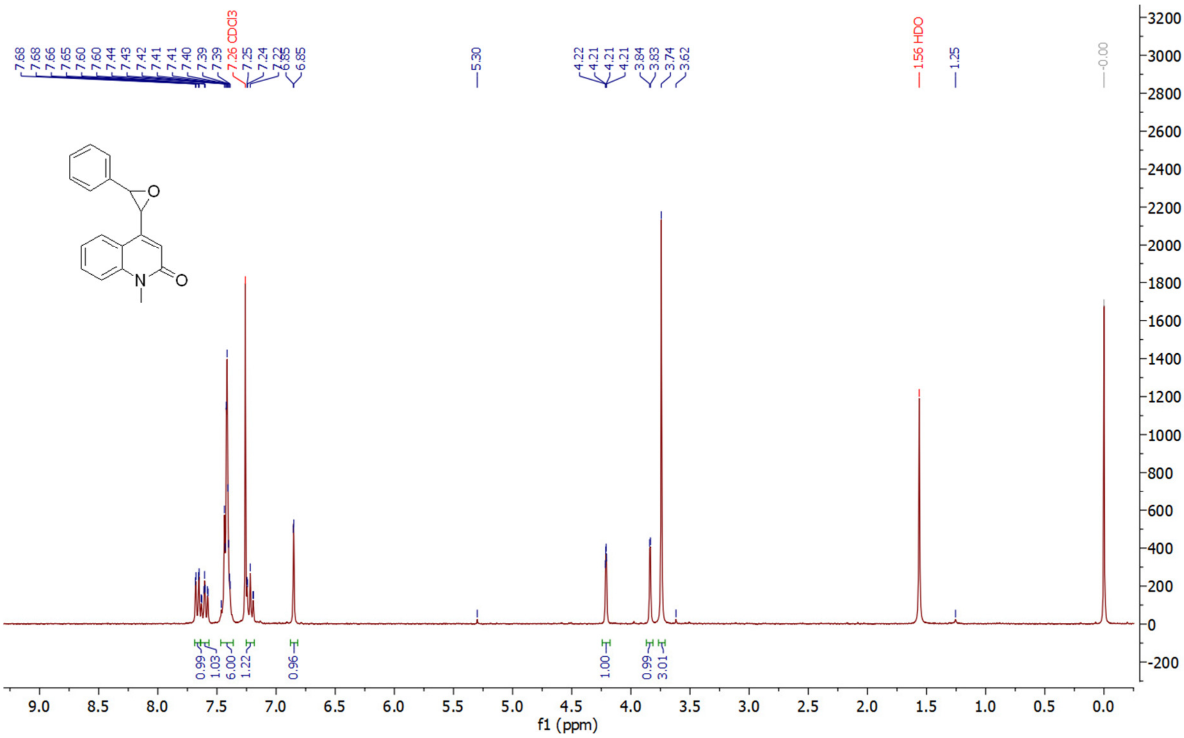

Figure S29. ¹H NMR spectrum ($CDCl_3$, 300 MHz) of the major diastereomer of compound *rac*-**8b.**

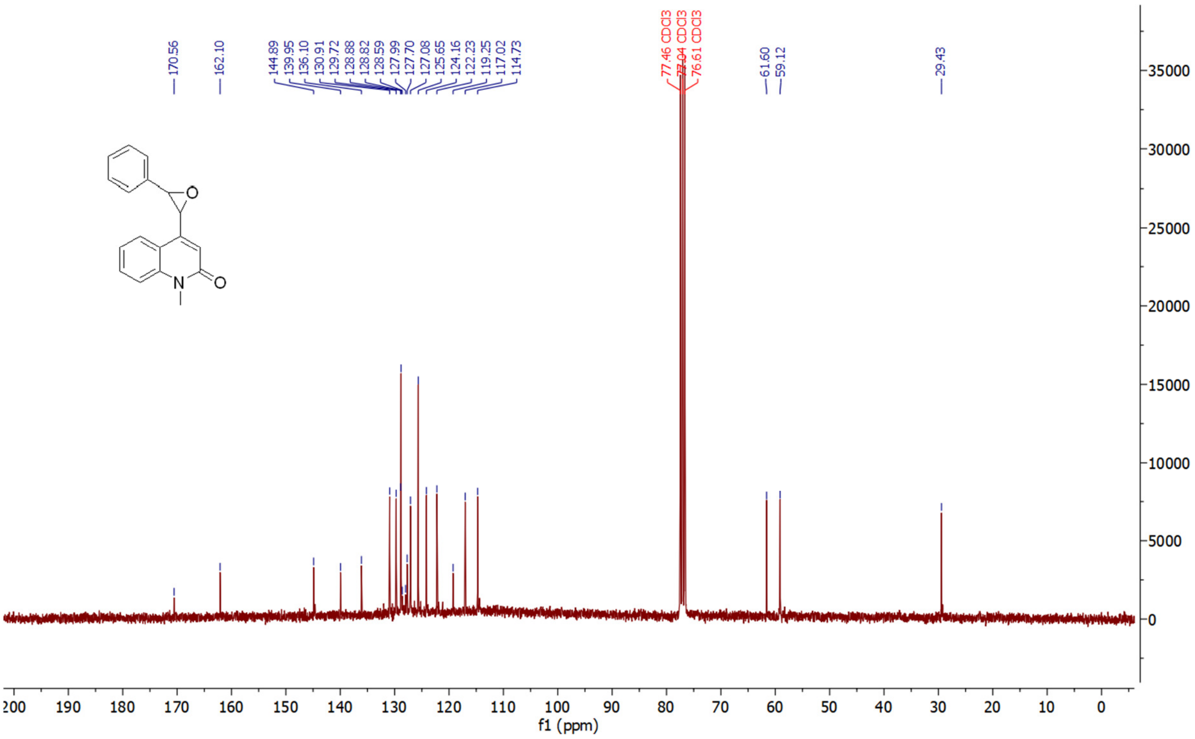

Figure S30. ^{13}C NMR spectrum ($CDCl_3$, 75 MHz) of the major diastereomer of compound *rac*-**8b.**

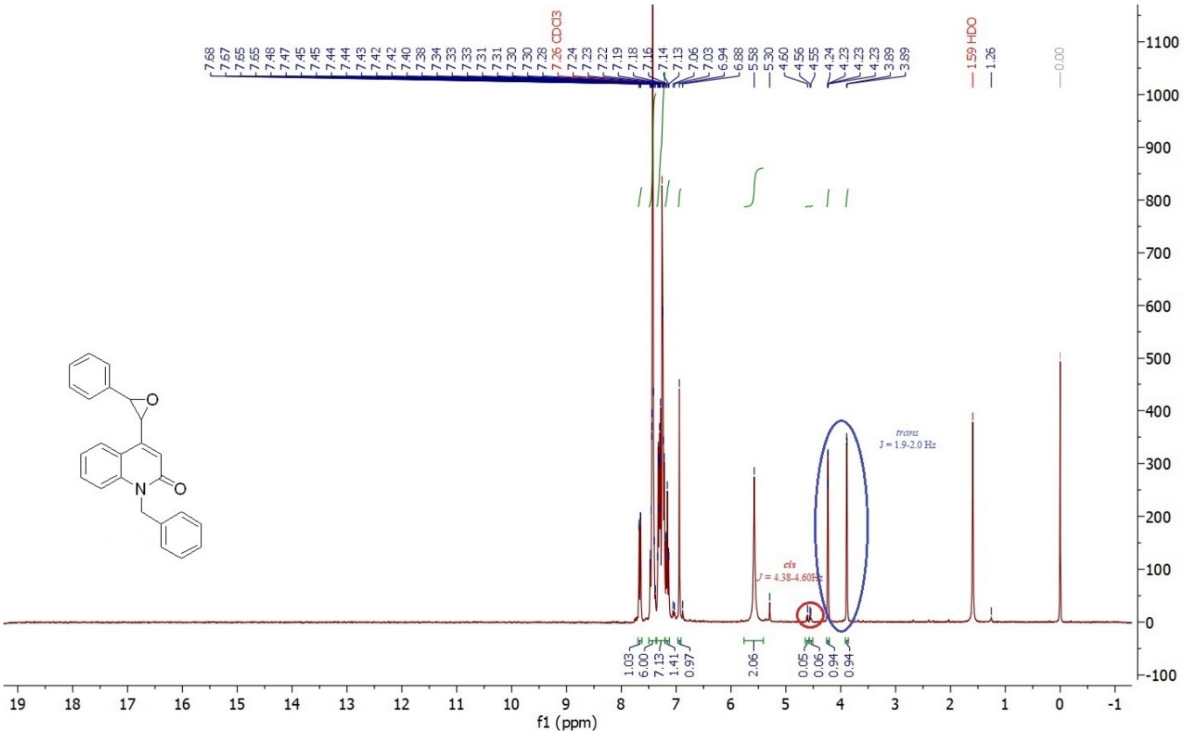

Figure S31. ^{1}H NMR ($CDCl_3$, 300MHz) of diastereomeric mixture of compounds *rac*-**8c**.

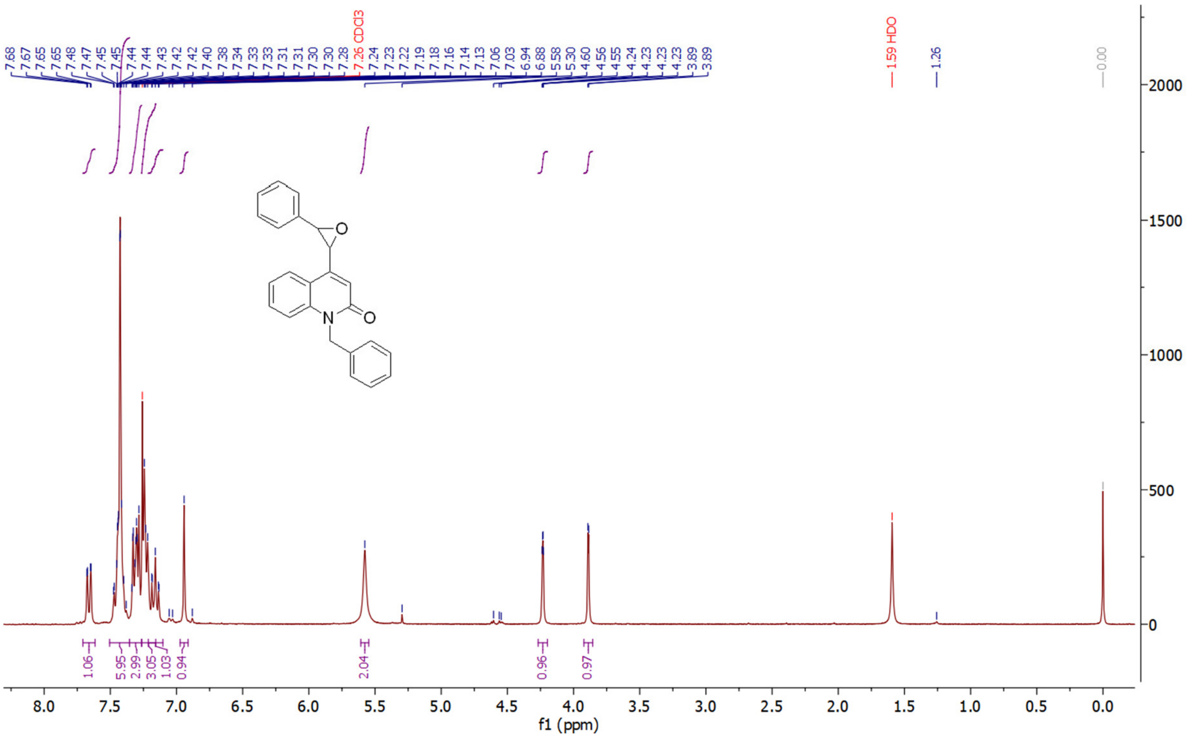

Figure S32. ^{1}H NMR spectrum ($CDCl_3$, 300 MHz) of compound *rac*-**8c.**

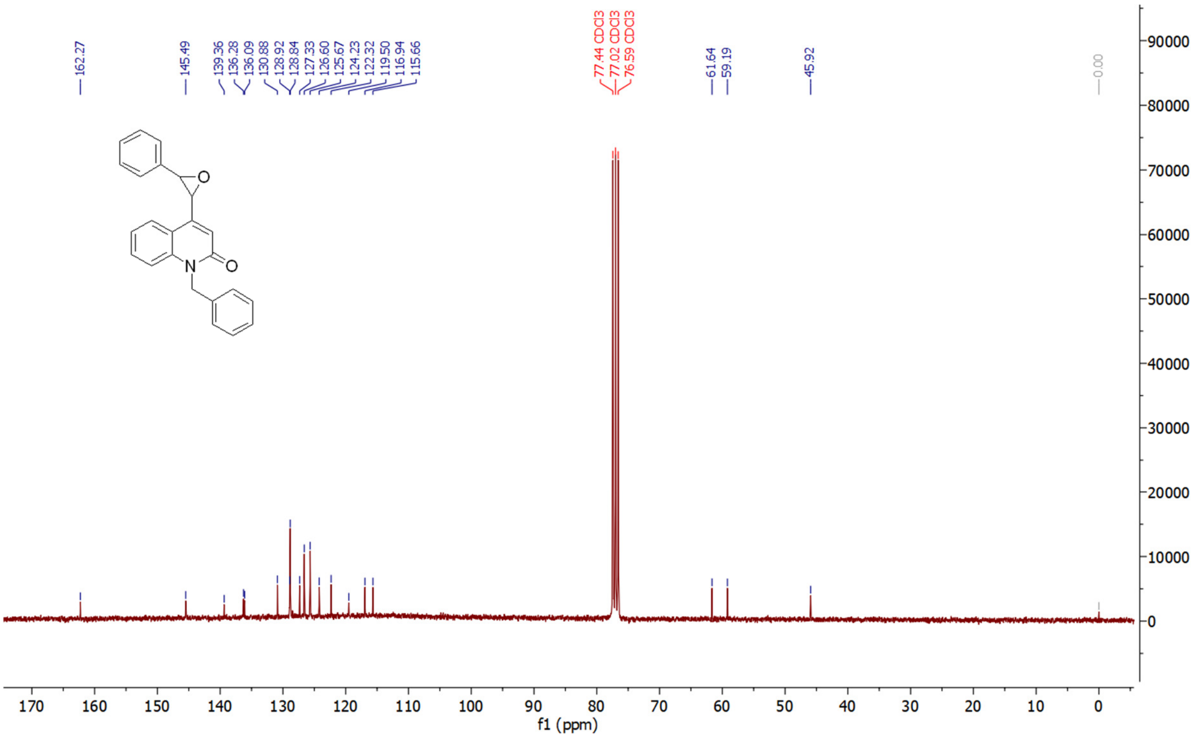

Figure S33. ^{13}C NMR spectrum ($CDCl_3$, 75 MHz) of compound *rac*-**8c.**

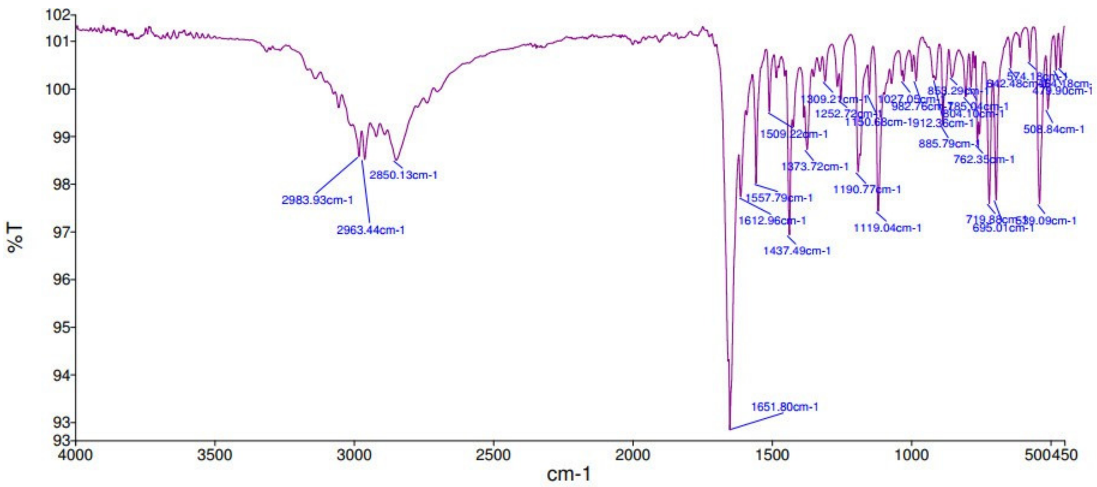

Figure S34. IR spectrum of compound *rac*-**6a**.

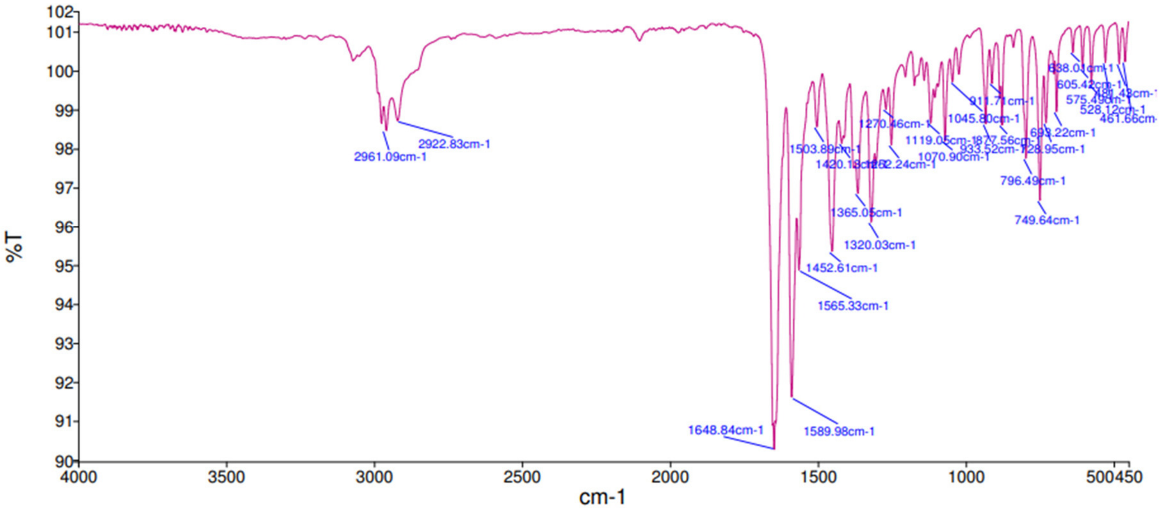

Figure S35. IR spectrum of compound *rac*-**6b.**

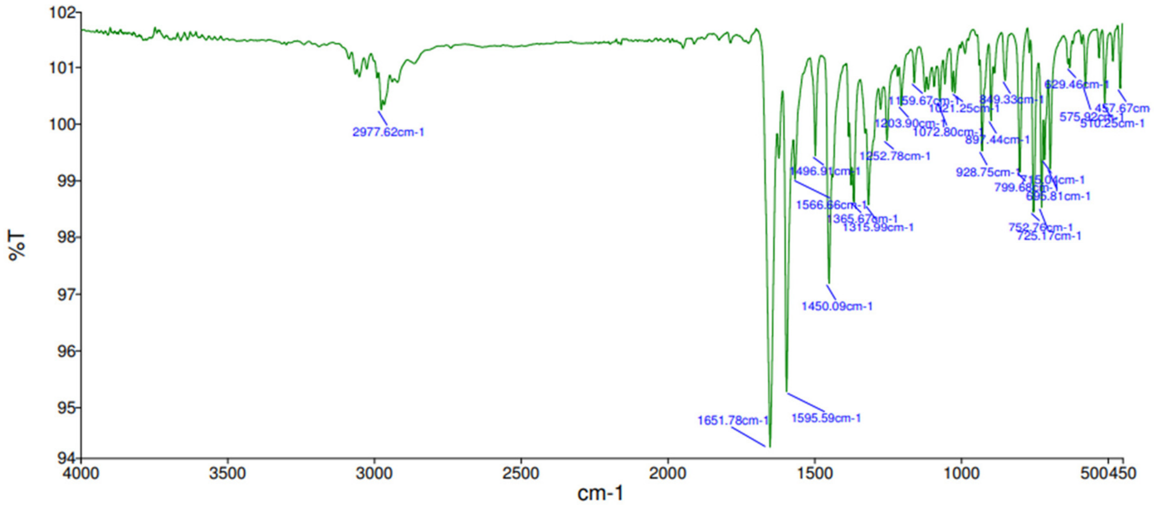

Figure 36. IR spectrum of compound *rac*-**6c.**

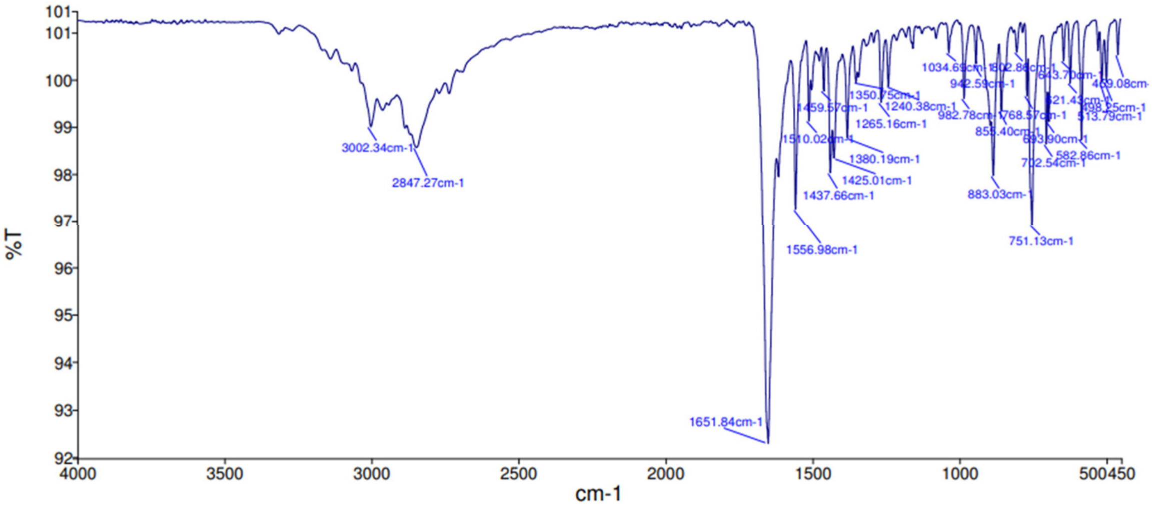

Figure S37. IR spectrum of compound *rac*-**8a.**

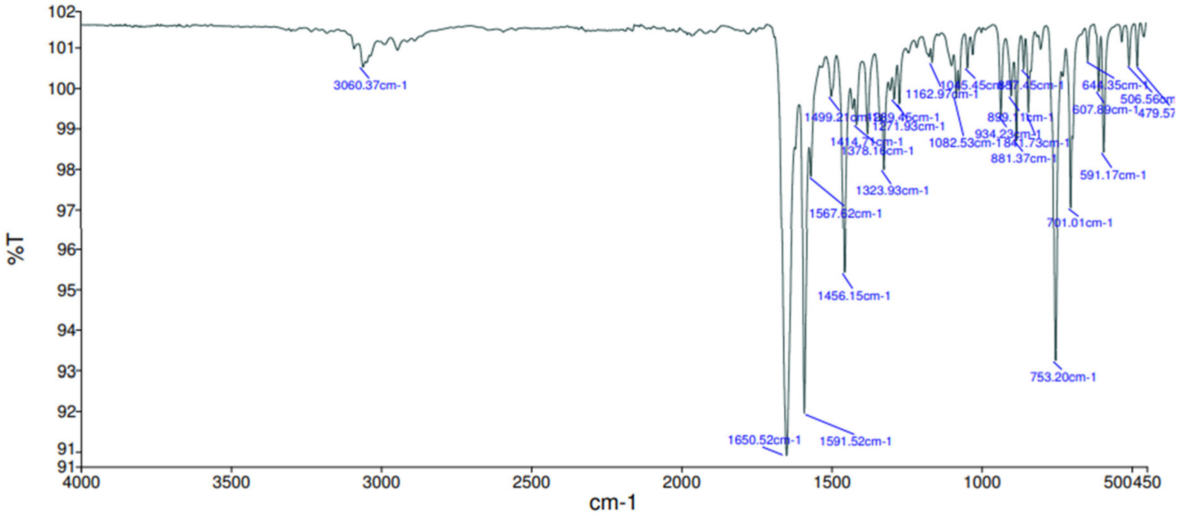

Figure 38. IR spectrum of compound *rac*-**8b.**

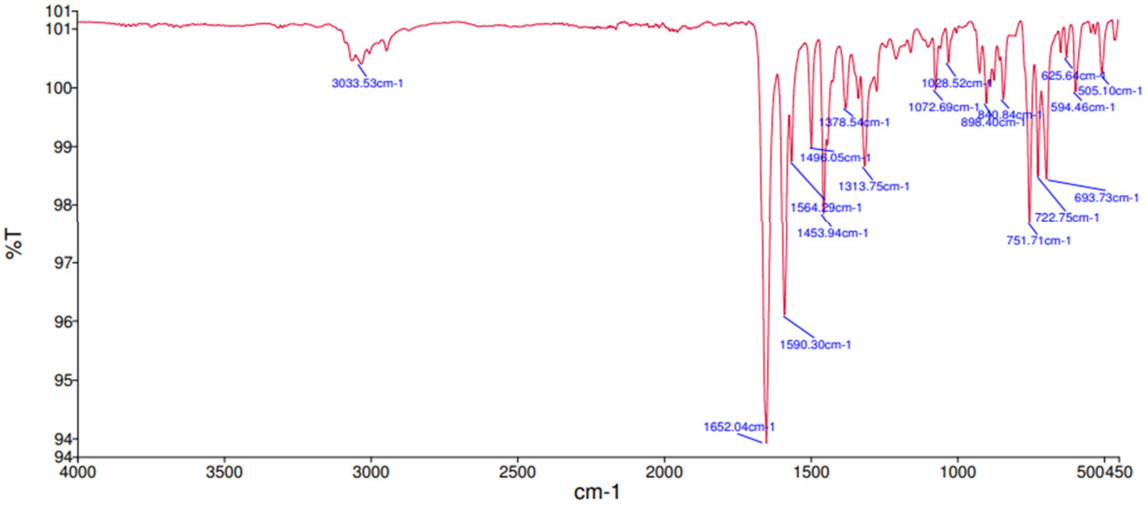

Figure S39. IR spectrum of compound *rac*-**8c**

Table S1. Chromatographic parameters for HPLC and SFC enantioseparation of *rac*-**6a-c**.

Marinoepoxide	Mobile phase	k_1	k_2	α	R_s	k_1	k_2	α	R_s
rac-**6a**	*n*-hexane/EtOH (80/20, *v*/v)	1.20	2.43	2.02	4.34	2.98	3.89	1.31	2.12
	DMC	1.51	2.24	1.48	1.99	5.82	9.26	1.59	3.31
	DMC/MeOH (90/10, *v*/v)	0.35	0.52	1.49	1.42	1.11	1.71	1.54	2.69
	DMC/EtOH (90/10, *v*/v)	0.51	0.77	1.50	1.72	1.55	2.33	1.50	2.60
	CO_2/MeOH (80/20, *v*/v)	0.98	2.10	2.14	2.63	1.78	2.85	1.60	2.38
	CO_2/MeOH (85/15, *v*/v)	1.63	3.34	2.05	3.25	3.28	5.23	1.60	3.02
	CO_2/MeOH (90/10, *v*/v)	2.93	5.98	2.04	3.98	6.10	9.92	1.63	3.90
	CO_2/MOH (95/5, *v*/v)	8.83	18.02	2.04	4.59	17.83	31.23	1.75	5.20
	CO_2/EtOH (80/20, *v*/v)	1.00	2.12	2.12	2.83	2.20	3.57	1.62	2.66
	CO_2/EtOH (85/15, *v*/v)	1.97	4.00	2.03	3.73	3.47	5.72	1.65	3.33
	CO_2/EtOH (90/10, *v*/v)	3.84	7.81	2.03	4.53	/	/	/	/
	CO_2/EtOH (95/5, *v*/v)	13.83	28.25	2.04	4.82	/	/	/	/
rac-**6b**	*n*-hexane/EtOH (80/20, *v*/v)	1.09	1.49	1.37	2.24	9.11	11.91	1.31	2.67
	DMC	0.45	0.56	1.24	0.65	5.42	9.81	1.81	4.70
	DMC/MeOH (90/10, *v*/v)	0.22	0.22	1.00	0.00	1.37	2.20	1.61	3.30
	DMC/EtOH (90/10, *v*/v)	0.27	0.27	1.00	0.00	1.91	3.10	1.62	3.45
	CO_2/MeOH (80/20, *v*/v)	0.58	0.87	1.51	0.82	4.17	6.61	1.58	3.34
	CO_2/MeOH (85/15, *v*/v)	0.94	1.38	1.47	1.18	7.22	11.44	1.58	3.87
	CO_2/MeOH (90/10, *v*/v)	1.58	2.32	1.47	1.72	12.95	20.87	1.61	4.35
	CO_2/MeOH (95/5, *v*/v)	3.73	5.58	1.49	2.69	/	/	/	/
	CO_2/MeOH (97/3, *v*/v)	6.98	10.81	1.55	3.01	/	/	/	/
	CO_2/EtOH (80/20, *v*/v))	0.59	0.79	1.34	0.58	6.00	9.07	1.51	3.21
	CO_2/EtOH (85/15, *v*/v)	1.18	1.53	1.30	0.93	9.13	14.04	1.54	3.64
	CO_2/EtOH (90/10, *v*/v)	2.01	2.68	1.33	1.49	/	/	/	/
	CO_2/EtOH (95/5, *v*/v)	4.96	6.97	1.40	2.54	/	/	/	/
rac-**6c**	*n*-hexane/EtOH (80/20, *v*/v)	1.39	1.44	1.04	1,72	6.61	8.34	1.26	2.13
	DMC	0.38	0.49	1.29	0.66	2.68	4.40	1.64	3.19
	DMC/MeOH (90/10, *v*/v)	0.20	0.20	1.00	0.00	0.76	1.12	1.48	2.06
	DMC/EtOH (90/10, *v*/v)	0.24	0.24	1.00	0.00	1.54	2.42	1.57	2.61
	CO_2/MeOH (80/20, *v*/v)	1.27	1.58	1.24	0.71	4.46	6.77	1.52	2.97
	CO_2/MeOH (85/15, *v*/v)	2.02	2.50	1.24	1.05	7.93	12.02	1.52	3.34
	CO_2/MeOH (90/10, *v*/v)	3.47	4.28	1.23	1.43	14.57	22.39	1.54	3.67
	CO_2/MeOH (95/5, *v*/v)	8.76	10.92	1.25	1.77	/	/	/	/
	CO_2/EtOH (80/20, *v*/v)	1.33	1.65	1.24	0.81	5.98	8.92	1.49	2.99
	CO_2/EtOH (85/15, *v*/v)	2.51	3.09	1.23	1.19	9.45	14.26	1.51	3.42
	CO_2/EtOH (90/10, *v*/v)	4.54	5.63	1.24	1.66	/	/	/	/
	CO_2/EtOH (95/5, *v*/v)	12.18	15.34	1.25	2.04	/	/	/	/
		**CHIRAL ART Amylose-SA**				**CHIRAL ART Cellulose-SC**			

Table S2. Chromatographic parameters for HPLC and SFC enantioseparation of *rac*-**8a-c**.

Marinoepoxide	Mobile phase	k_1	k_2	α	R_s	k_1	k_2	α	R_s
rac-**8a**	*n*-hexane/EtOH (80/20, *v*/v)	1.93	5.40	2.80	6.30	2.44	2.65	1.09	0.56
	DMC	1.27	1.72	1.36	1.56	3.97	4.90	1.23	1.49
	DMC/MeOH (90/10, *v*/v)	0.35	0.52	1.48	1.44	0.93	1.08	1.16	0.69
	DMC/EtOH (90/10, *v*/v)	0.44	0.62	1.42	1.29	2.10	2.49	1.19	1.24
	CO_2/MeOH (80/20, *v*/v)	4.09	12.75	3.11	6.33	9.16	10.14	1.11	0.74
	CO_2/MeOH (85/15, *v*/v)	6.58	19.86	3.02	6.32	17.22	19.30	1.12	0.98
	CO_2/MeOH (90/10, *v*/v)	12.03	35.31	2.94	6.50	5.40	6.11	1.13	0.77
	CO_2/EtOH (80/20, *v*/v)	3.88	8.22	2.12	4.58	8.72	9.92	1.14	0.98
	CO_2/EtOH (85/15, *v*/v)	6.54	13.78	2.11	5.01	3.93	4.27	1.09	0.63
	CO_2/EtOH (90/10, *v*/v)	13.36	28.35	2.12	5.03	/	/	/	/
rac-**8b**	*n*-hexane/EtOH (80/20, *v*/v)	1.96	5.73	2.92	8.74	11.72	13.16	1.12	1.35
	DMC	0.40	0.64	1.61	1.92	3.77	4.83	1.28	1.85
	DMC/MeOH (90/10, *v*/v)	0.19	0.33	1.71	1.38	1.15	1.35	1.17	0.97
	DMC/EtOH (90/10, *v*/v)	0.22	0.36	1.91	1.45	1.51	1.81	1,20	1.17
	CO_2/MeOH (80/20, *v*/v)	2.66	6.45	2.43	5.60	9.96	11.51	1.16	1.25
	CO_2/MeOH (85/15, *v*/v)	4.11	9.79	2.38	6.16	17.10	19.80	1.16	1.28
	CO_2/MeOH (90/10, *v*/v)	6.88	16.32	2.37	6.45	30.41	35.41	1.16	1.54
	CO_2/EtOH (80/20, *v*/v)	2.31	4.54	1.97	4.36	12.50	14.34	1.15	1.55
	CO_2/EtOH (85/15, *v*/v)	4.22	8.09	1.92	5.11	19.39	22.34	1,15	1.41
	CO_2/EtOH (90/10, *v*/v)	7.62	14.74	1.93	5.86	/	/	/	/
rac-**8c**	*n*-hexane/EtOH (80/20, *v*/v)	1.89	5.19	2.75	7.23	9.31	9.31	1.00	0.00
	DMC	0.31	0.51	1.63	1.45	0.68	0.68	1.00	0.00
	DMC/MeOH (90/10, *v*/v)	0.17	0.25	1.46	0.68	0.69	0.69	1.00	0.00
	DMC/EtOH (90/10, *v*/v)	0.19	0.30	1.55	0.87	0.87	0.87	1.00	0.00
	CO_2/MeOH (80/20, *v*/v)	4.45	8.22	1.85	4.53	11.93	11.93	1.00	0.00
	CO_2/MeOH (85/15, *v*/v)	7.00	12.73	1.82	4.82	21.04	21.04	1.00	0.00
	CO_2/MeOH (90/10, *v*/v)	12.17	22.18	1.75	5.22	/	/	/	/
	CO_2/EtOH (80/20, *v*/v)	4.00	7.20	1.80	4.58	14.11	15.62	1.11	0.72
	CO_2/EtOH (85/15, *v*/v)	7.41	13.21	1.78	5.18	22.85	25.69	1.12	0.75
	CO_2/EtOH (90/10, *v*/v)	14.14	25.32	1.79	5.92	/	/	/	/
		CHIRAL ART Amylose-SA				*CHIRAL ART Cellulose-SC*			

References:

[1] B. S. Furniss, Antony J. Hannaford, Peter W. G. Smith, Austin R. Tatchell, *Vogel´s textbook of practical organic chemistry*, Fifth edition, London, **1989**, 902.

[2] A. S. C. Fonseca, M. S. T. Gonçalves, S. P. G. Costa, *Amino Acids,*
2010, *39*, 699-712.

[3] K. Manpreet, B. S. Jayashree, P. Aravinda, *Pharmanest*, **2014**, *5*, 1724-1732.

[4] T. Durst, R. N. Ben, M.-N- Roy, e-EROS Encyclopedia of Reagents for Organic Synthesis **2013**, 1-3.
